# Supplementary material for: Counteranion-mediated efficient iodine capture in a hexacationic imidazolium organic cage enabled by multiple non-covalent interactions
Source: Nat Commun. 2023 Sep 28;14:6082. doi: 10.1038/s41467-023-41866-7 (PMC10539326; doi:10.1038/s41467-023-41866-7)
Supplement: Supplementary file 1 — Supplementary information [file 41467_2023_41866_MOESM1_ESM.pdf]

Supplementary Information

Counteranion-Mediated Efficient Iodine Capture in a Hexacationic Imidazolium Organic Cage Enabled by Multiple Non-covalent Interactions

Jian Yang,[1] Shao-Jun Hu,[1] Li-Xuan Cai,[1] Li-Peng Zhou[1] and Qing-Fu Sun[1,2]*

[1] State Key Laboratory of Structure Chemistry, Fujian Institute of Research on the Structure of Matter, Chinese Academy of Sciences, Fuzhou, Fujian, 350002 (China)

[2] University of Chinese Academy of Sciences, Beijing, 100049 (China)

*Correspondence to: qfsun@fjirsm.ac.cn.

Table of Contents

1. Supplementary methods 2
    1.1 Synthesis and characterization 2
    1.2 Iodine vapor capture experiments 12
    1.3 Iodine uptake experiments from solution 13
    1.4 Recyclability experiments of 3·6Br for I_2 capture 14
    1.5 Single Crystal X-Ray Diffraction Study 15
2. Supplementary figures 15
    2.1 Supplementary mass spectra 15
    2.2 Supplementary X-ray crystal structures 17
    2.3 Supplementary PXRD spectra 22
    2.4 Supplementary N_2 adsorption isotherm 24
    2.5 Supplementary TGA spectra 26
    2.6 Supplementary UV/vis absorption spectra 30
    2.7 Supplementary FT-IR spectra 34
    2.8 Supplementary Raman spectra 36
    2.9 Supplementary XPS spectra 37
    2.10 Supplementary ^1H NMR titration experiments spectra 41
3. Supplementary tables 43
4. Supplementary references 48

1. Supplementary methods

1.1 Synthesis and characterization

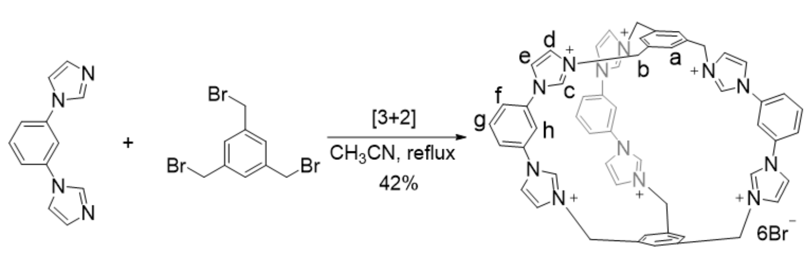

Supplementary Fig. 1 Synthesis of **3**·6Br.

Precursor **1** was synthesized from a reported literature.[1] To 100 mL acetonitrile solution of **1** (50 mg, 0.24 mmol, 1.5 equiv) in a 250 mL flame-dried round bottom flask was added **2** (57 mg, 0.16 mmol, 1 equiv) in 100 mL acetonitrile dropwise. The reaction mixture was stirred at reflux temperature for 2 d to give rise to pure **3**·6Br as a white precipitate from the reaction solution, which was recrystallized from water, collected and dried (45 mg, 42% yield). ^{1}H NMR (400 MHz, DMSO-d_6) δ 10.19 (s, 6H), 8.53 (s, 6H), 8.24 (s, 3H), 8.19 (s, 6H), 8.02 – 7.91 (m, 9H), 7.77 (s, 6H), 5.56 (s, 12H). ^{13}C NMR (151 MHz, DMSO-d_6) δ 136.3, 136.0, 135.9, 132.4, 131.3, 124.6, 122.7, 121.5, 116.4, 52.8. HR-ESI-TOF-MS for **3**·6Br: m/z calcd. for $[M-2Br^-]^{2+}$: 592.0408, found 592.0398.

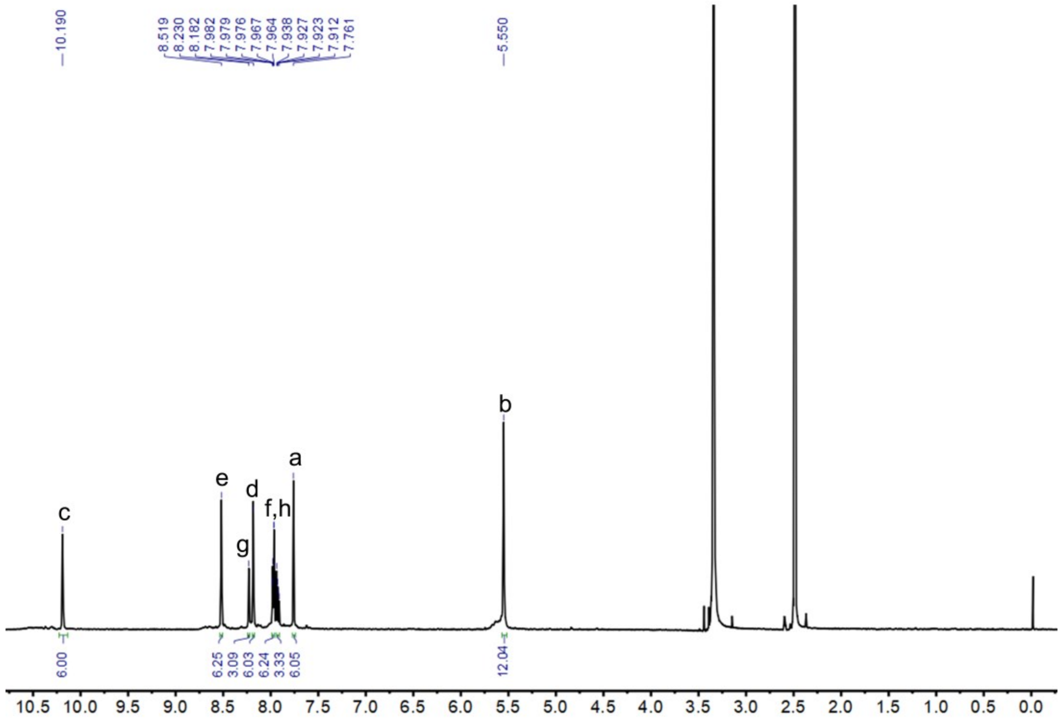

Supplementary Fig. 2 ^{1}H NMR spectrum of **3**·6Br (400 MHz, DMSO-d_6, 298 K).

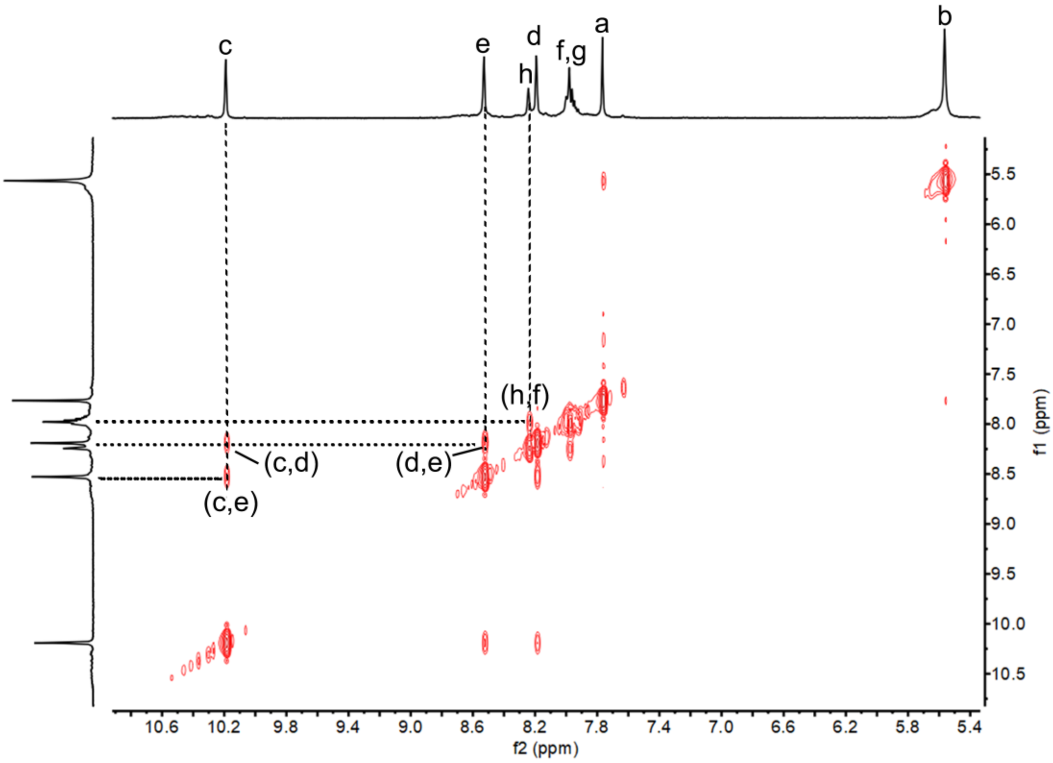

Supplementary Fig. 3 ^{1}H-^{1}H COSY NMR spectrum of **3**·6Br (400 MHz, DMSO-d_6, 298 K).

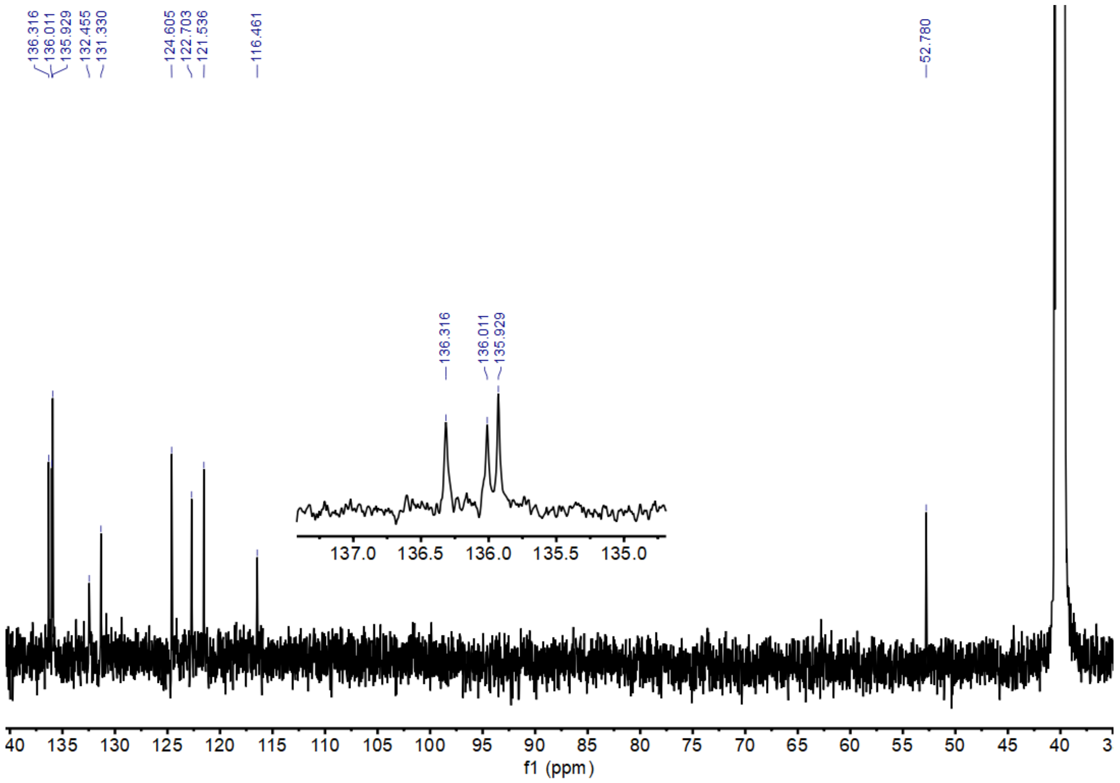

Supplementary Fig. 4 ^{13}C NMR spectrum of **3**·6Br (151 MHz, DMSO-d_6, 298 K).

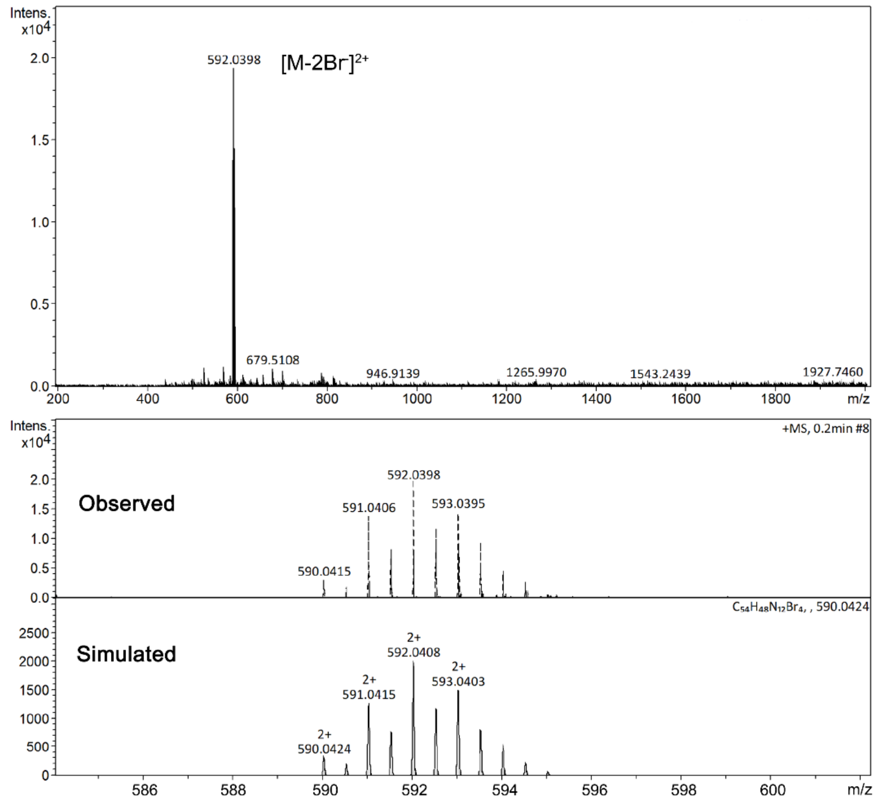

Supplementary Fig. 5 HR-ESI-TOF-MS of **3**·6Br and the observed and calculated isotopic patterns for [M-2Br$^-$]$^{2+}$.

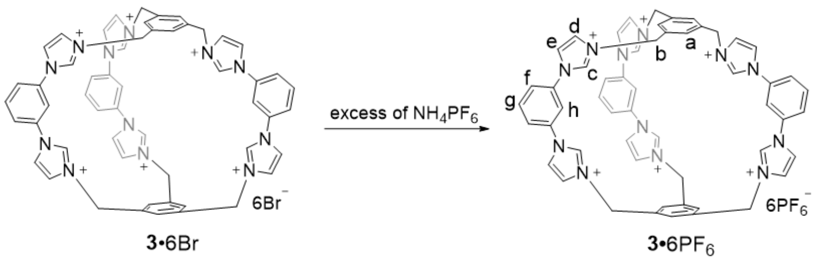

Supplementary Fig. 6 Synthesis of **3**·6PF$_6$.

To a solution of **3**·6Br (50 mg, 0.037 mmol) in 50 mL distilled water was added excess of NH_4PF_6, the reaction mixture was heated at 80 °C overnight. White solid precipitated out from reaction solution, which was collected and washed with distilled water, dried under vacuum to give **3**·6PF$_6$ as a white solid (58 mg, 90% yield). ^{1}H NMR (600 MHz, DMSO-d_6) δ 9.64 (s, 6H), 8.25 (s, 6H), 8.22 (s, 6H), 8.01 – 7.94 (m, 9H), 7.72 (s, 3H), 7.68 (s, 6H), 5.56 (s, 12H). ^{13}C NMR (101 MHz, DMSO-d_6) δ 137.1, 135.7, 135.5, 133.1, 129.5, 124.7, 123.3, 121.9, 116.0, 52.6. HR-ESI-TOF-MS for **3**·6PF$_6$: m/z calcd. for $[M-2PF_6^-]^{2+}$: 722.1341, found 722.1329.

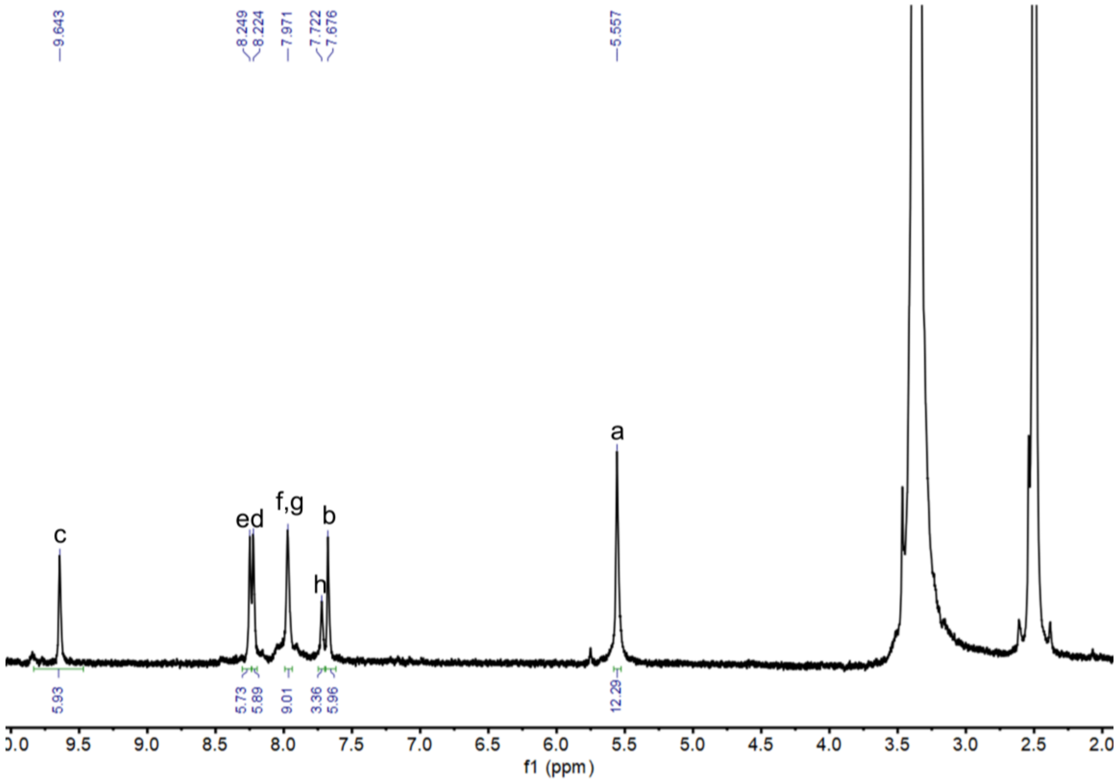

Supplementary Fig. 7 ^{1}H NMR spectrum of **3**·6PF$_6$ (600 MHz, DMSO-d_6, 298 K).

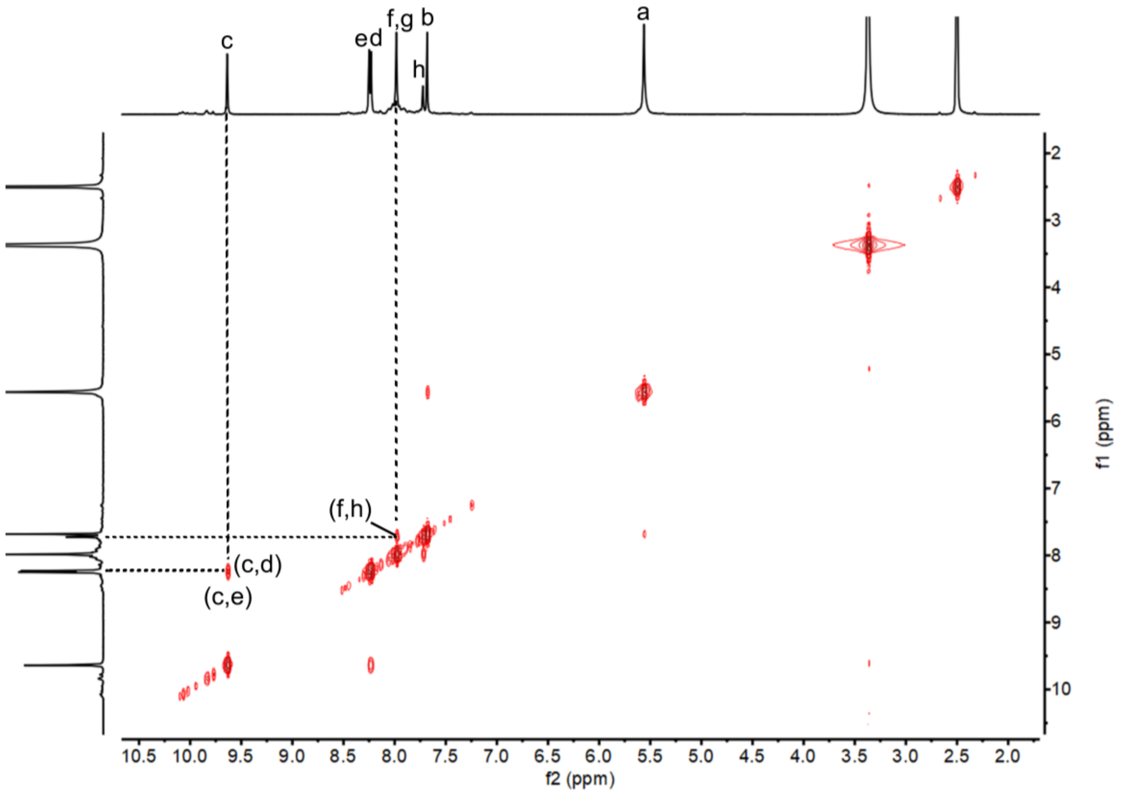

Supplementary Fig. 8 ^{1}H-^{1}H COSY NMR spectrum of **3**·$6PF_6$ (400 MHz, DMSO-d_6, 298 K).

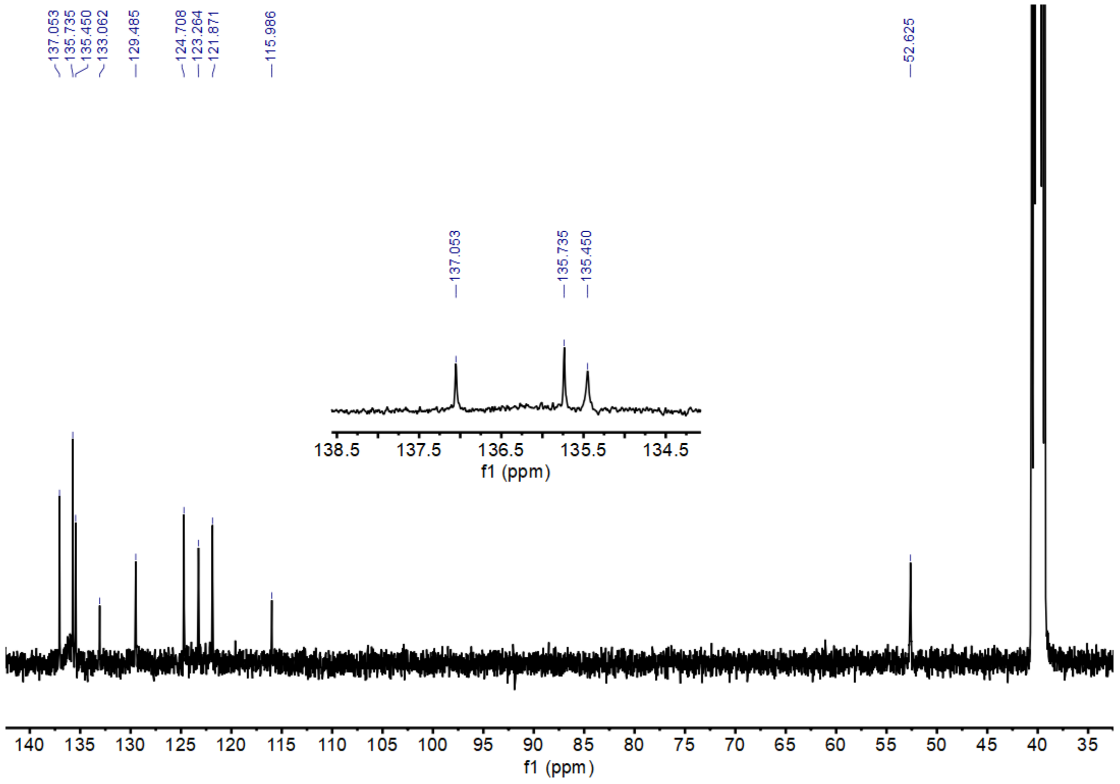

Supplementary Fig. 9 ^{13}C NMR spectrum of **3**·$6PF_6$ (101 MHz, DMSO-d_6, 298 K).

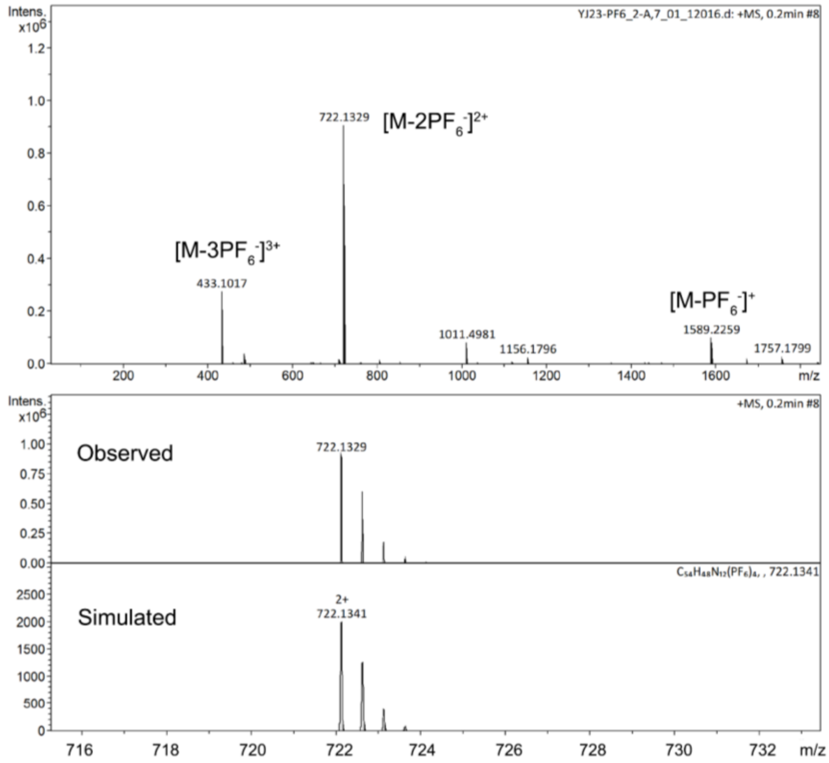

Supplementary Fig. 10 HR-ESI-TOF-MS of **3**·6PF$_6$ and the observed and calculated isotopic patterns for [M-2PF$_6^-$]$^{2+}$.

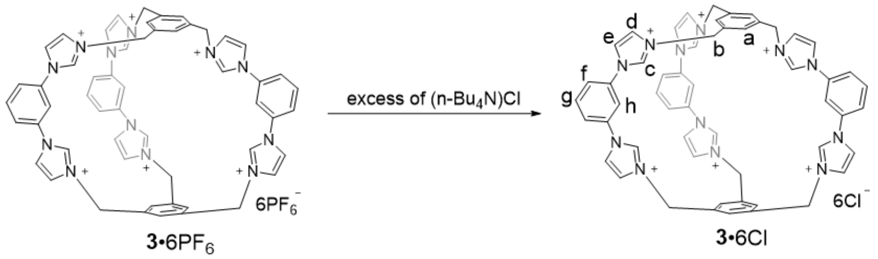

Supplementary Fig. 11 Synthesis of **3**·6Cl.

To a solution of **3**·6PF$_6$ (50 mg, 0.029 mmol) in 50 mL acetonitrile was added excess of (n-Bu$_4$N)Cl, the reaction mixture was heated at 80 °C overnight. White solid precipitated out from reaction solution, which was collected and washed with distilled water, dried under vacuum to give **3**·6Cl as a white solid (30 mg, 95% yield). ^{1}H NMR (600 MHz, DMSO-*d*$_6$) δ 10.29 (s, 6H), 8.62 (t, *J* = 1.9 Hz, 6H), 8.28 – 8.26 (m, 3H), 8.21 (t, *J* = 1.9 Hz, 6H), 7.95 – 7.93 (m, 6H), 7.89 – 7.87 (m, 3H), 7.70 (s, 6H), 5.50 (s, 12H). ^{13}C NMR (101 MHz, DMSO-*d*$_6$) δ 136.3, 135.9, 135.8, 132.3, 131.3, 124.5, 122.6, 121.5, 116.5, 52.7. HR-ESI-TOF-MS for **3**·6Cl: m/z calcd. for [M-2Cl$^-$]$^{2+}$: 503.1425, found 503.1406.

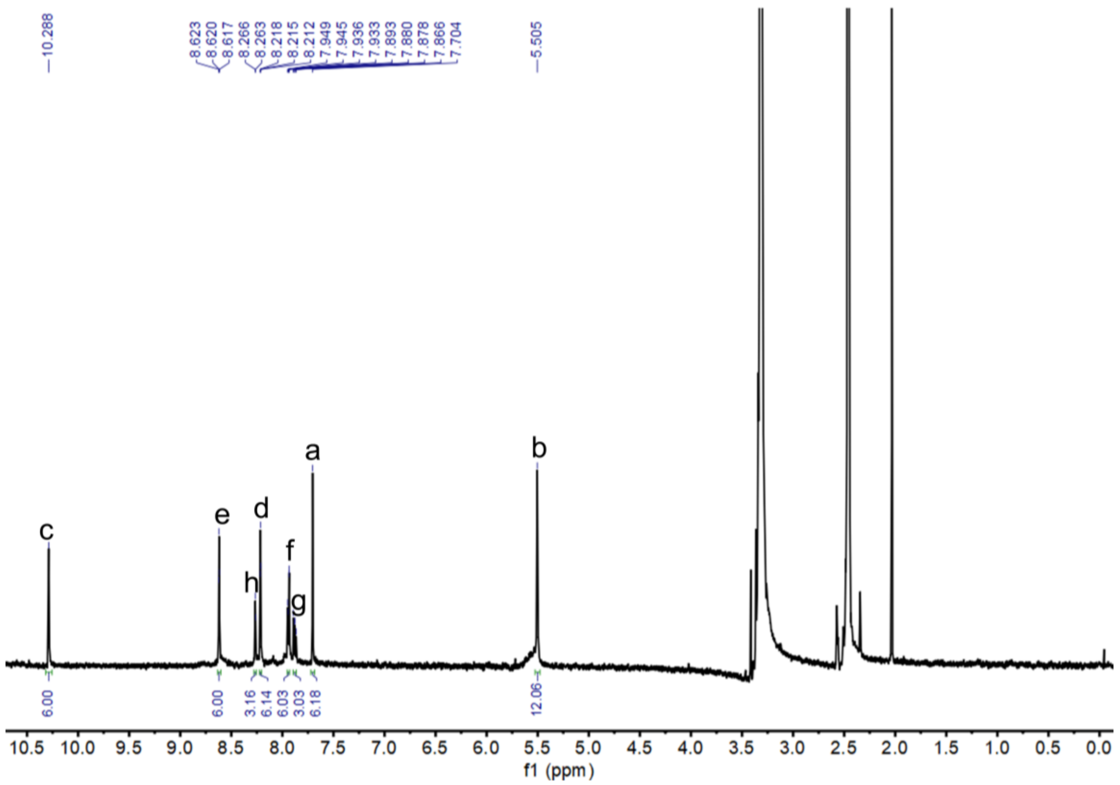

Supplementary Fig. 12 ^{1}H NMR spectrum of **3**·6Cl (600 MHz, DMSO-d_6, 298 K).

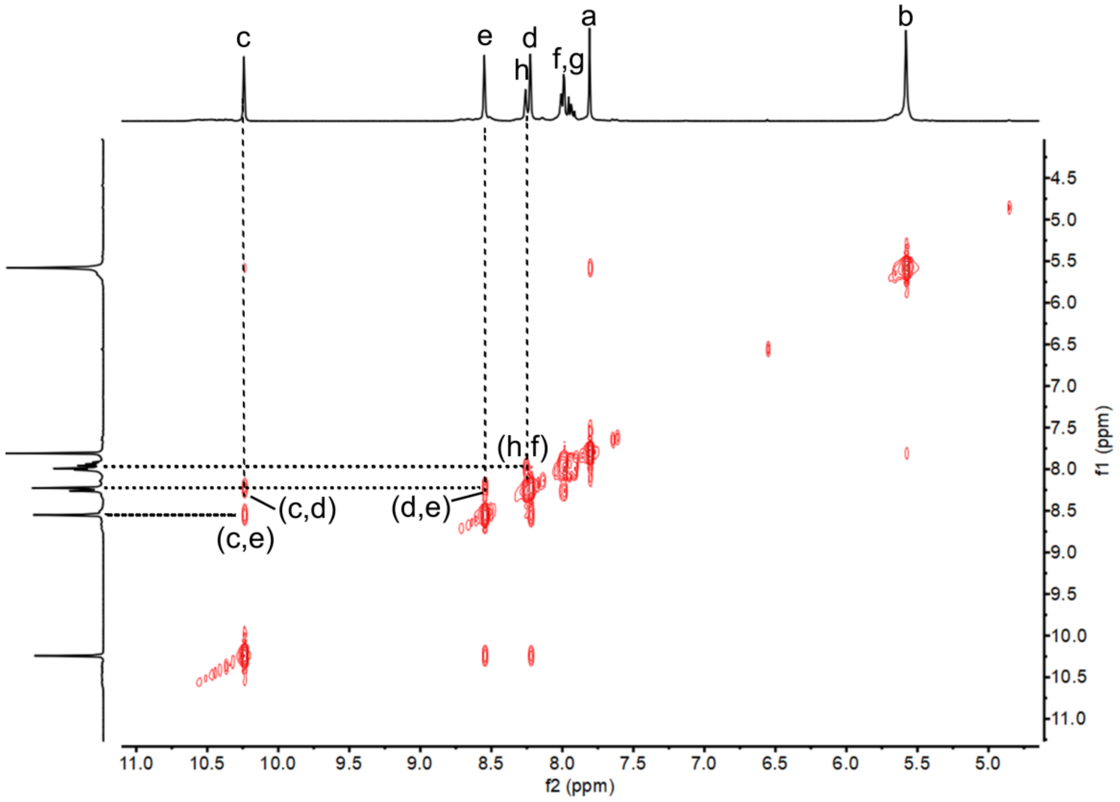

Supplementary Fig. 13 ^{1}H-^{1}H COSY NMR spectrum of **3**·6Cl (400 MHz, DMSO-d_6, 298 K).

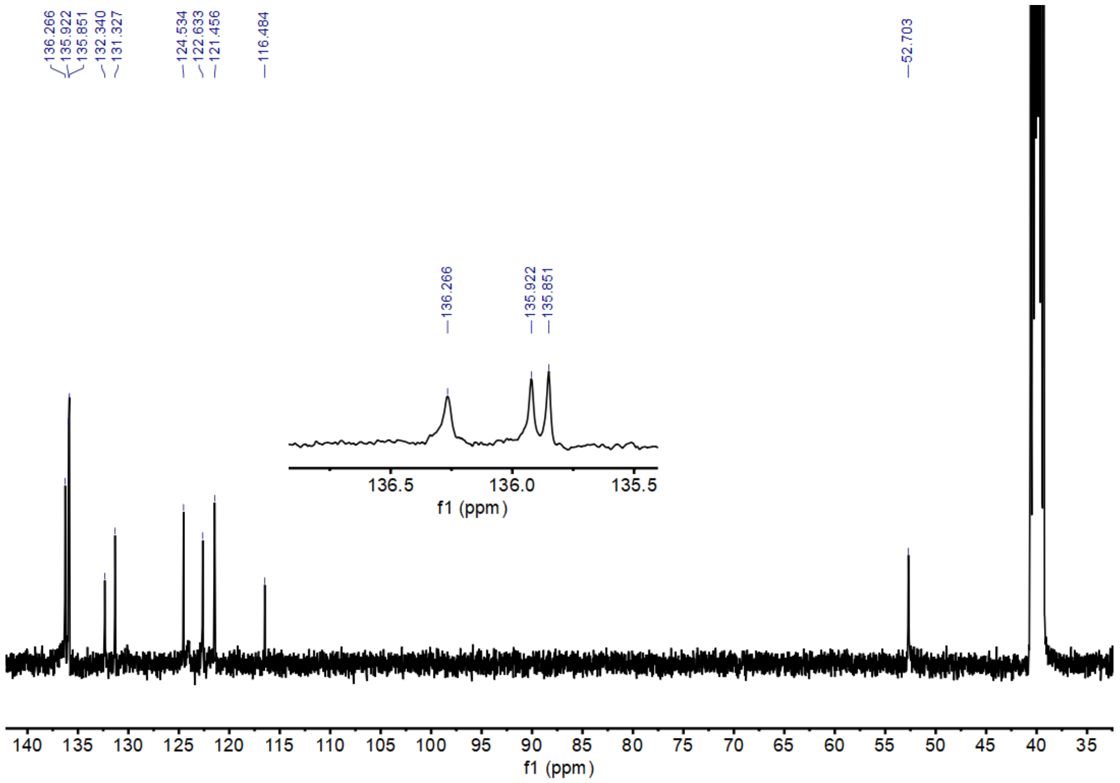

Supplementary Fig. 14 ^{13}C NMR spectrum of **3**·6Cl (101 MHz, DMSO-*d*$_6$, 298 K).

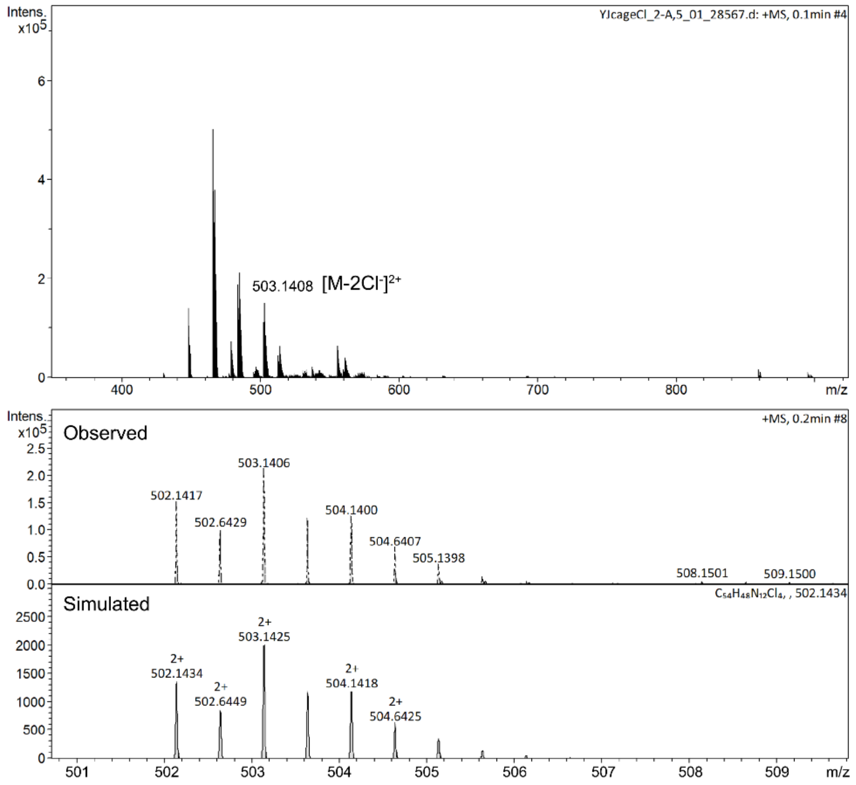

Supplementary Fig. 15 HR-ESI-TOF-MS of **3**·6Cl and the observed and calculated isotopic patterns for [M-2Cl^{-}]$^{2+}$.

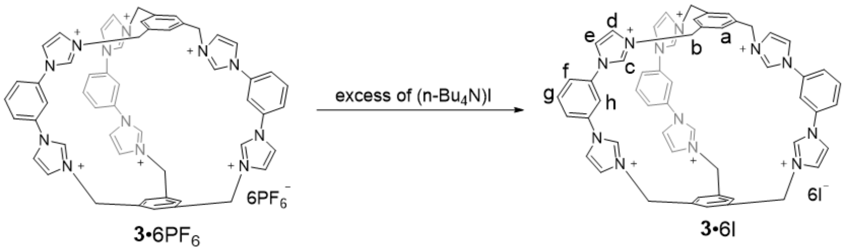

Supplementary Fig. 16 Synthesis of **3**·6I.

Synthesis of **3**·6I was similar to that for **3**·6Cl by using (n-Bu_4N)I instead of (n-Bu_4N)Cl. **3**·6I was obtained as a white solid. ^{1}H NMR (600 MHz, DMSO-d_6) δ 9.91 (s, 6H), 8.36 – 8.29 (m, 6H), 8.22 – 8.15 (m, 6H), 7.99 – 7.97 (m, 3H), 7.96 – 7.91 (m, 9H), 7.69 (s, 6H), 5.52 (s, 12H). ^{13}C NMR (151 MHz, DMSO-d_6) δ 136.5, 135.9, 135.9, 132.8, 130.5, 124.6, 123.1, 121.8, 116.1, 52.7. HR-ESI-TOF-MS for **3**·6I: m/z calcd. for $[M-2I^-]^{2+}$: 686.0146, found 686.0143.

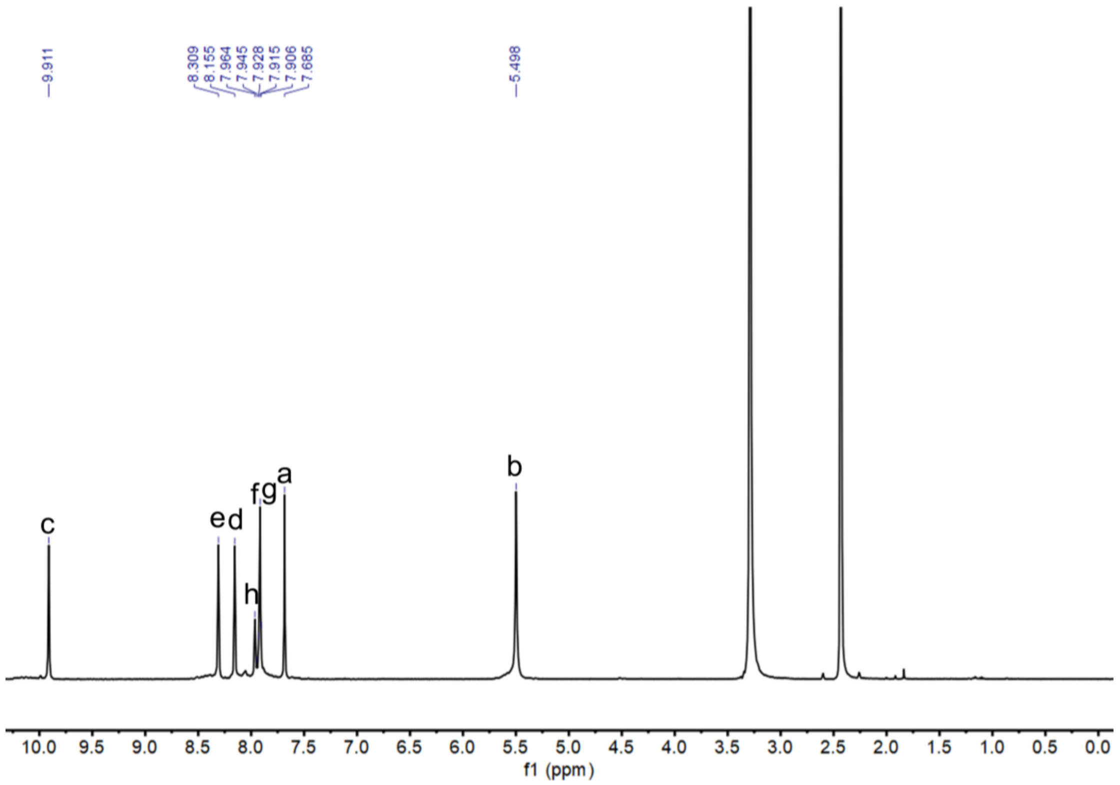

Supplementary Fig. 17 ^{1}H NMR spectrum of **3**·6I (600 MHz, DMSO-d_6, 298 K).

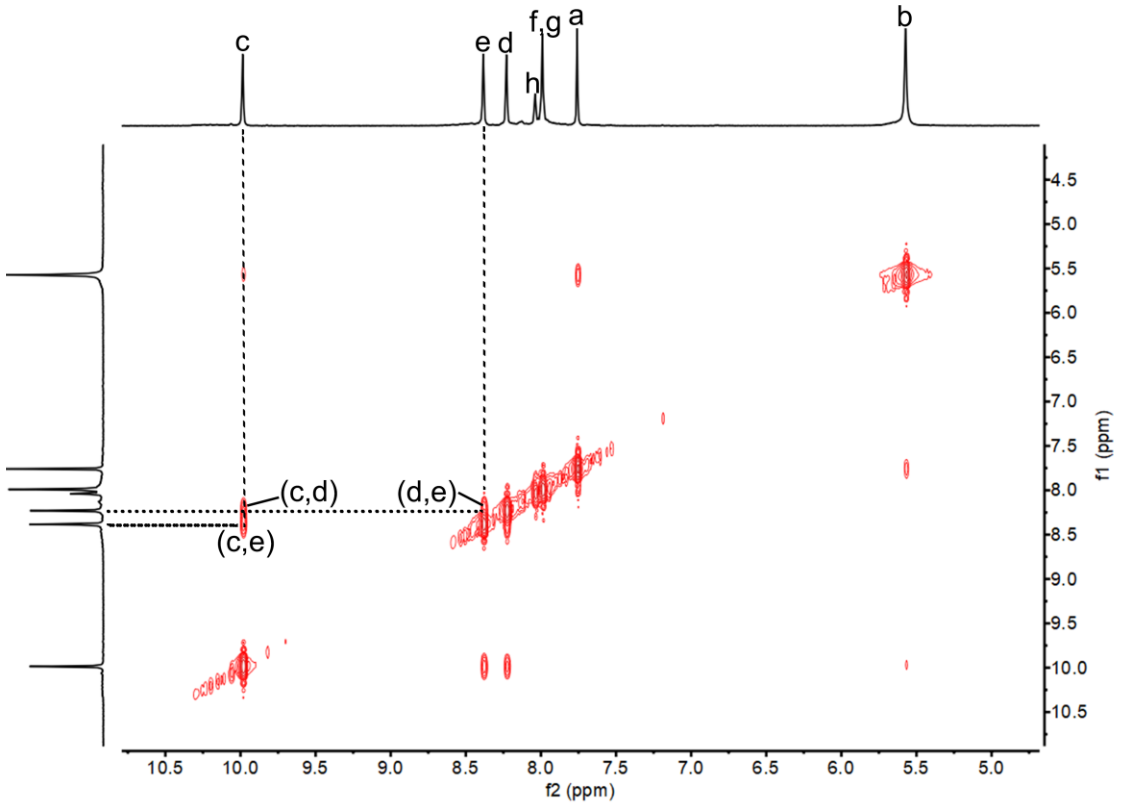

Supplementary Fig. 18 ^{1}H-^{1}H COSY NMR spectrum of **3**·6I (400 MHz, DMSO-d_6, 298 K).

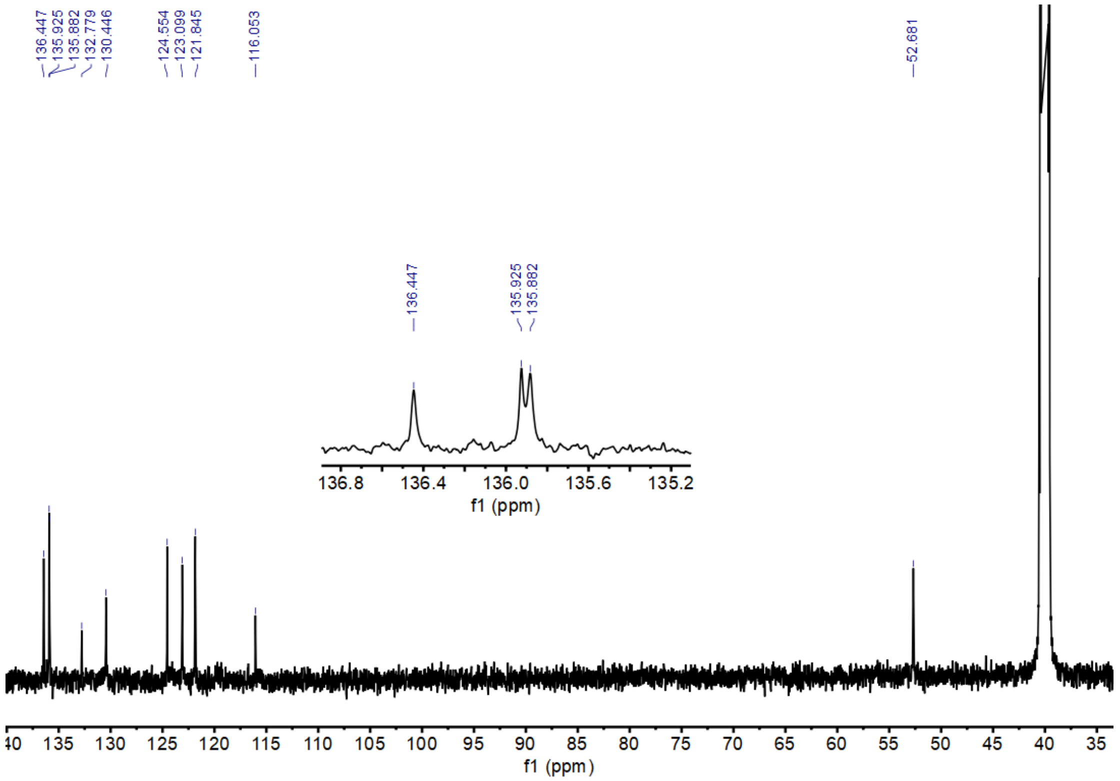

Supplementary Fig. 19 ^{13}C NMR spectrum of **3**·6I (151 MHz, DMSO-d_6, 298 K).

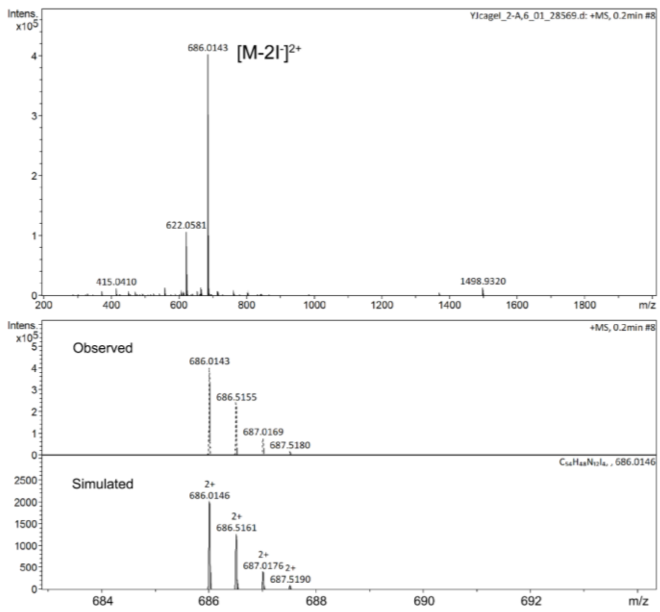

Supplementary Fig. 20 HR-ESI-TOF-MS of **3**·6I and the observed and calculated isotopic patterns for $[M\text{-}2I^{-}]^{2+}$.

1.2 Iodine vapor capture experiments

Before iodine capture experiments, the adsorbents were activated by heating at 120 ^{o}C under vacuum for 12 h. A typical iodine vapor capture experiment was carried out as follows: to a pre-weighed glass vial was placed 10 mg activated adsorbent **3**·6X (X = Cl^{-}, Br^{-}, I^{-}, PF_6^{-}), and the glass vial was then transferred to a sealed jar containing excessive iodine at the bottom. The sealed jar was placed in an oven at 75 ^{o}C. At different time intervals, the iodine-adsorbed sample was cooled down to room temperature and weighed by analytical balance.

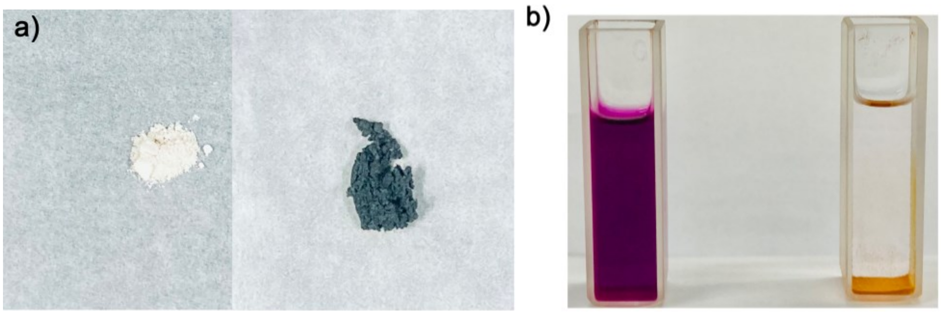

Supplementary Fig. 21 Photographs for (a) **3**·6Cl before and after iodine adsorption. (b) A solution of I_2 in n-hexane (2 mM, 3 mL) upon addition of 5 mg **3**·6I for 5 minutes.

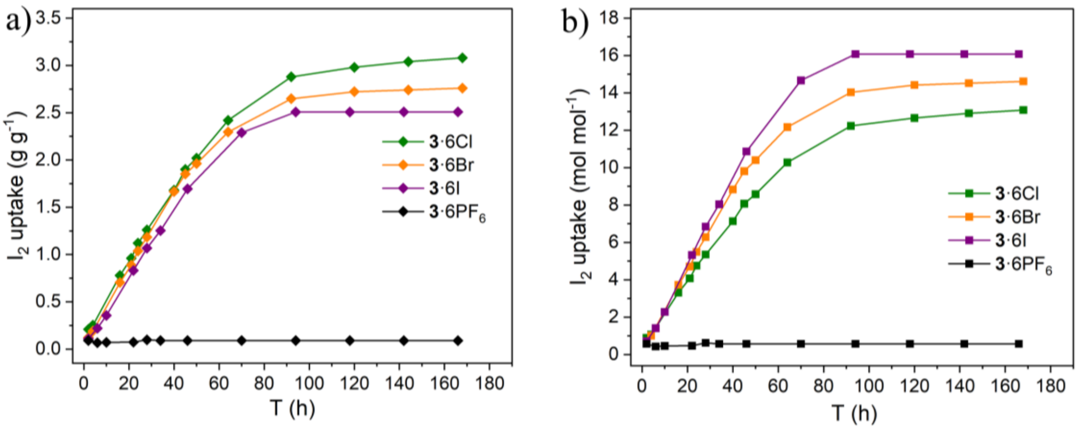

Supplementary Fig. 22, Time-dependent I_2 vapor uptake by **3**·6X (X = Cl^-, Br^-, I^-, PF_6^-) in (a) g g^{-1}, and (b) mol mol^{-1} at room temperature, respectively.

1.3 Iodine uptake experiments from solution

A solution of I_2 in n-hexane (2 mM) was prepared and time-dependent UV/vis absorption spectra of a solution of I_2 in n-hexane (2 mM, 3 mL) upon addition of 5 mg **3**·6X (X = Cl^-, Br^-, I^-, PF_6^-) was were recorded at various time.

For determining the adsorption capacity of I_2 from n-hexane, and given that **3**·6I exhibits the fastest I_2 adsorption rate from n-hexane among **3**·6X (X=Cl^-, Br^-, I^-, PF_6^-), therefor, **3**·6I was selected as absorbent for evaluating its I_2 adsorption capacity from n-hexane. Firstly, we made a calibration plot of standard for iodine in n-hexane solution by UV-vis spectra. And then 10 mg **3**·6I was placed in a close-sealed glass bottle containing 2.5 mg/mL I_2 solution in n-hexane. And the solution was stirred for 24 h. After that the stirring was stopped and the solution was kept still for 30 min. The supernatant was diluted and used for UV-vis absorbance measurement. According to the standard calibration plot, the iodine adsorption from n-hexane for **3**·6I was calculated to be 2.26 ± 0.02 g g^{-1}, which was less than that from vapor (3.54 g g^{-1}), which might be due to occupation of pores in **3**·6I by solvent molecules.

An aqueous solution of I_2/KI was chosen as the iodine source considering the low solubility of I_2 in water, and meanwhile this system can supply an effective source of I^-, I_2 and I_3^-.[2-3] An aqueous solution of I_2/KI was prepared by mixing equal mole of I_2 and KI in water. Similarly, the iodine adsorption capacity from aqueous solution of

I_2/KI by **3**·6PF_6 was determined to be 1.10 ± 0.17 g g^{-1}.

1.4 Recyclability experiments of 3·6Br for I_2 capture

Immersing I_2-adsorbed sample I_2@**3**·6Br into ethanol on sonication, after several times the desorption was finished until ethanol solution became colorless. And then the recycled **3**·6Br was reactivated by heating at 120 °C under vacuum for 12 h for recyclability experiments of I_2 capture as described above.

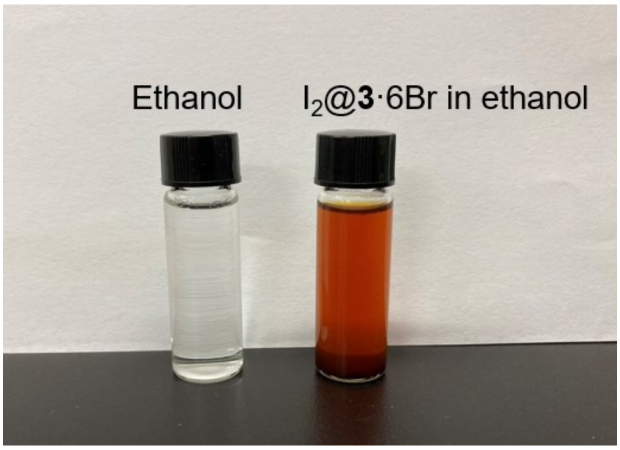

Supplementary Fig. 23 Photographs of I_2 desorption from I_2@3·6Br in ethanol (first immersing).

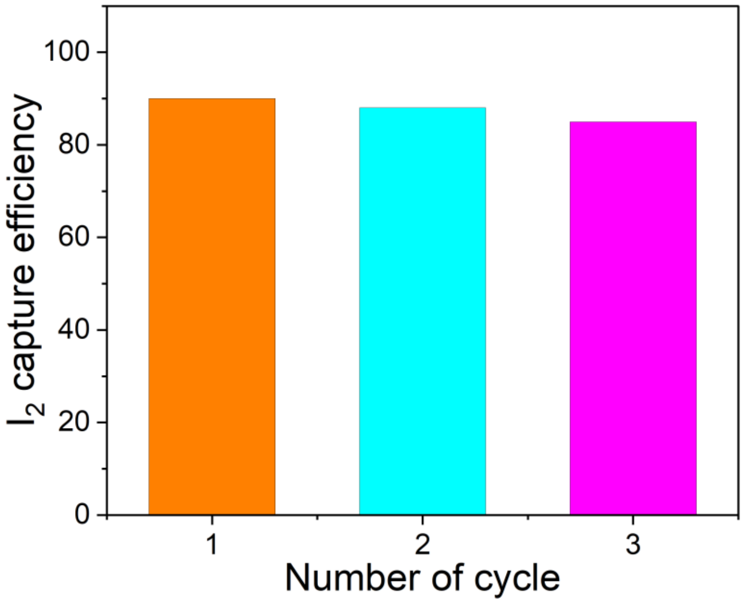

Supplementary Fig. 24 Recyclability experiments of **3**·6Br for I_2 capture.

1.5 Single Crystal X-Ray Diffraction Study

Suitable single crystals of **3**·6Cl and **3**·6Br were obtained by slow evaporation of their aqueous solution for one month, single crystals of **3**·6I and **3**·$6PF_6$ were obtained by slow vapor diffusion of diethyl ether into their corresponding DMSO solution for three weeks. Single crystal of **3**·$[I_2Br]_5[I_4Br]$ was obtained by slow evaporation of corresponding solution of **3**·6Br with I_2 in acetonitrile for one week. The X-ray diffraction for **3**·6Cl, **3**·6I, **3**·$6PF_6$ and **3**·$[I_2Br]_5[I_4Br]$ were carried out on micro-focus metaljet diffractometer using Ga Kα radiation (λ = 1.3405 Å). Data reduction was performed with the CrysAlisPro package.[4] The X-ray diffraction for **3**·6Br were carried out on Bruker D8 VENTURE photon II diffractometer with Iμs 3.0 microfocus X-ray source using APEX III program. And an analytical absorption correction was performed. Data reduction was performed with the saint and SADABS package.[5] The structures were solved by direct methods and refined by full-matrix least-squares on F^2 with anisotropic displacement using the SHELX software package.[6] The large amount of amorphous solvents and highly-disordered counterions existing in the unit cell, these residual electron intensities were removed by the PLATON/SQUEEZE routine.[7] Details on crystal data collection and refinement were summarized in Supplementary Tables 2-6. CCDC 2234458-2234462 contains the supplementary crystallographic data for this paper. These data can be obtained free of charge via www.ccdc.cam.ac.uk/conts/retrieving.html (or from the Cambridge Crystallographic Data Centre, 12 Union Road, Cambridge CB21EZ, UK; fax: (+44) 1223-336-033; or deposit@ccdc.cam.ac.uk).

2. Supplementary figures

2.1 Supplementary mass spectra

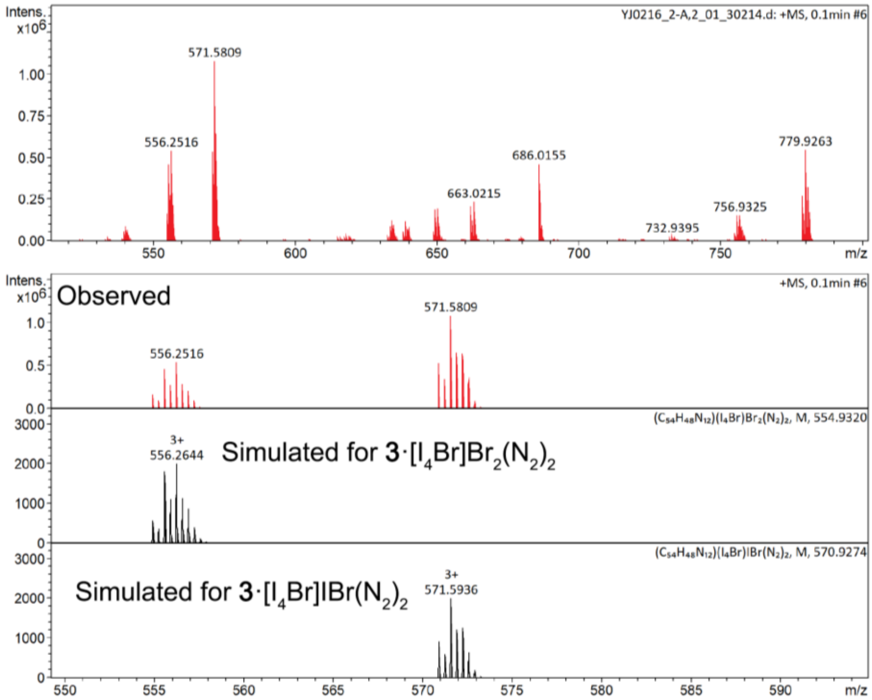

Supplementary Fig. 25 Experimental and simulated patterns of mass spectra for [**3**·$(I_4Br)Br_2(N_2)_2]^{3+}$ and [**3**·$(I_4Br)IBr(N_2)_2]^{3+}$.

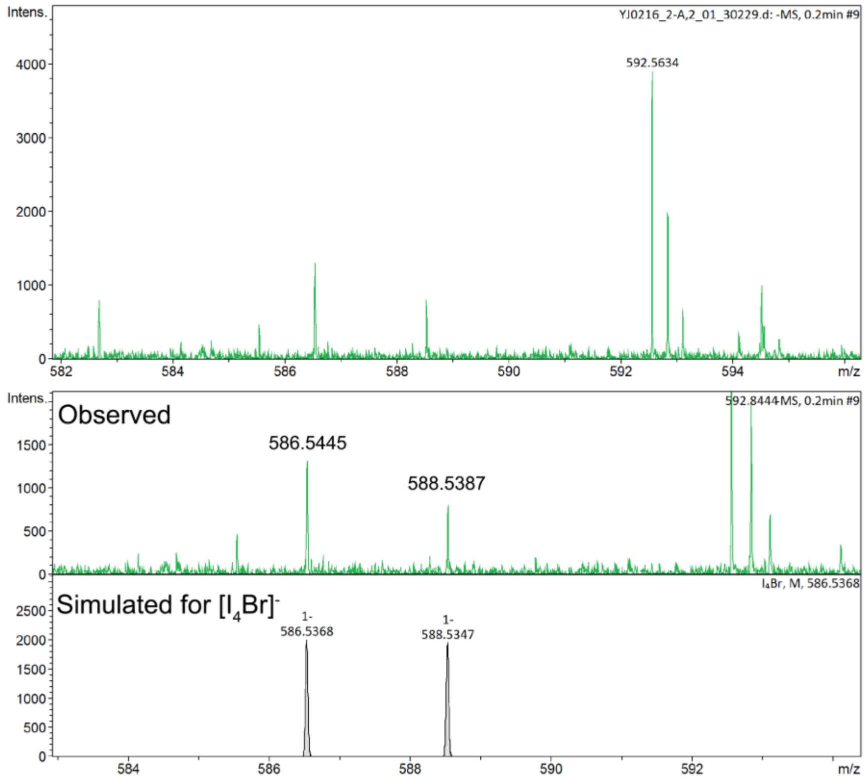

Supplementary Fig. 26 Experimental and simulated patterns of mass spectra for $[I_4Br]^{-}$.

2.2 Supplementary X-ray crystal structures

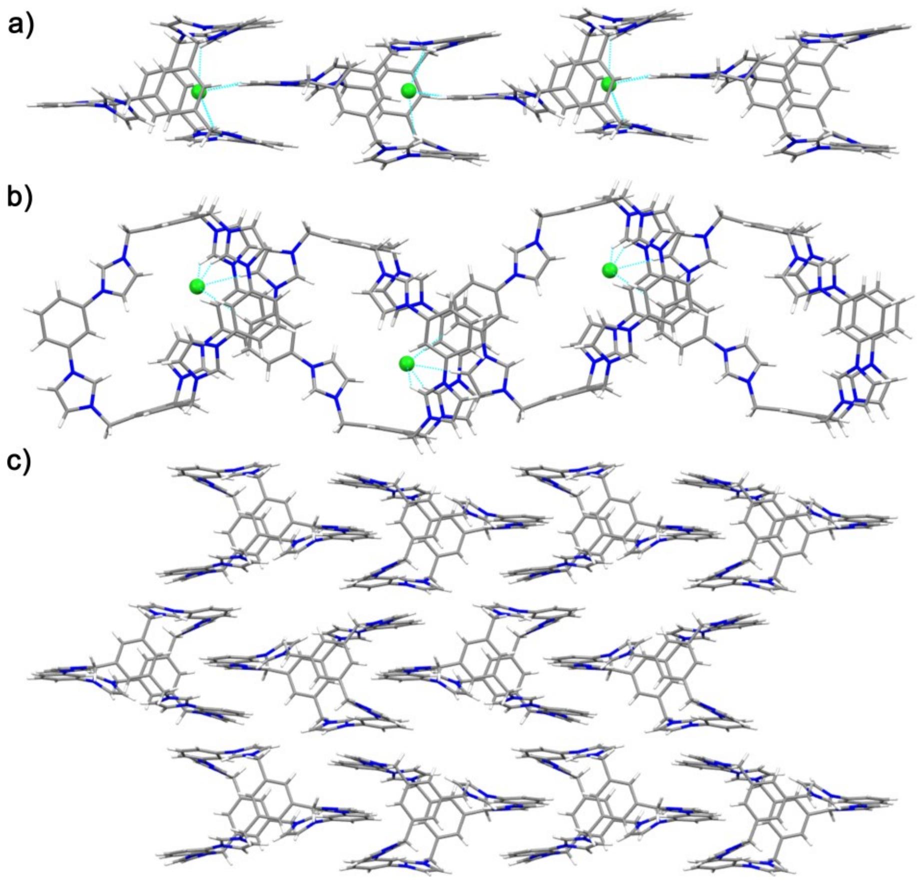

Supplementary Fig. 27 Crystal structure of **3**·6Cl. (a) Top view and (b) side view of 1D chain connected by C-H···Cl hydrogen bonds. (c) Stacked 3D supramolecular structure. Solvent molecules and unrelated anions are omitted for clarity.

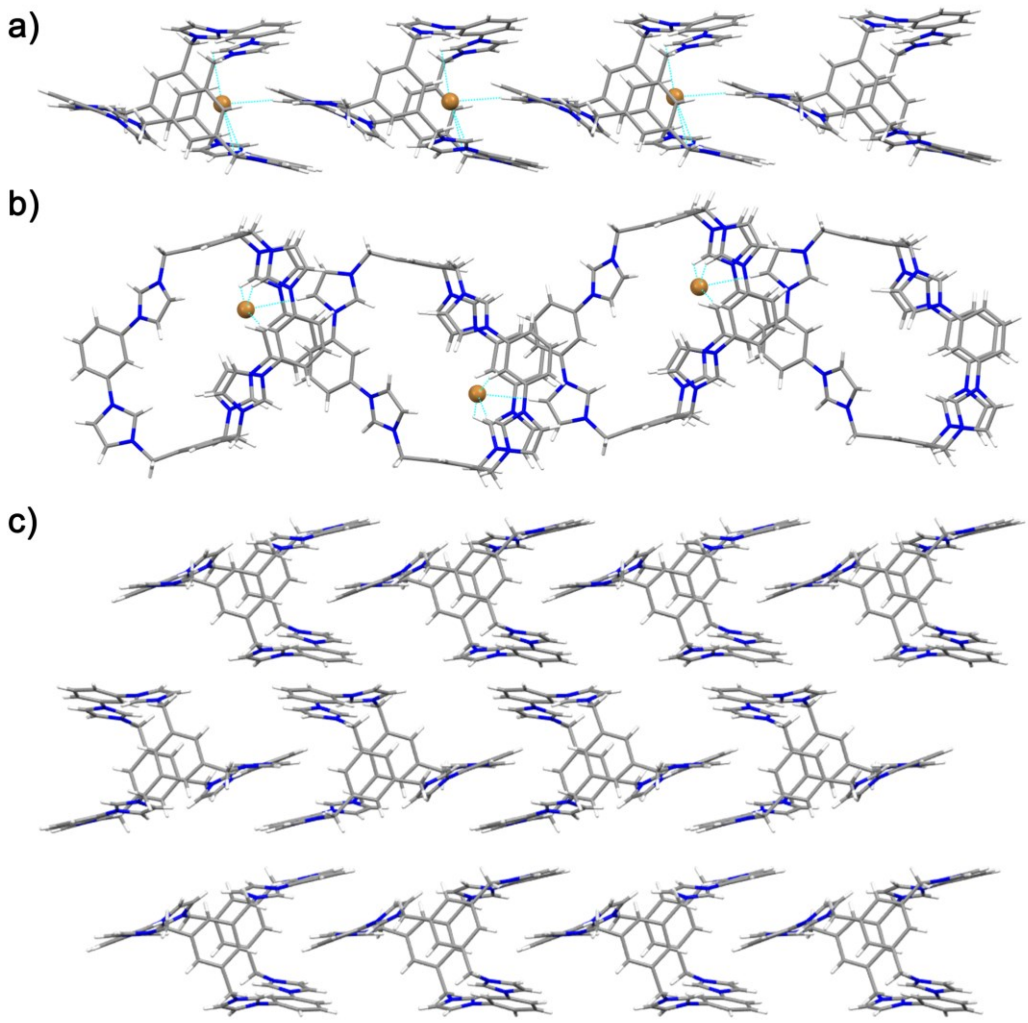

Supplementary Fig. 28 Crystal structure of **3**·6Br. (a) Top view and (b) side view of 1D chain connected by C-H···Br hydrogen bonds. (c) Stacked 3D supramolecular structure. Solvent molecules and unrelated anions are omitted for clarity.

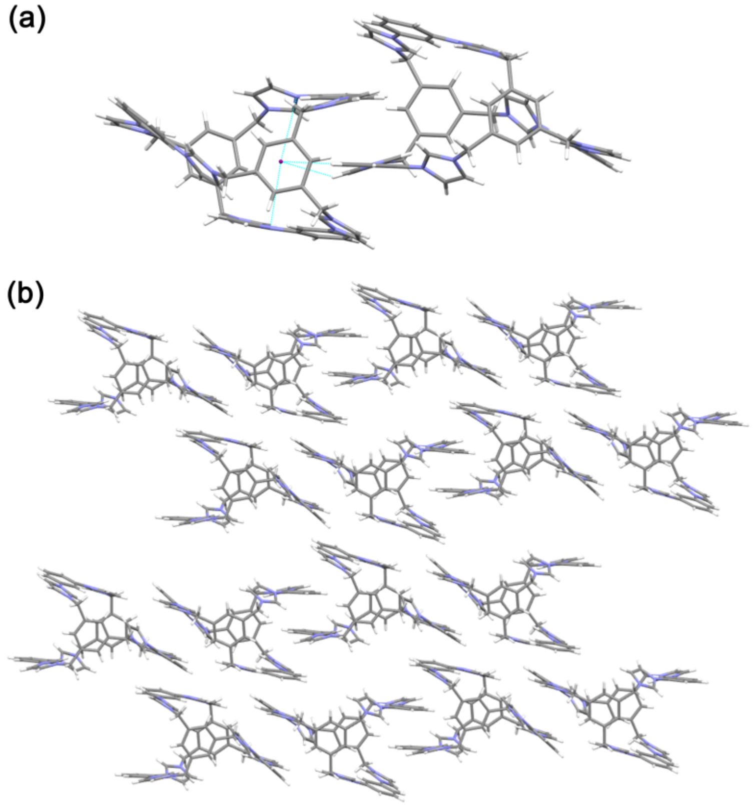

Supplementary Fig. 29 Crystal structure of **3**·6I. (a) Top view of dimer connected by C-H···I hydrogen bonds. (b) Stacked 3D supramolecular structure. Solvent molecules and unrelated anions are omitted for clarity.

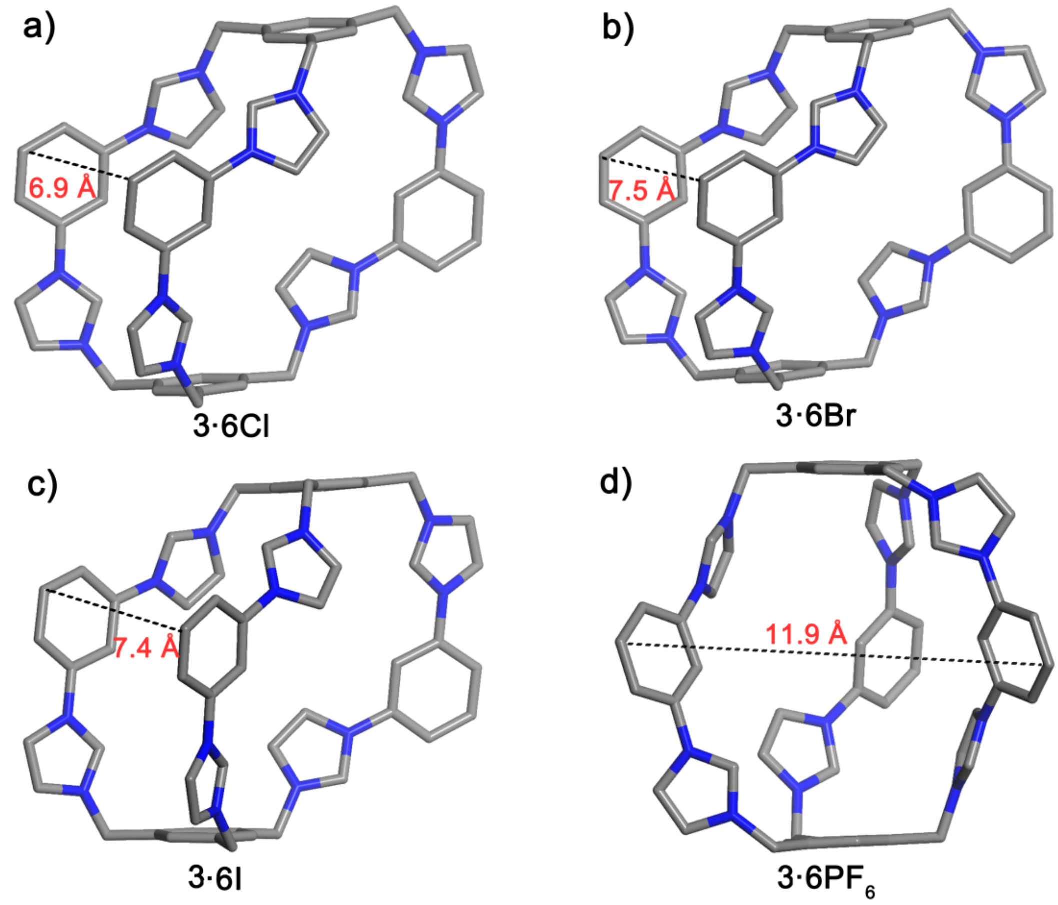

Supplementary Fig. 30 Crystal structures with distance indicated between the two closest diimidazolium units for (a) **3**·6Cl, (b) **3**·6Br, (c) **3**·6I, and (d) **3**·6PF_6, respectively. Counteranions and hydrogens are omitted for clarity.

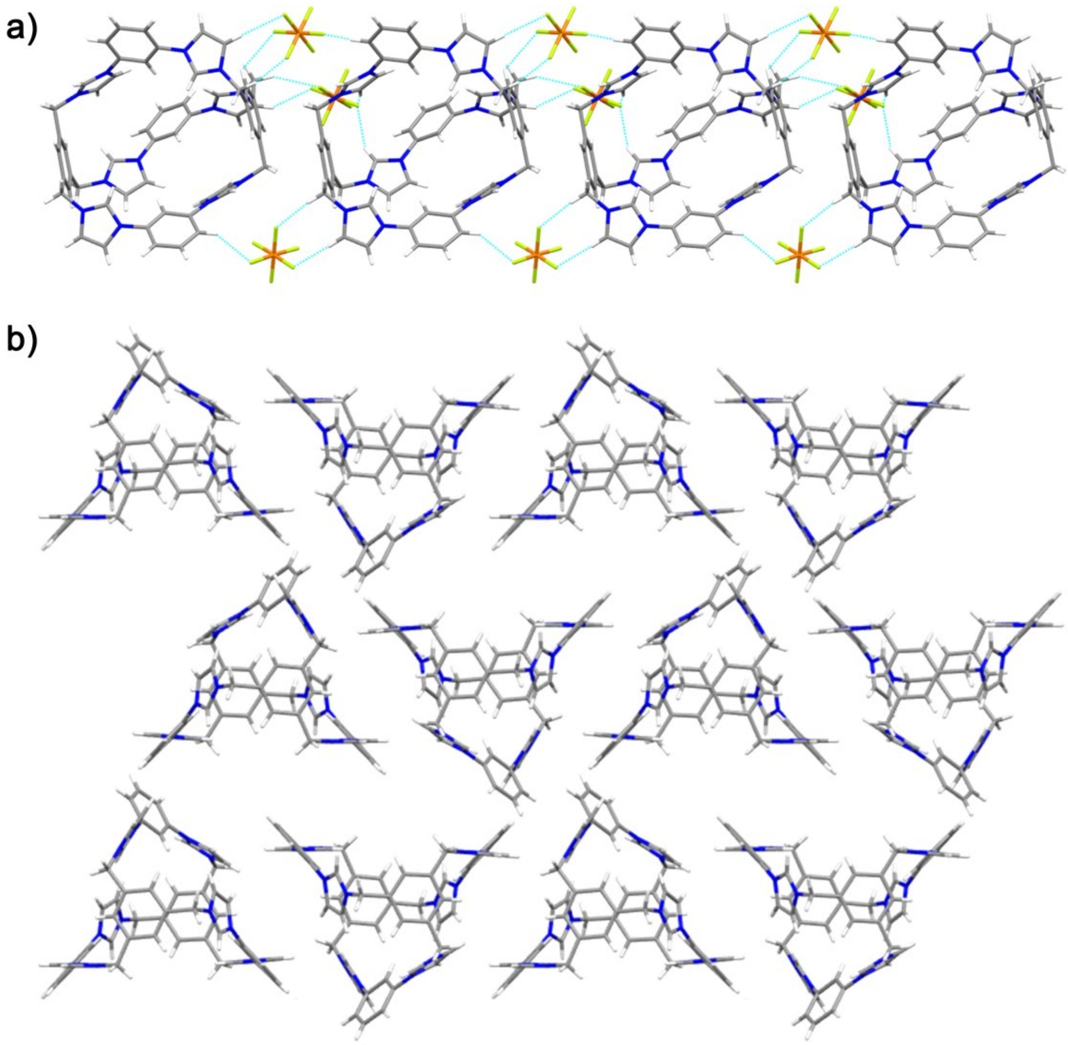

Supplementary Fig. 31 Crystal structure of **3**·$6PF_6$. (a) Side view of 1D chain connected by C-H···F hydrogen bonds. (b) Stacked 3D supramolecular structure. Solvent molecules and unrelated anions are omitted for clarity.

2.3 Supplementary PXRD spectra

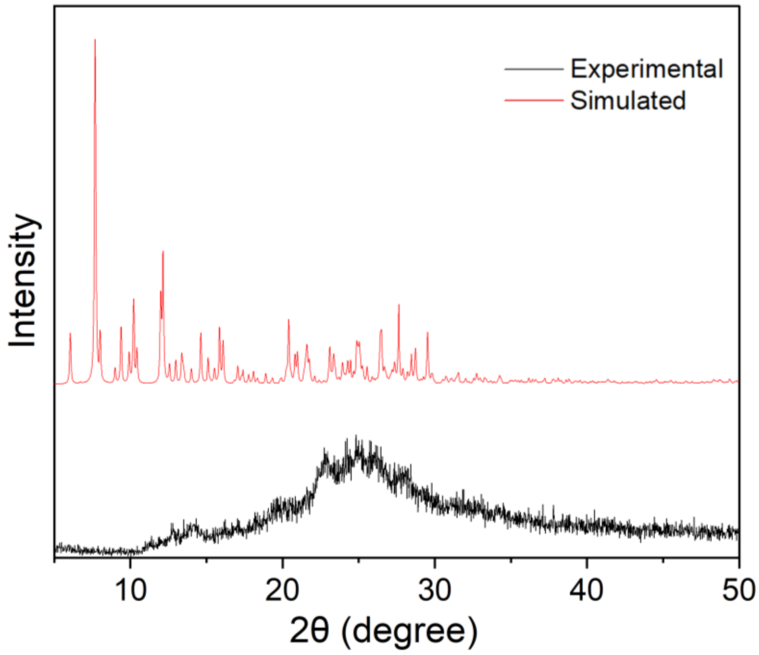

Supplementary Fig. 32 Powder X-ray diffraction pattern of **3**·6Cl.

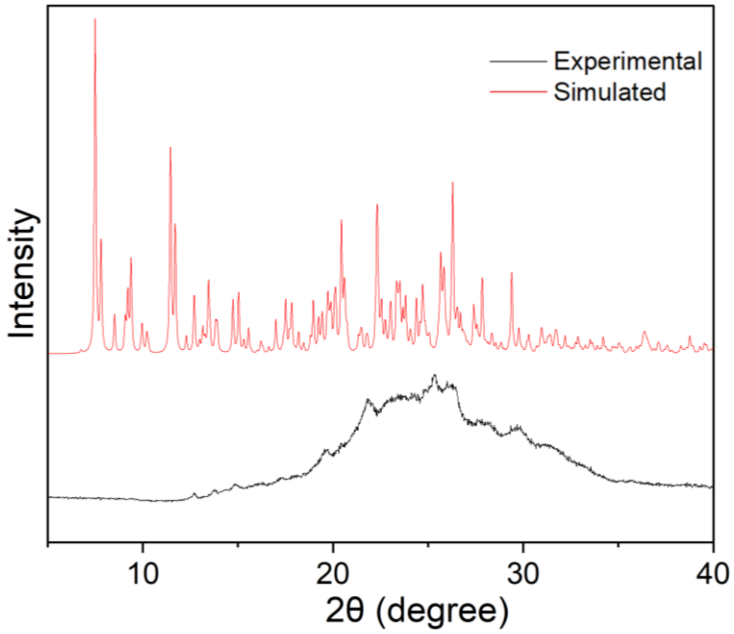

Supplementary Fig. 33 Powder X-ray diffraction pattern of **3**·6Br.

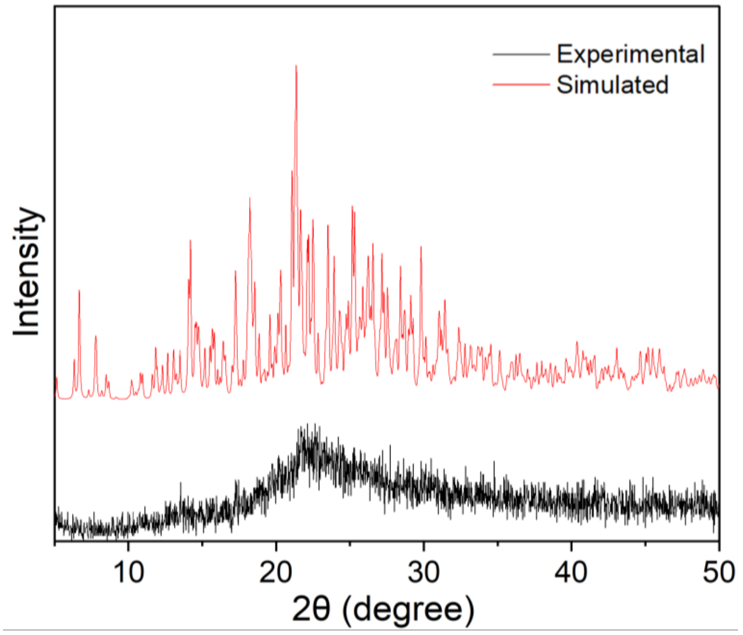

Supplementary Fig. 34 Powder X-ray diffraction pattern of **3**·6I.

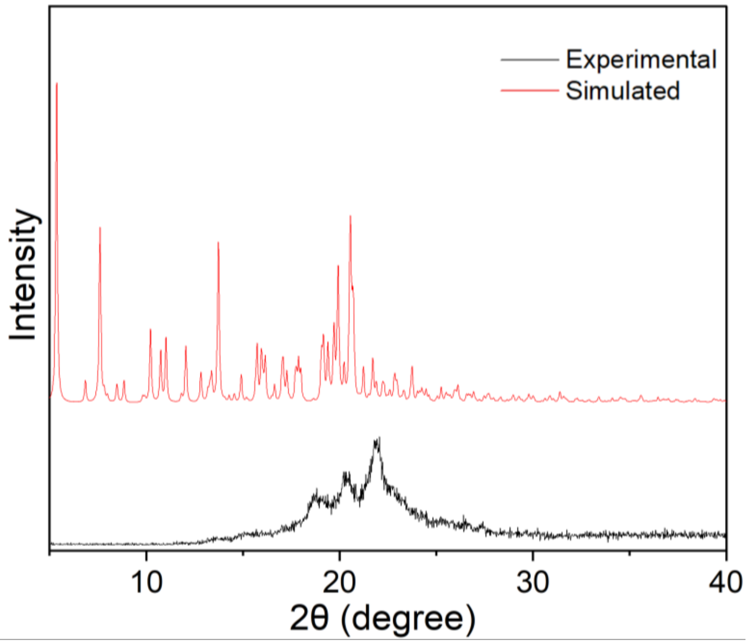

Supplementary Fig. 35 Powder X-ray diffraction pattern of **3**·$6PF_6$.

2.4 Supplementary N_2 adsorption isotherm

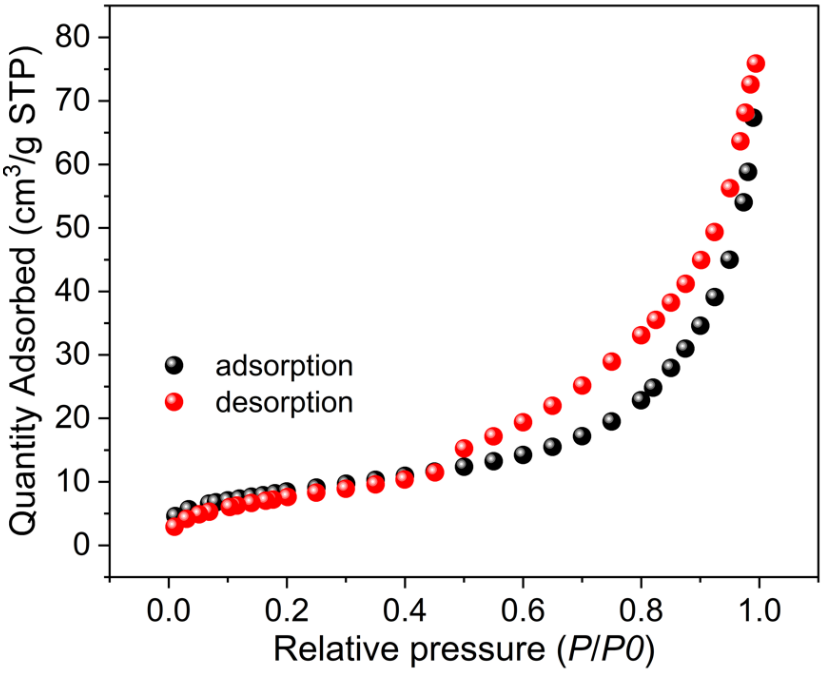

Supplementary Fig. 36 N_2 adsorption isotherm of **3**·6Cl.

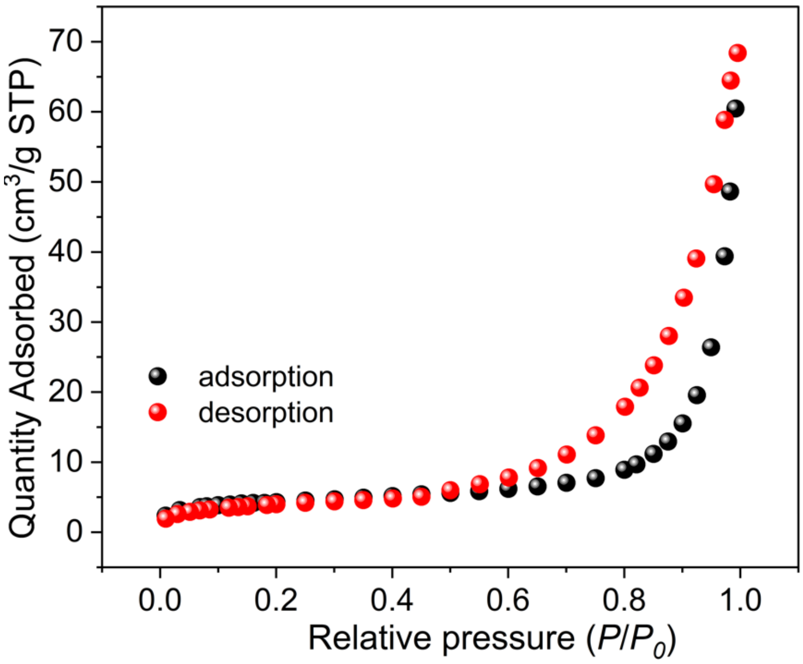

Supplementary Fig. 37 N_2 adsorption isotherm of **3**·6Br.

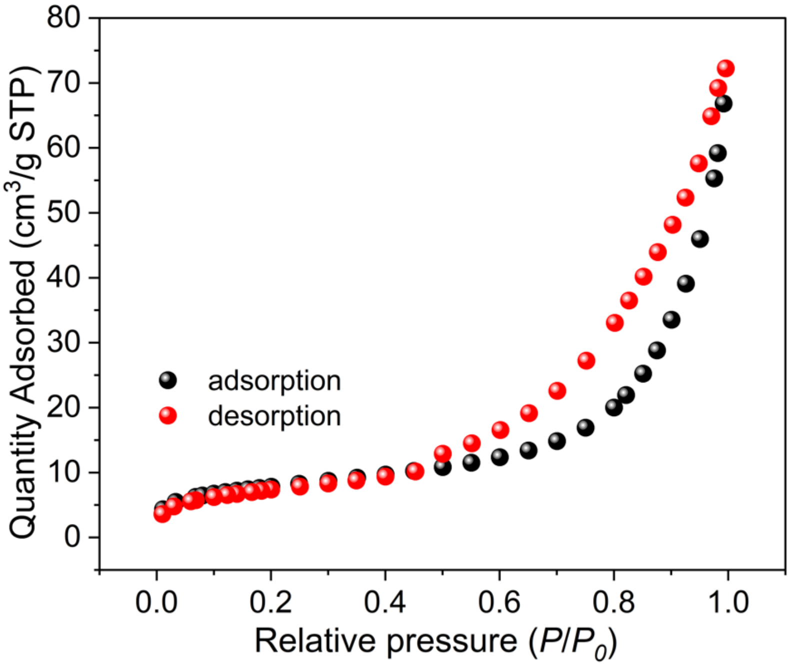

Supplementary Fig. 38 N_2 adsorption isotherm of **3**·6I.

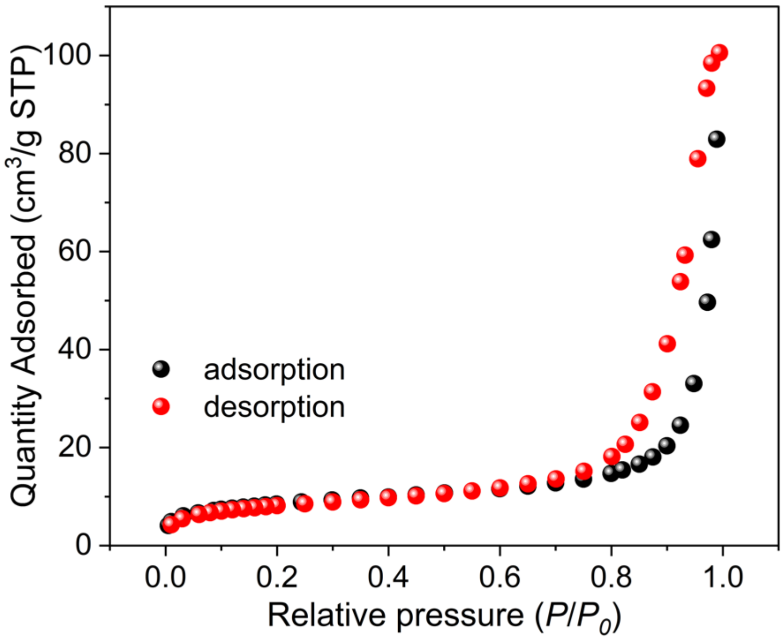

Supplementary Fig. 39 N_2 adsorption isotherm of **3**·6PF_6.

2.5 Supplementary TGA spectra

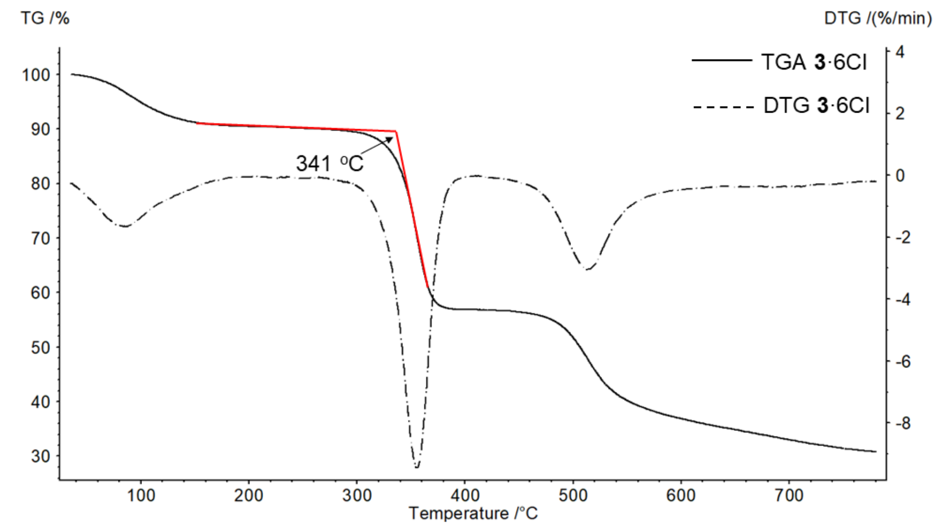

Supplementary Fig. 40 TGA and DTG spectra of **3**·6Cl, with framework decomposition temperature indicated.

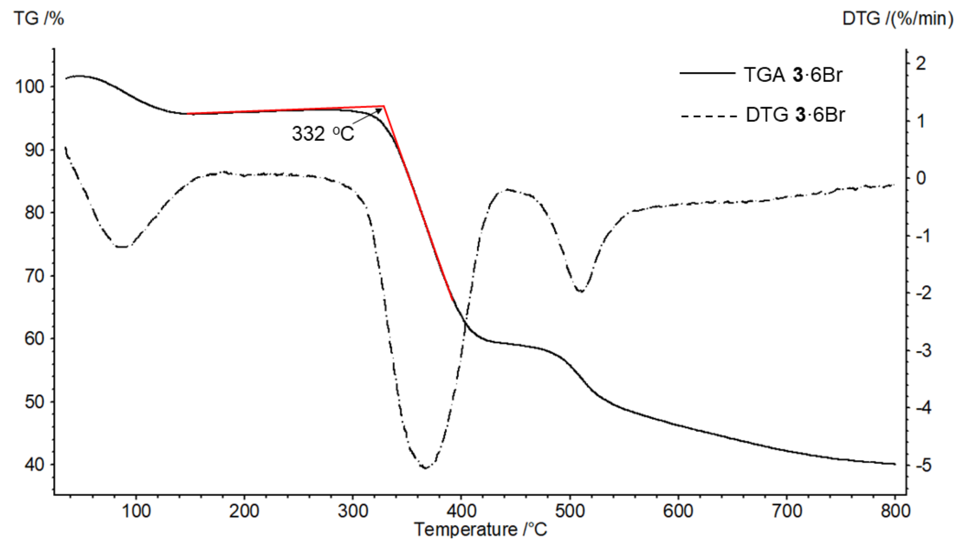

Supplementary Fig. 41 TGA and DTG spectra of **3**·6Br, with framework decomposition temperature indicated.

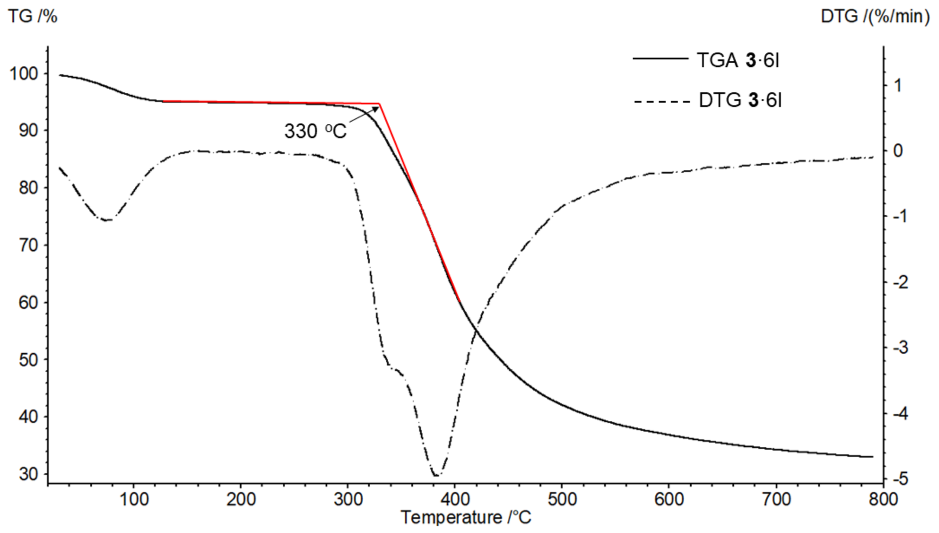

Supplementary Fig. 42 TGA and DTG spectra of **3**·6I, with framework decomposition temperature indicated.

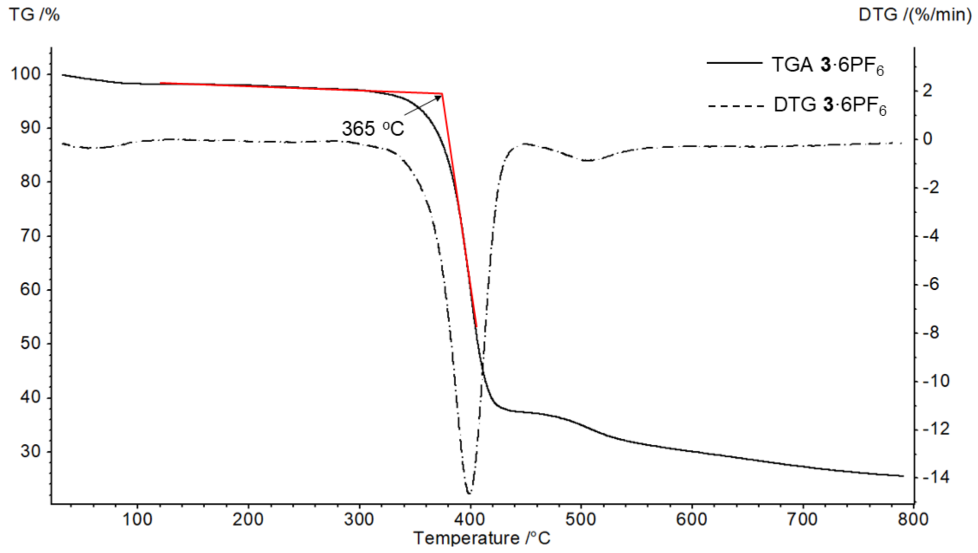

Supplementary Fig. 43 TGA and DTG spectra of **3**·6PF_6, with framework decomposition temperature indicated.

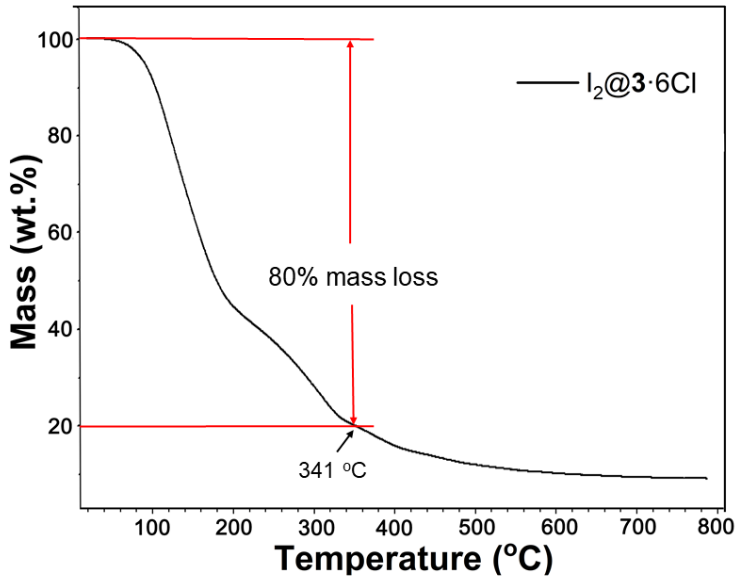

Supplementary Fig. 44 TGA spectrum of I_2@**3**·6Cl.

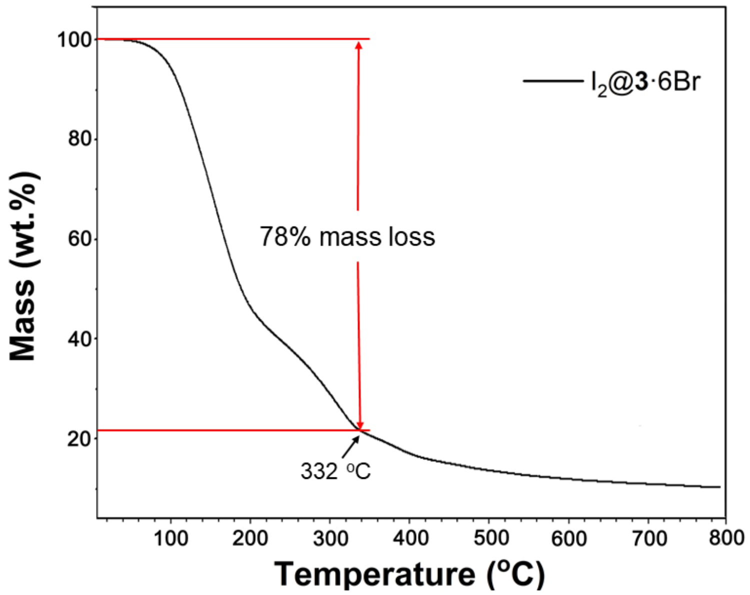

Supplementary Fig. 45 TGA spectrum of I_2@**3**·6Br.

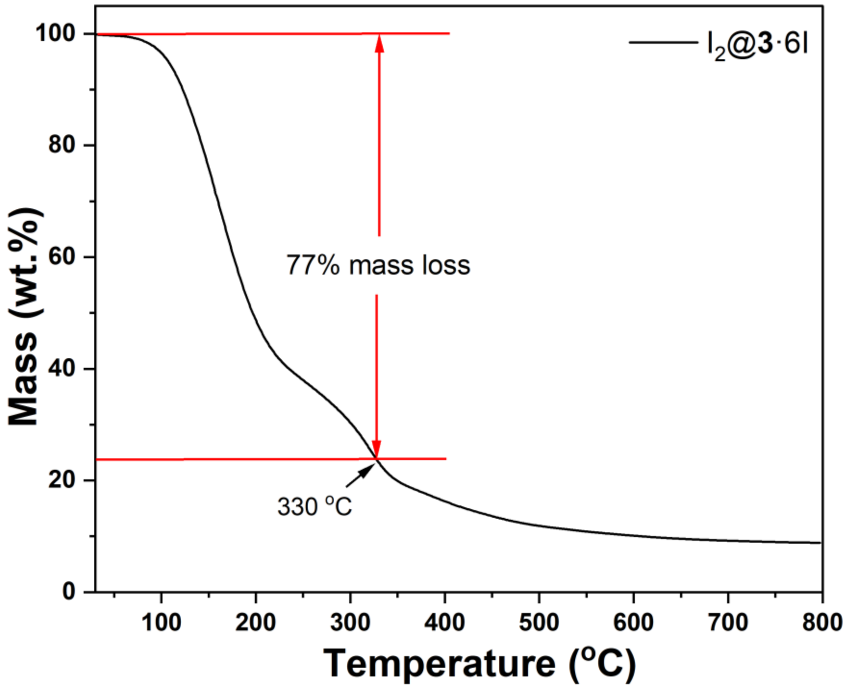

Supplementary Fig. 46 TGA spectrum of I_2@**3**·6I.

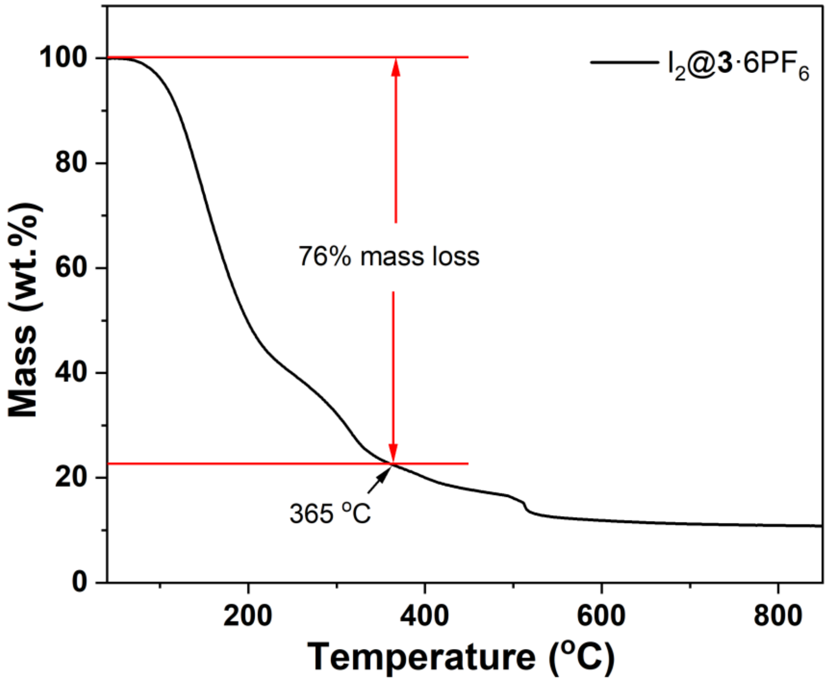

Supplementary Fig. 47 TGA spectrum of I_2@**3**·6PF_6.

2.6 Supplementary UV/vis absorption spectra

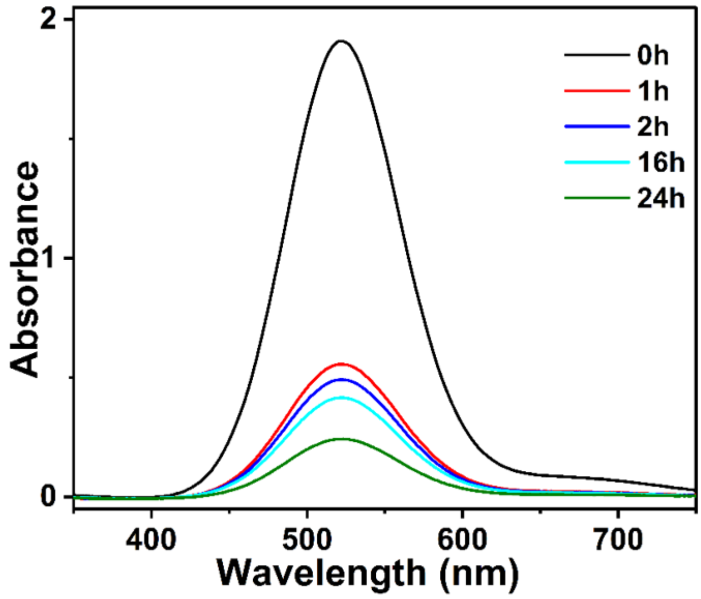

Supplementary Fig. 48 Time-dependent UV/vis absorption spectra of a solution of I_2 in n-hexane (2 mM, 3 mL) upon addition of 5 mg **3**·6Cl.

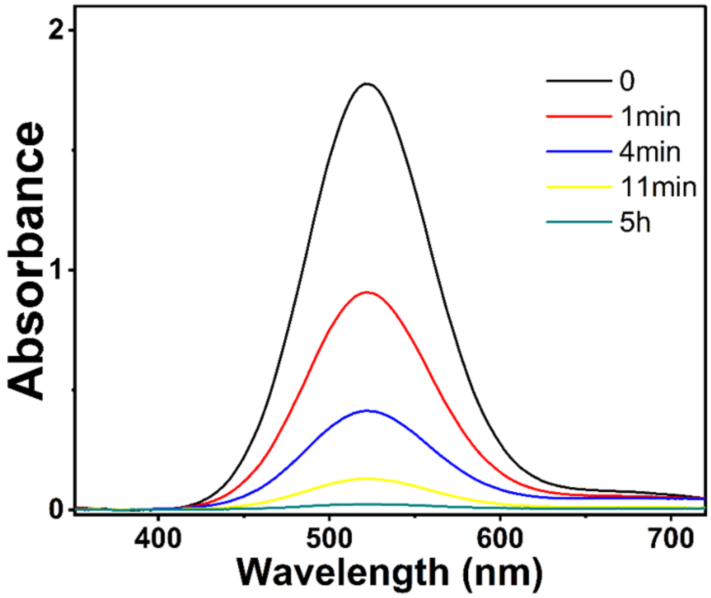

Supplementary Fig. 49 Time-dependent UV/vis absorption spectra of a solution of I_2 in n-hexane (2 mM, 3 mL) upon addition of 5 mg **3**·6Br.

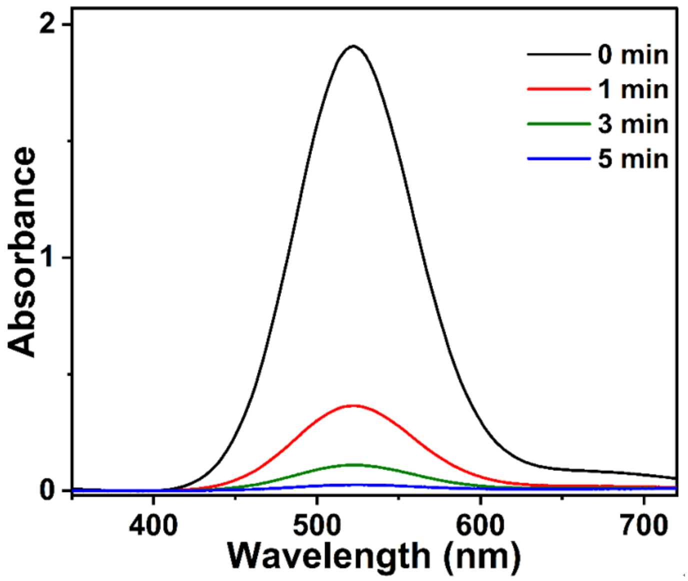

Supplementary Fig. 50 Time-dependent UV/vis absorption spectra of a solution of I_2 in n-hexane (2 mM, 3 mL) upon addition of 5 mg **3**·6I.

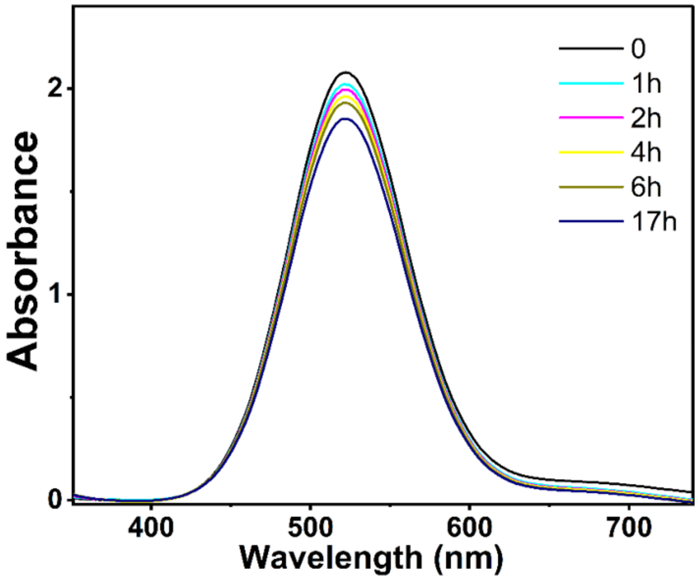

Supplementary Fig. 51 Time-dependent UV/vis absorption spectra of a solution of I_2 in n-hexane (2 mM, 3 mL) upon addition of 5 mg **3**·6PF_6.

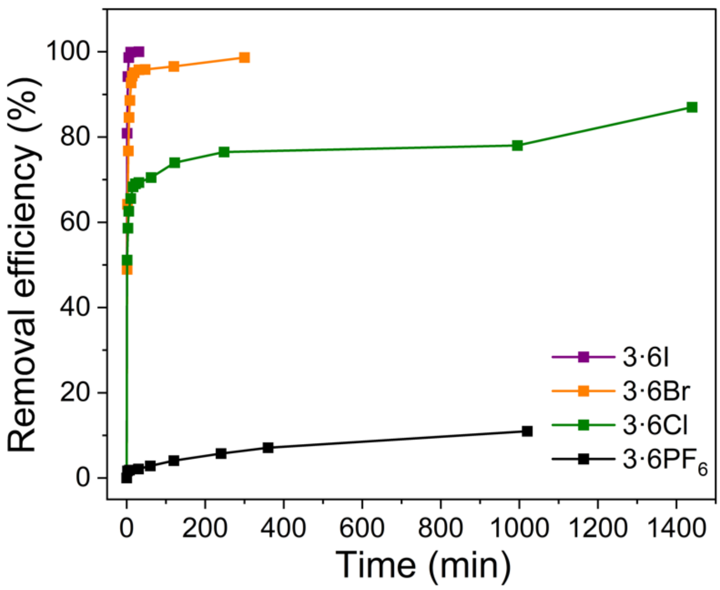

Supplementary Fig. 52 Iodine removal efficiency in n-hexane (2 mM, 3 mL) at various times by 5 mg **3**·6X (X = Cl^-, Br^-, I^-, PF_6^-), monitored by change of I_2 absorbance at 522 nm.

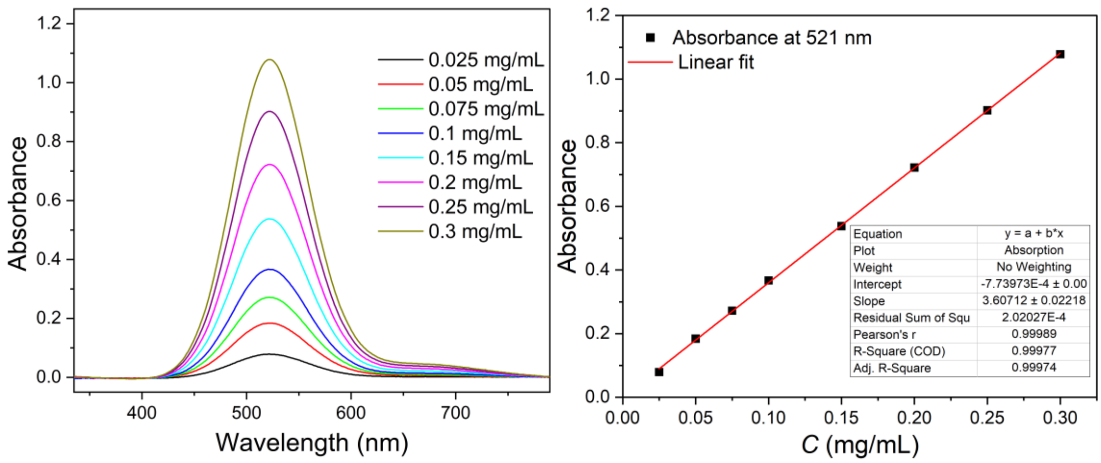

Supplementary Fig. 53 Standard calibration plot of iodine in n-hexane solution determined by UV-vis spectra.

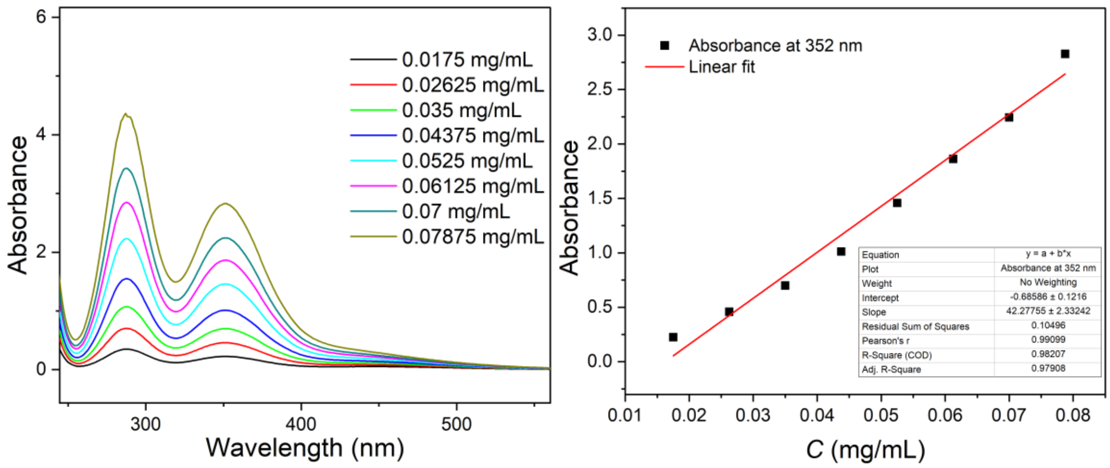

Supplementary Fig. 54 Standard calibration plot of aqueous solution of I_2/KI by UV-vis spectra.

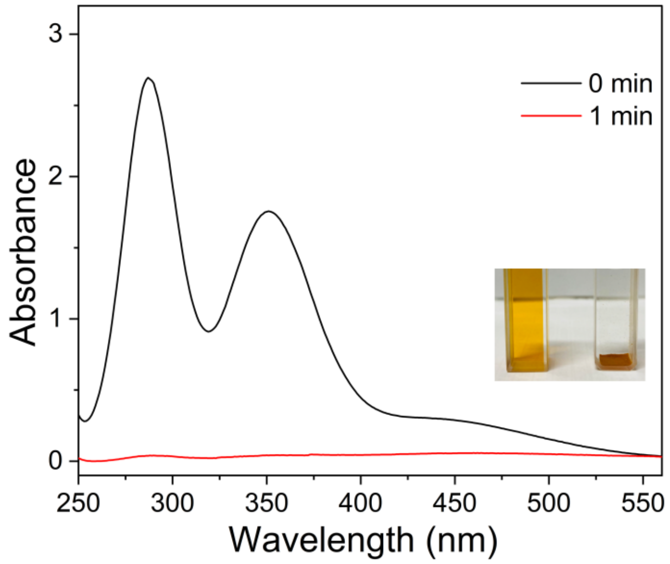

Supplementary Fig. 55 Time-dependent UV/vis absorption spectra of an aqueous solution of I_2/KI (0.4 mM, 3 mL) upon addition of 5 mg **3**·$6PF_6$. Inset: photographs of an aqueous solution of I_2/KI before and after addition of **3**·$6PF_6$.

2.7 Supplementary FT-IR spectra

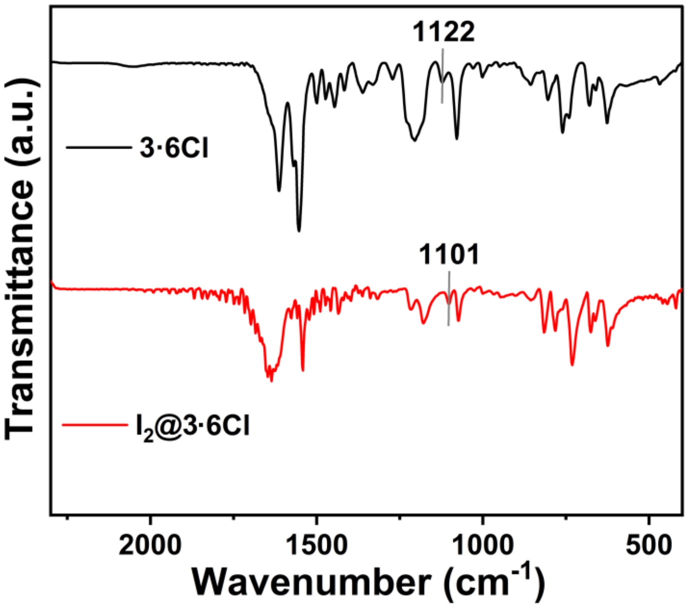

Supplementary Fig. 56 FT-IR spectra of **3**·6Cl before and after I_2 adsorption.

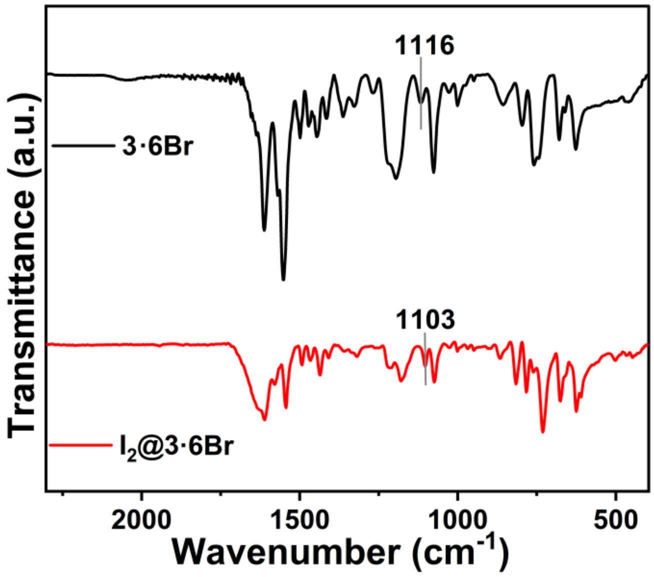

Supplementary Fig. 57 FT-IR spectra of **3**·6Br before and after I_2 adsorption.

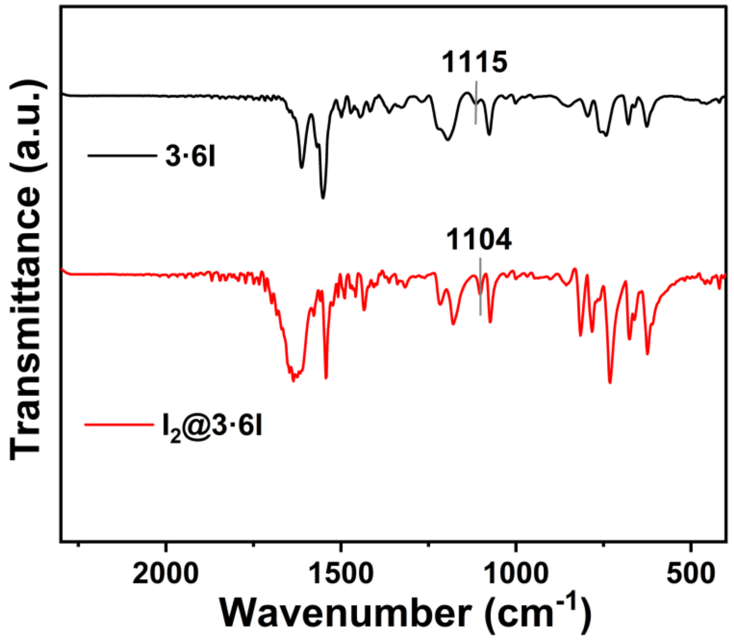

Supplementary Fig. 58 FT-IR spectra of **3**·6I before and after I_2 adsorption.

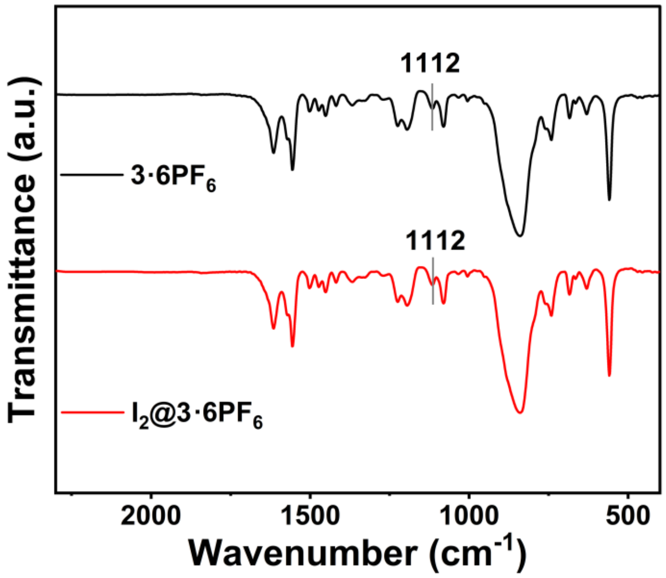

Supplementary Fig. 59 FT-IR spectra of **3**·6PF_6 before and after I_2 adsorption.

2.8 Supplementary Raman spectra

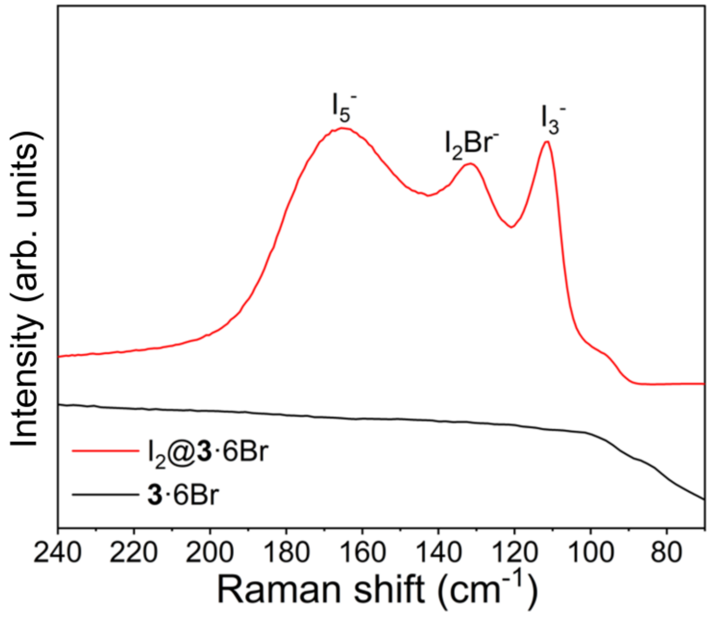

Supplementary Fig. 60 Raman spectra of **3**·6Br before and after I_2 adsorption.

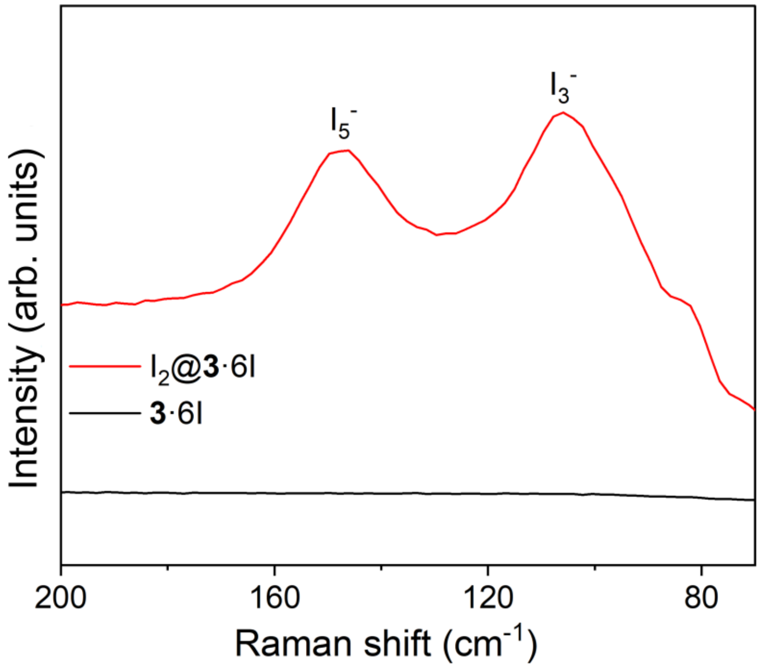

Supplementary Fig. 61 Raman spectra of **3**·6I before and after I_2 adsorption.

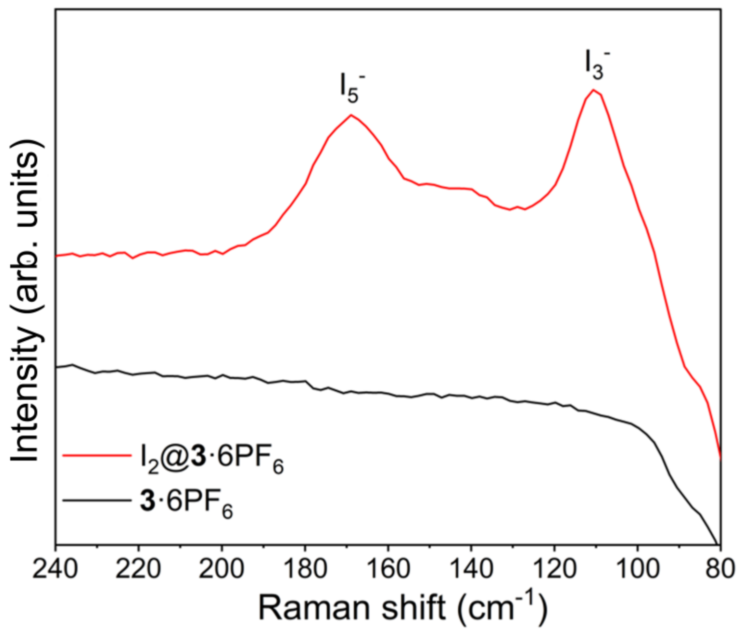

Supplementary Fig. 62 Raman spectra of **3**·6PF_6 before and after I_2 adsorption.

2.9 Supplementary XPS spectra

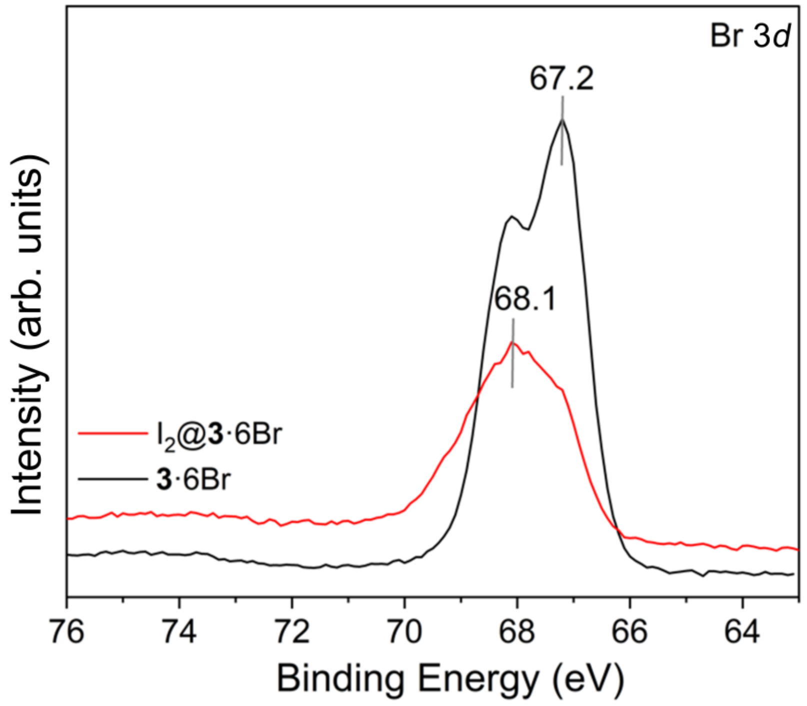

Supplementary Fig. 63 Br 3*d* XPS spectra of **3**·6Br before and after I_2 adsorption.

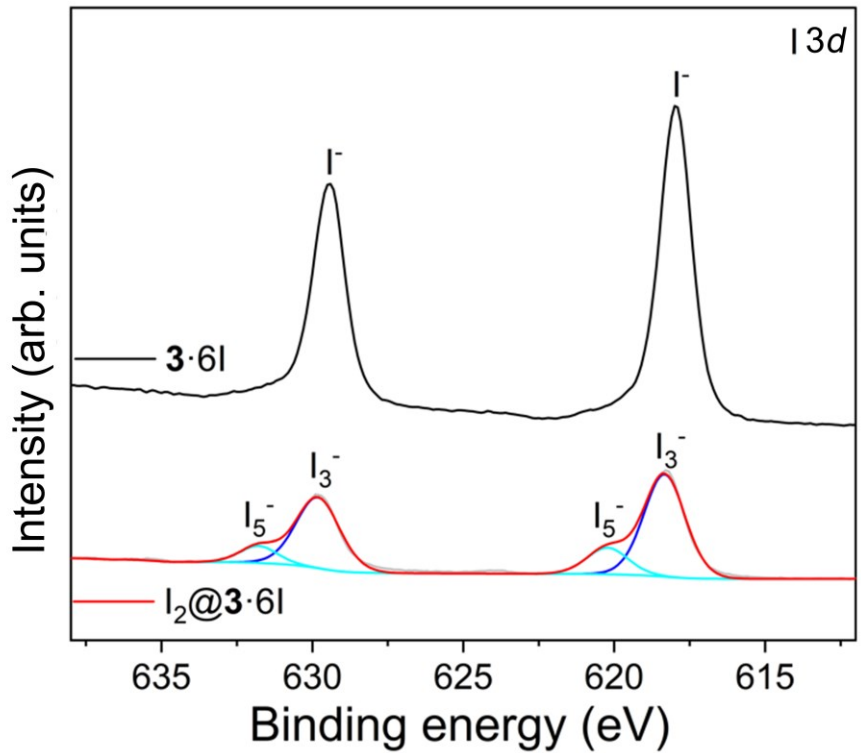

Supplementary Fig. 64 I 3*d* XPS spectra of **3**·6I before and after I_2 adsorption.

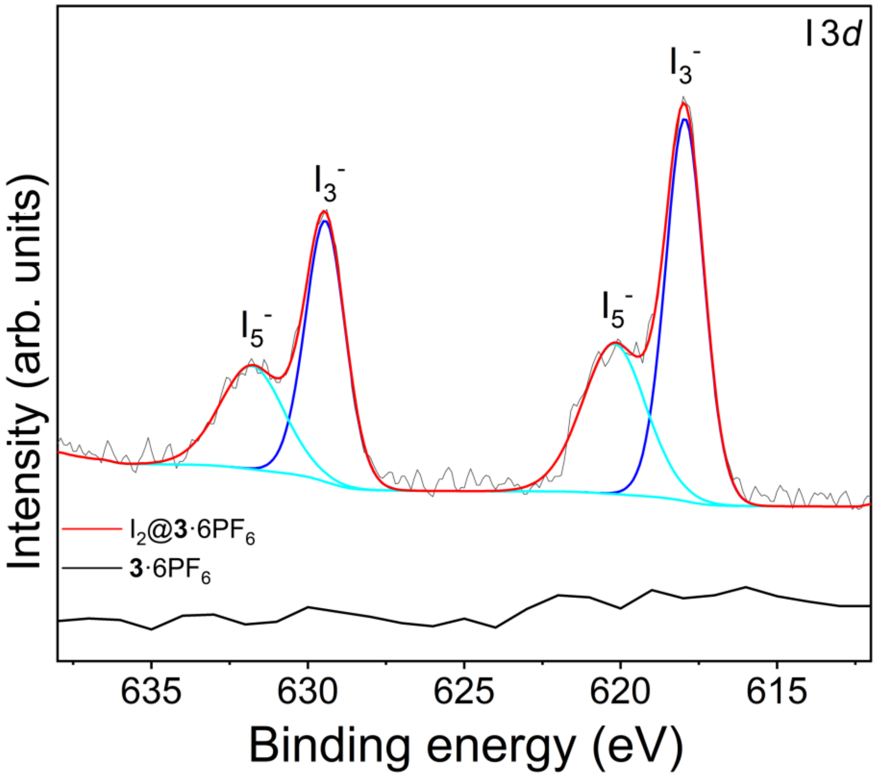

Supplementary Fig. 65 I 3*d* XPS spectra of **3**·$6PF_6$ before and after I_2 adsorption.

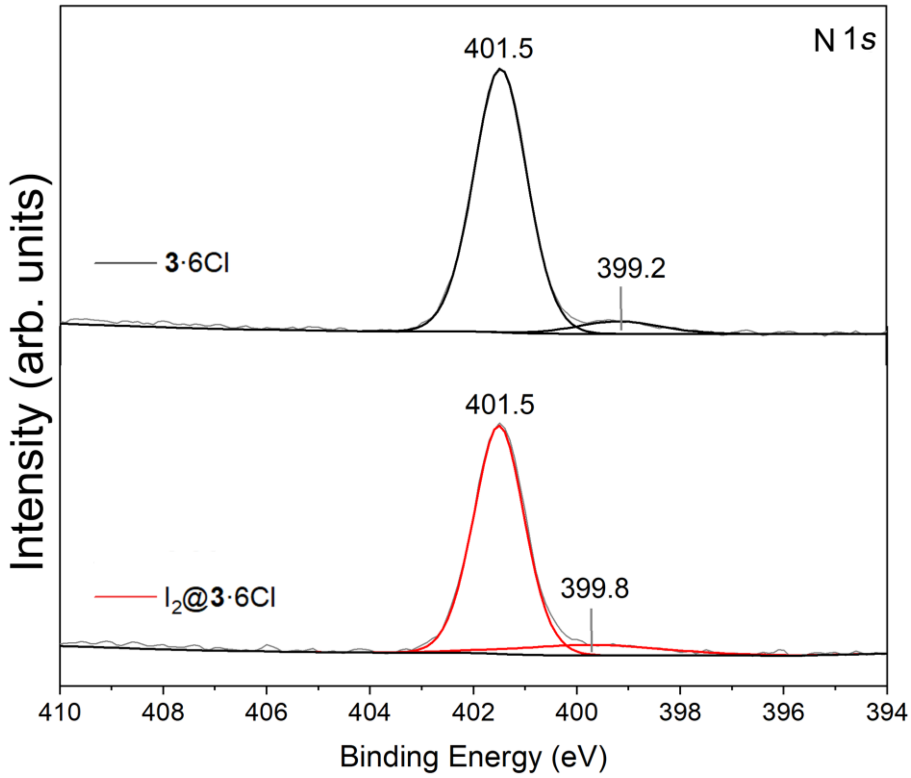

Supplementary Fig. 66 N 1*s* XPS spectra of **3**·6Cl before and after I_2 adsorption.

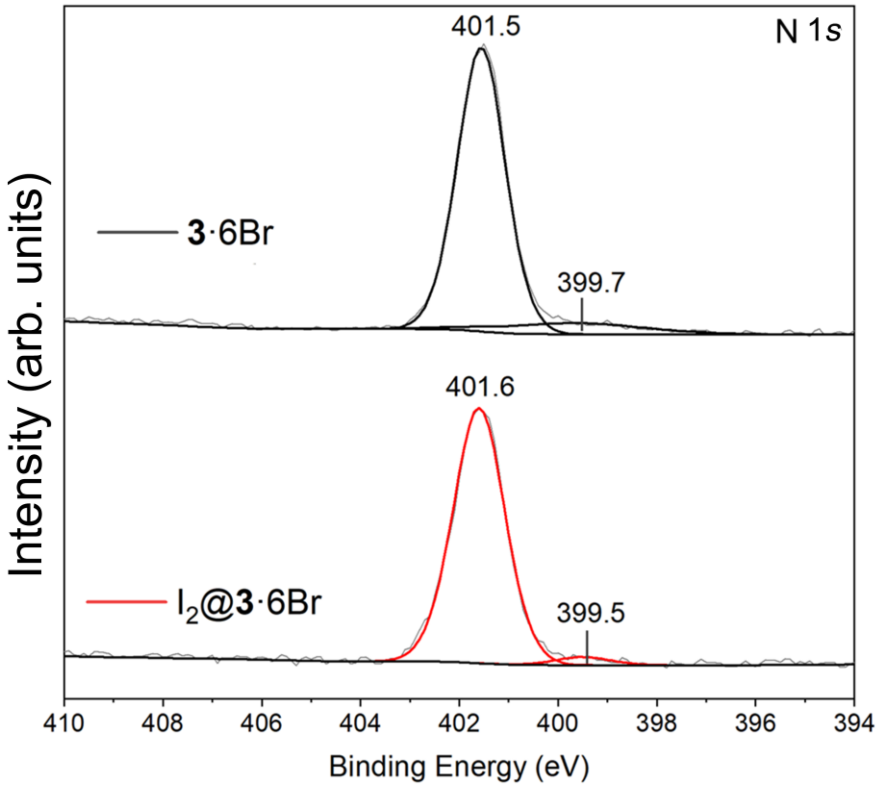

Supplementary Fig. 67 N 1*s* XPS spectra of **3**·6Br before and after I_2 adsorption.

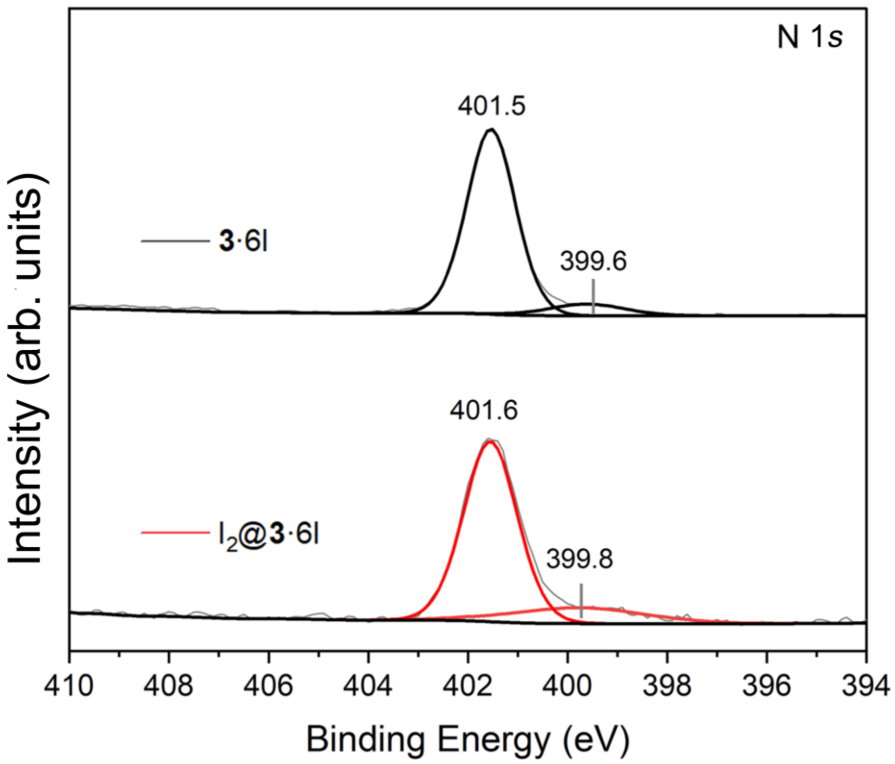

Supplementary Fig. 68 N 1*s* XPS spectra of **3**·6I before and after I_2 adsorption.

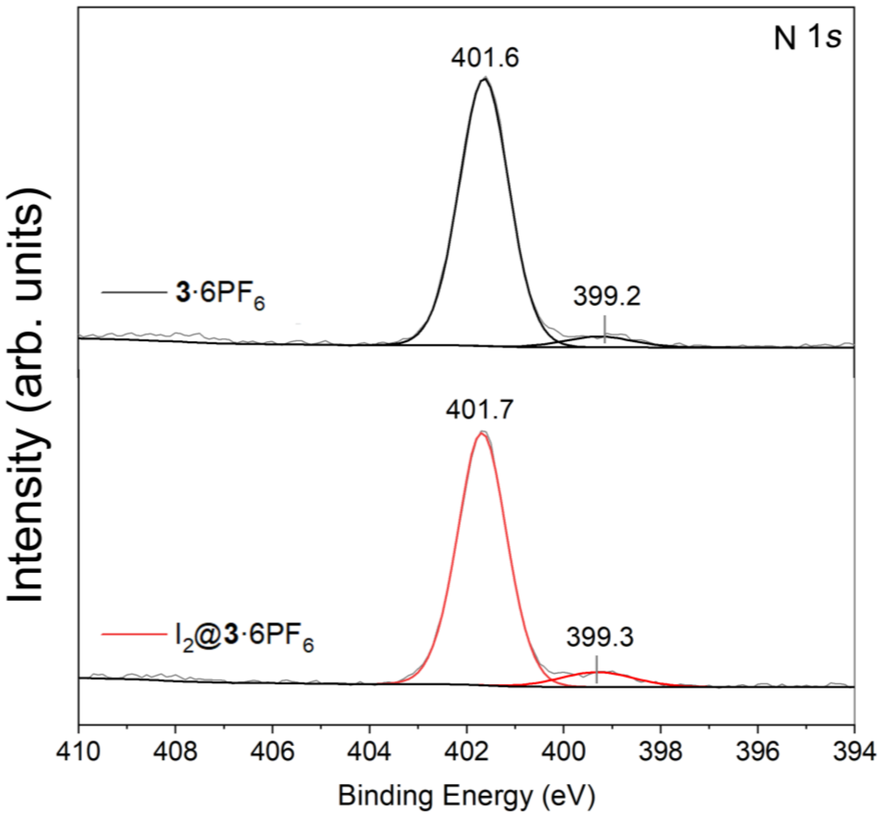

Supplementary Fig. 69 N 1*s* XPS spectra of **3**·6PF_6 before and after I_2 adsorption.

2.10 Supplementary ^{1}H NMR titration experiments spectra

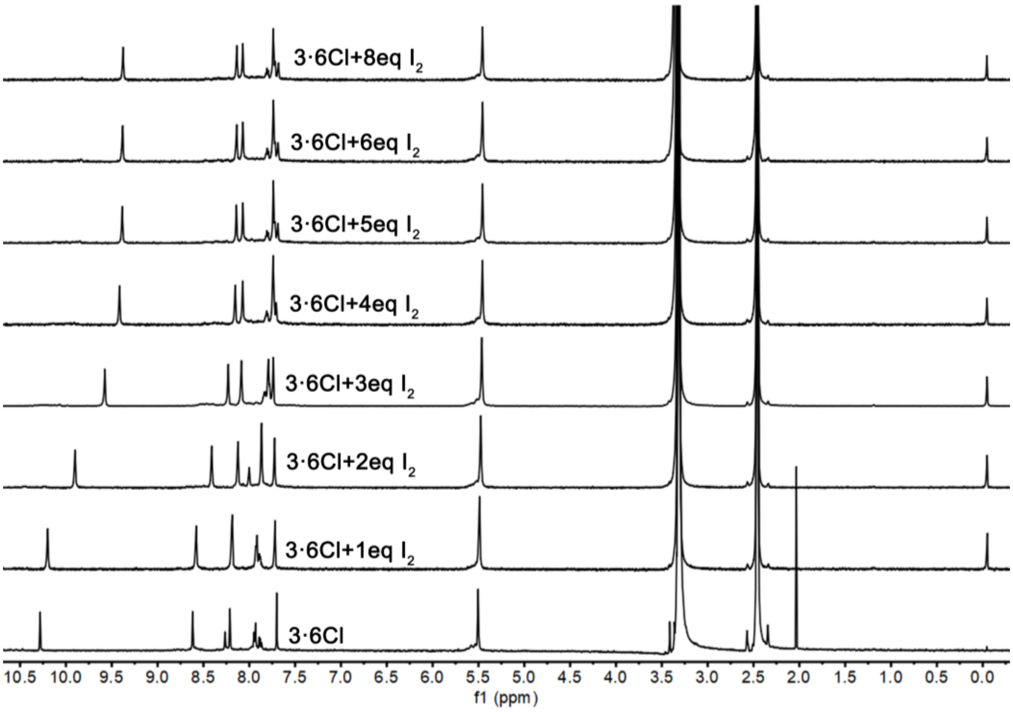

Supplementary Fig. 70 Stacked ^{1}H NMR spectra of **3**·6Cl upon addition of different equivalence of I_2 (600 MHz, DMSO-d_6, 298 K).

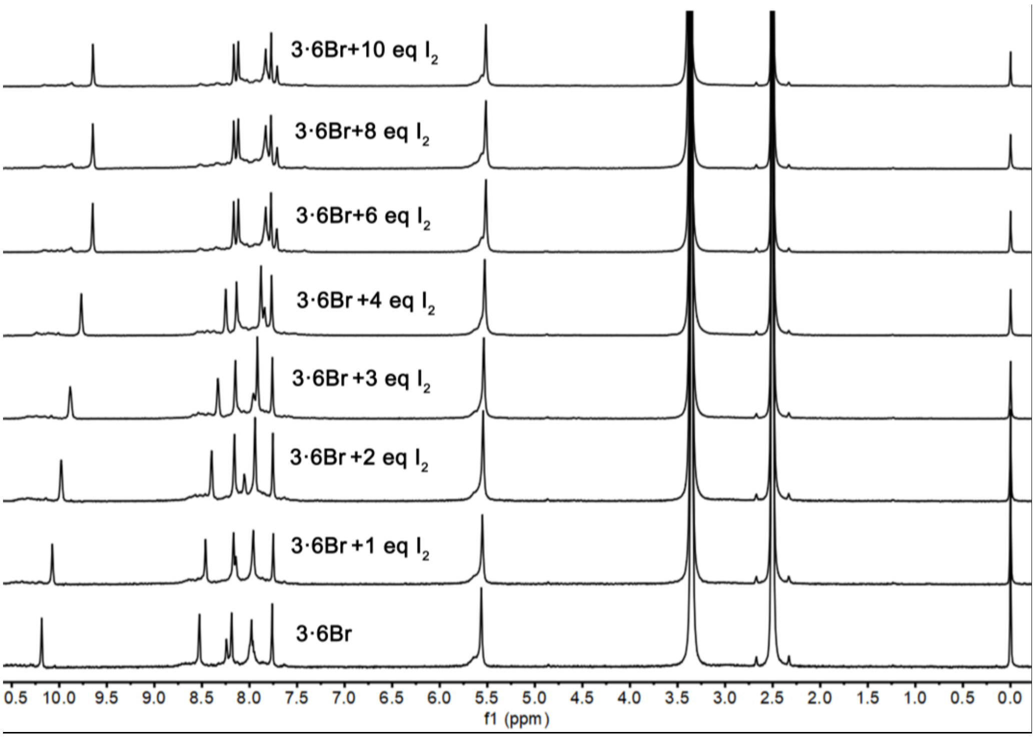

Supplementary Fig. 71 Stacked ^{1}H NMR spectra of **3**·6Br upon addition of different equivalence of I_2 (600 MHz, DMSO-d_6, 298 K).

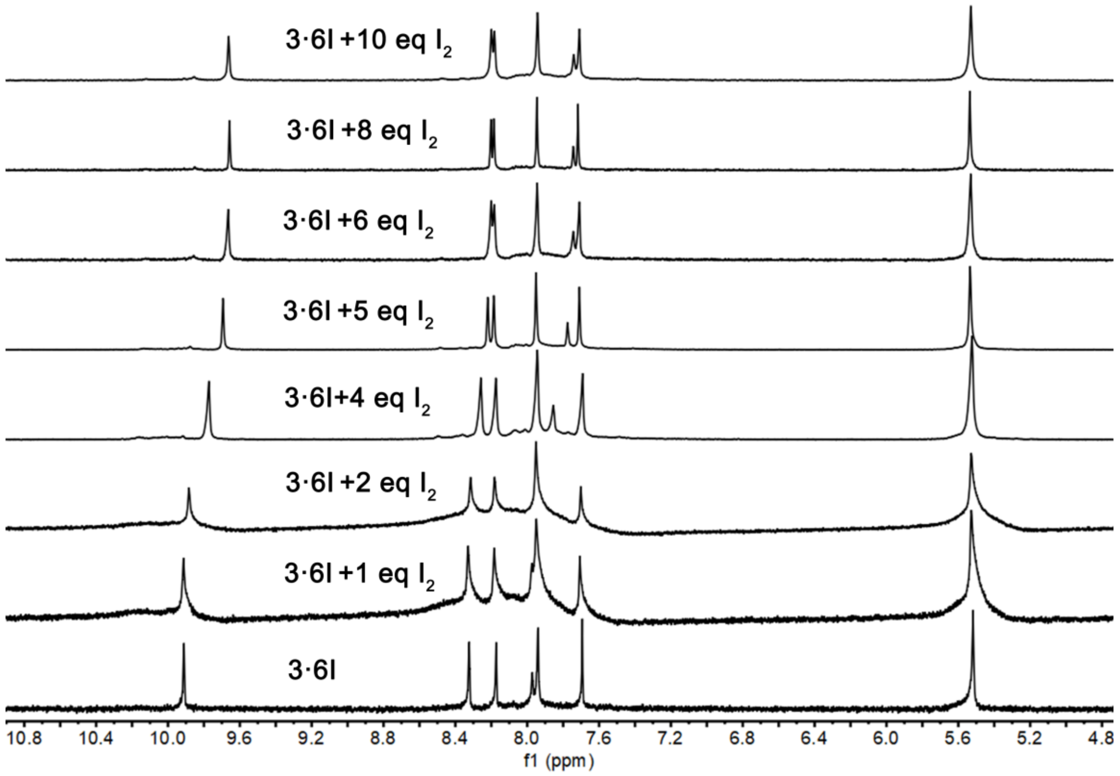

Supplementary Fig. 72 Stacked ^{1}H NMR spectra of **3**·6I upon addition of different equivalence of I_2 (600 MHz, DMSO-d_6, 298 K).

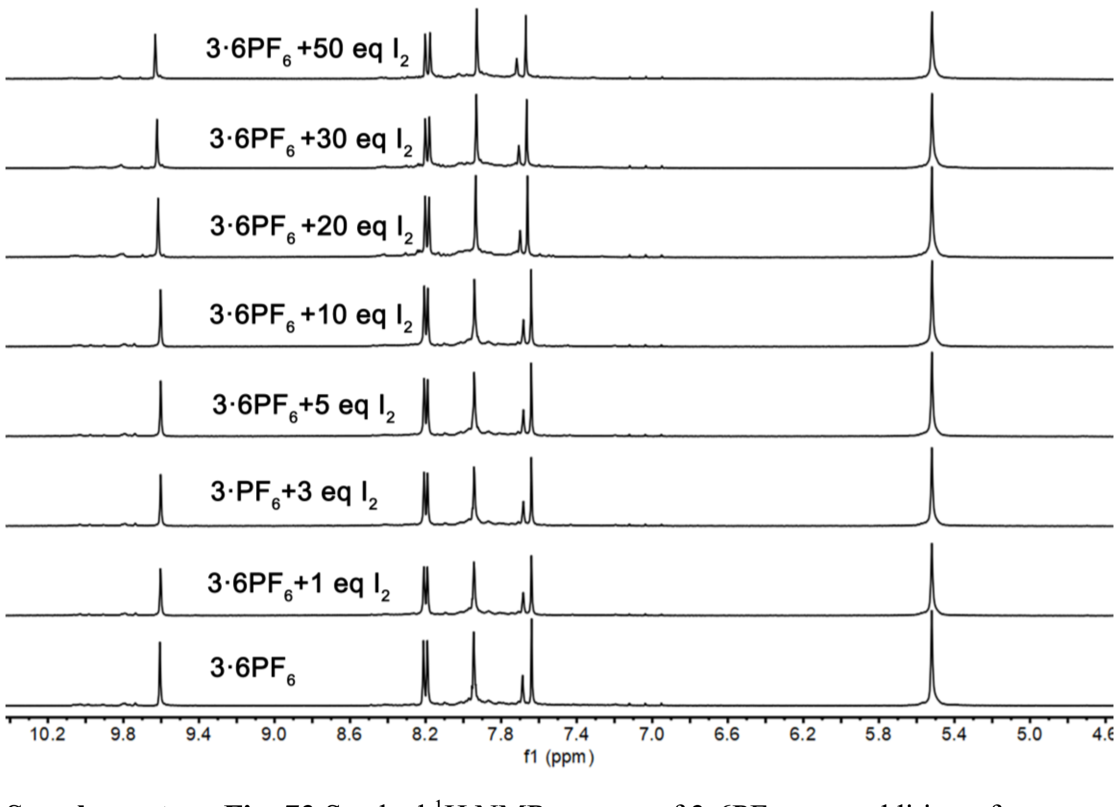

Supplementary Fig. 73 Stacked ^{1}H NMR spectra of **3**·6PF_6 upon addition of different equivalence of I_2 (600 MHz, DMSO-d_6, 298 K).

3. Supplementary tables

Supplementary Table 1. Summary of porosity information of different cages.

**Cage**	**3**·6Cl	**3**·6Br	**3**·6I	**3**·$6PF_6$
**BET Surface Area (m^2/g)**	30.77	15.29	27.60	29.93
**Adsorption average pore width by BET (nm)**	10.86	15.93	12.39	20.79

Supplementary Table 2. Crystal data and structure refinement for **3**·6Cl (CCDC 2234458).

Identification code	cageCl
Empirical formula	$C_{54}H_{48}Cl_6N_{12}$
Formula weight	1077.74
Temperature/K	100.15
Crystal system	monoclinic
Space group	$P2_1/c$
a/Å	13.6528(7)
b/Å	22.9332(10)
c/Å	19.5435(11)
α/°	90
β/°	106.068(6)
γ/°	90
Volume/Å^3	5880.1(5)
Z	4
ρ_{calc}g/cm^3	1.217
μ/mm^{-1}	1.997
F(000)	2232.0
Crystal size/mm^3	0.2 × 0.15 × 0.1
Radiation	GaKα (λ = 1.3405)
2Θ range for data collection/°	5.288 to 120.578
Index ranges	$-17 \leq h \leq 17$, $-26 \leq k \leq 29$, $-24 \leq l \leq 25$
Reflections collected	44011
Independent reflections	12967 [R_{int} = 0.0321, R_{sigma} = 0.0322]
Data/restraints/parameters	12967/0/678
Goodness-of-fit on F^2	1.015

Final R indexes [I>=2σ (I)]	R_1 = 0.0744, wR_2 = 0.1966
Final R indexes [all data]	R_1 = 0.0885, wR_2 = 0.2051
Largest diff. peak/hole / e Å^{-3}	0.66/-0.58

Supplementary Table 3. Crystal data and structure refinement for **3**·6Br (CCDC 2234459).

Identification code	cageBr
Empirical formula	$C_{54}H_{48}N_{12}Br_6O$
Formula weight	1344.50
Temperature/K	301(2)
Crystal system	monoclinic
Space group	C2/c
a/Å	38.547(8)
b/Å	13.939(2)
c/Å	23.671(5)
α/°	90
β/°	94.098(6)
γ/°	90
Volume/Å^3	12686(4)
Z	8
ρ_{calc}g/cm^3	1.408
μ/mm^{-1}	3.840
F(000)	5328.0
Crystal size/mm^3	0.15 × 0.1 × 0.08
Radiation	MoKα (λ = 0.71073)
2Θ range for data collection/°	4.564 to 42.118
Index ranges	$-38 \leq h \leq 38$, $-14 \leq k \leq 14$, $-23 \leq l \leq 23$
Reflections collected	47876
Independent reflections	6810 [R_{int} = 0.1196, R_{sigma} = 0.0931]
Data/restraints/parameters	6810/469/649
Goodness-of-fit on F^2	1.485
Final R indexes [I>=2σ (I)]	R_1 = 0.1298, wR_2 = 0.3763
Final R indexes [all data]	R_1 = 0.1833, wR_2 = 0.4232
Largest diff. peak/hole / e Å^{-3}	1.67/-2.11

Supplementary Table 4. Crystal data and structure refinement for **3**·6I (CCDC 2234460).

Identification code	cageI
Empirical formula	$C_{56}H_{56}I_6N_{12}O_2S$
Formula weight	1722.58
Temperature/K	150.00(10)
Crystal system	triclinic
Space group	P-1
a/Å	14.03844(17)
b/Å	21.1682(2)
c/Å	22.3833(2)
α/°	75.0123(10)
β/°	85.6441(9)
γ/°	86.4936(9)
Volume/Å^3	6400.84(13)
Z	4
ρ_{calc}g/cm^3	1.788
μ/mm^{-1}	16.171
F(000)	3304.0
Crystal size/mm^3	0.04 × 0.03 × 0.02
Radiation	GaKα (λ = 1.3405)
2Θ range for data collection/°	4.47 to 105.858
Index ranges	$-16 \leq h \leq 15$, $-25 \leq k \leq 25$, $-26 \leq l \leq 26$
Reflections collected	88423
Independent reflections	22605 [R_{int} = 0.0488, R_{sigma} = 0.0365]
Data/restraints/parameters	22605/6/1461
Goodness-of-fit on F^2	1.036
Final R indexes [I>=2σ (I)]	R_1 = 0.0592, wR_2 = 0.1795
Final R indexes [all data]	R_1 = 0.0661, wR_2 = 0.1844
Largest diff. peak/hole / e Å^{-3}	3.25/-5.75

Supplementary Table 5. Crystal data and structure refinement for **3**·6PF6 (CCDC 2234461).

Identification code	cagePF6
Empirical formula	$C_{54}H_{48}F_{36}N_{12}P_6$
Formula weight	1734.86
Temperature/K	293
Crystal system	monoclinic
Space group	$P2_1/n$
a/Å	13.9971(5)
b/Å	23.3167(9)
c/Å	23.5433(11)
α/°	90
β/°	100.018(5)
γ/°	90
Volume/Å^3	7566.6(6)
Z	4
ρ_{calc}g/cm^3	1.523
μ/mm^{-1}	1.638
F(000)	3480.0
Crystal size/mm^3	0.15 × 0.12 × 0.1
Radiation	GaKα (λ = 1.3405)
2Θ range for data collection/°	4.674 to 91.514
Index ranges	$-14 \leq h \leq 14$, $-24 \leq k \leq 24$, $-25 \leq l \leq 25$
Reflections collected	40482
Independent reflections	9583 [R_{int} = 0.0638, R_{sigma} = 0.0670]
Data/restraints/parameters	9583/1317/973
Goodness-of-fit on F^2	1.043
Final R indexes [I>=2σ (I)]	R_1 = 0.1010, wR_2 = 0.2711
Final R indexes [all data]	R_1 = 0.1329, wR_2 = 0.2909
Largest diff. peak/hole / e Å^{-3}	1.56/-0.83

Supplementary Table 6. Crystal data and structure refinement for **3**·$[I_2Br]_5[I_4Br]$ (CCDC 2234462).

Identification code	cageI4Br
Empirical formula	$C_{54}H_{48}N_{12}Br_6I_{14}$
Formula weight	3121.10
Temperature/K	100.0(2)
Crystal system	triclinic
Space group	P-1
a/Å	14.2268(2)
b/Å	14.4251(2)
c/Å	19.8823(3)
α/°	92.9620(10)
β/°	95.2350(10)
γ/°	94.2910(10)
Volume/Å^3	4044.86(10)
Z	2
ρ_{calc}g/cm^3	2.563
μ/mm^{-1}	31.450
F(000)	2816.0
Crystal size/mm^3	0.25 × 0.15 × 0.1
Radiation	GaKα (λ = 1.3405)
2Θ range for data collection/°	3.886 to 120.142
Index ranges	$-14 \le h \le 18$, $-18 \le k \le 18$, $-25 \le l \le 24$
Reflections collected	56157
Independent reflections	17945 [R_{int} = 0.0434, R_{sigma} = 0.0342]
Data/restraints/parameters	17945/0/746
Goodness-of-fit on F^2	1.045
Final R indexes [I>=2σ (I)]	R_1 = 0.0803, wR_2 = 0.2580
Final R indexes [all data]	R_1 = 0.0855, wR_2 = 0.2639
Largest diff. peak/hole / e Å^{-3}	10.24/-5.08

Supplementary Table 7. Comparison for iodine uptake capacities of different adsorbents.

Adsorbent	Type	S_{BET} (m^2•g^{-1})	T (°C)	Time (h)	Capacity (g g^{-1})	Ref.
BisImi-POP@2	POP	-	77	48	10.30	8
iCOF-AB-50	COF	1390	75	30	10.21	9
TBIM	POP	8.12	77	72	9.43	10
IL@PCN-333(Al)	MOF	1635.3	75	12	7.35	11
SR-KOH	PC	3072	77	1	6.46	12
QTD-COF-V	COF	-	75	4	6.29	13
SCU-COF-2	COF	413	75	96	6.00	14
**CageCl**	**POC**	**30.77**	**75**	**24**	**5.89**	**This work**
BPPOC	POC	577	75	50	5.64	15
TPT-BD COF	COF	109	75	32	5.43	16
PCN-333(Al)	MOF	2935.9	75	12	4.42	11
OMC3	POC	80	75	12	3.78	17
Bpy-cage	POC	1.8	75	14	3.23	18
BTPOC	POC	605	75	14	3.21	19
OM-COF-300	COF	1410	75	36	3.15	20
HcOF-1	HCOF	-	75	-	2.90	21
PAF-24	POP	136	75	48	2.76	22
CalP4	POP	759	75	24	2.20	23
cage-1	MOC	137	75	36	1.42	24
AlOC-26-NC	MC	508	80	2.5	0.70	25
CC3	POC	-	20	350	0.45	26
EtP6β	MC	-	85	2	0.20	27

Notes: POP: porous organic polymer, COF: covalent organic framework, MOF: metal organic framework, PC: porous carbon, POC: porous organic cage, HCOF: hydrogen-bonded cross-linked organic framework, MC: molecular crystal, MOC: metal organic cage.

4. Supplementary references

1. Walter, S. M.; Kniep, F.; Herdtweck, E.; Huber, S. M. *Angew. Chemie Int. Ed.* **2011**, *50*, 7187–7191.

2. Liu, Y.-J.; Sun, Y.; Shen, S.; Wang, S.; Liu, Z.; Fang, W.; Wright, D. S.; Zhang, J. *Nat. Commun.* **2022**, *13*, 6632.

3. Gogia, A.; Das, P.; Mandal, S. K. *ACS Appl. Mater. Interfaces* **2020**, *12*, 46107–46118.

4. Agilent Technologies, CrysAlisPro v. 1.171.36.28, **2013**.

5. APEX III, Data collection software (version 2017.3)

6. Sheldrick, G. M. Acta Crystallogr. Sect. A, **2008**, *64*, 112.

7. Spek, A. L. Single-Crystal Structure Validation with the Program PLATON. *J. Appl. Crystallogr*. **2003**, *36*, 7.

8. Niu, T.-H.; Feng, C.-C.; Yao, C.; Yang, W.-Y.; Xu, Y.-H. *ACS Appl. Polym. Mater.* **2021**, *3*, 354–361.

9. Xie, Y.; Pan, T.; Lei, Q.; Chen, C.; Dong, X.; Yuan, Y.; Shen, J.; Cai, Y.; Zhou, C.; Pinnau, I.; Han, Y. *Angew. Chemie Int. Ed.* **2021**, *60*, 22432–22440.

10. Geng, T.; Zhang, C.; Liu, M.; Hu, C.; Chen, G. *J. Mater. Chem. A* **2020**, *8*, 2820–2826.

11. Tang, Y.; Huang, H.; Li, J.; Xue, W.; Zhong, C. *J. Mater. Chem. A* **2019**, *7*, 18324–18329.

12. Sun, H.; Yang, B.; Li, A. *Chem. Eng. J.* **2019**, *372*, 65–73.

13. Guo, X.; Li, Y.; Zhang, M.; Cao, K.; Tian, Y.; Qi, Y.; Li, S.; Li, K.; Yu, X.; Ma, L. *Angew. Chemie - Int. Ed.* **2020**, *59*, 22697–22705.

14. He, L.; Chen, L.; Dong, X.; Zhang, S.; Zhang, M.; Dai, X.; Liu, X.; Lin, P.; Li, K.; Chen, C.; Pan, T.; Ma, F.; Chen, J.; Yuan, M.; Zhang, Y.; Chen, L.; Zhou, R.; Han, Y.; Chai, Z.; Wang, S. A *Chem* **2021**, *7*, 699–714.

15. Liu, C.; Jin, Y.; Yu, Z.; Gong, L.; Wang, H.; Yu, B.; Zhang, W.; Jiang, J. *J. Am. Chem. Soc.* **2022**, *144*, 12390–12399.

16. Guo, X.; Tian, Y.; Zhang, M.; Li, Y.; Wen, R.; Li, X.; Li, X.; Xue, Y.; Ma, L.; Xia, C.; Li, S. *Chem. Mater.* **2018**, *30*, 2299–2308.

17. Zhang, L.; Jin, Y.; Tao, G. H.; Gong, Y.; Hu, Y.; He, L.; Zhang, W. *Angew. Chemie - Int. Ed.* **2020**, *59*, 20846–20851.

18. Luo, D.; He, Y.; Tian, J.; Sessler, J. L.; Chi, X. *J. Am. Chem. Soc.* **2022**, *144*, 113–117.

19. Liu, C.; Li, W.; Liu, Y.; Wang, H.; Yu, B.; Bao, Z.; Jiang, J. *Chem. Eng. J.* **2022**, *428*, 131129.

20. Liu, T.; Zhao, Y.; Song, M.; Pang, X.; Shi, X.; Jia, J.; Chi, L.; Lu, G. *J. Am. Chem. Soc.* **2023**, *145*, 2544−2552.

21. Lin, Y.; Jiang, X.; Kim, S. T.; Alahakoon, S. B.; Hou, X.; Zhang, Z.; Thompson, C. M.; Smaldone, R. A.; Ke, C. *J. Am. Chem. Soc.* **2017**, *139*, 7172–7175.

22. Yan, Z.; Yuan, Y.; Tian, Y.; Zhang, D.; Zhu, G. *Angew. Chemie Int. Ed.* **2015**, *54*, 12733–12737.

23. Shetty, D.; Raya, J.; Han, D. S.; Asfari, Z.; Olsen, J. C.; Trabolsi, A. *Chem. Mater.* **2017**, *29*, 8968–8972.

24. Pei, W. Y.; Yang, J.; Wu, H.; Zhou, W.; Yang, Y. W.; Ma, J. F. *Chem. Commun.* **2020**, *56*, 2491–2494.

25. Yao, S.; Fang, W.-H.; Sun, Y.; Wang, S.-T.; Zhang, J. *J. Am. Chem. Soc.* **2021**, *143*, 2325–2330.

26. Hasell, T.; Schmidtmann, M.; Cooper, A. I. *J. Am. Chem. Soc*. **2011**, *133*, 14920-14923.

27. Jie, K.; Zhou, Y.; Li, E.; Li, Z.; Zhao, R.; Huang, F. *J. Am. Chem. Soc.* **2017**, *139*, 15320–15323.
