# Supplementary material for: Counteranion-mediated efficient iodine capture in a hexacationic imidazolium organic cage enabled by multiple non-covalent interactions
Source: Nat Commun. 2023 Sep 28;14:6082. doi: 10.1038/s41467-023-41866-7 (PMC10539326; doi:10.1038/s41467-023-41866-7)
Supplement: Supplementary file 3 — Description of Additional Supplementary Files [file 41467_2023_41866_MOESM3_ESM.pdf]

Description of Additional Supplementary Files

File Name: Supplementary Data 1

Description: Crystallographic data and checkcif data
