# Supplementary material for: Counteranion-mediated efficient iodine capture in a hexacationic imidazolium organic cage enabled by multiple non-covalent interactions
Source: Nat Commun. 2023 Sep 28;14:6082. doi: 10.1038/s41467-023-41866-7 (PMC10539326; doi:10.1038/s41467-023-41866-7)

checkCIF/PLATON report

Structure factors have been supplied for datablock(s) cageBr

THIS REPORT IS FOR GUIDANCE ONLY. IF USED AS PART OF A REVIEW PROCEDURE FOR PUBLICATION, IT SHOULD NOT REPLACE THE EXPERTISE OF AN EXPERIENCED CRYSTALLOGRAPHIC REFEREE.

No syntax errors found. CIF dictionary Interpreting this report

Datablock: cageBr

Bond precision: C-C = 0.0256 A Wavelength=0.71073

Cell:	a=38.547(8)	b=13.939(2)	c=23.671(5)
	alpha=90	beta=94.098(6)	gamma=90
Temperature:	301 K		

	Calculated	Reported
Volume	12686(4)	12686(4)
Space group	C 2/c	C 2/c
Hall group	-C 2yc	-C 2yc
Moiety formula	C54 H48 N12, 6(Br) [+ solvent]	?
Sum formula	C54 H48 Br6 N12 [+ solvent]	C54 H48 Br6 N12 O0
Mr	1344.44	1344.50
Dx,g cm-3	1.408	1.408
Z	8	8
Mu (mm-1)	3.840	3.840
F000	5328.0	5328.0
F000’	5316.21	
h,k,lmax	38,14,23	38,14,23
Nref	6864	6810
Tmin,Tmax	0.637,0.736	
Tmin’	0.557	

Correction method= Not given

Data completeness= 0.992 Theta(max)= 21.059

R(reflections)= 0.1298(3916) wR2(reflections)= 0.4232(6810)

S = 1.485 Npar= 649

The following ALERTS were generated. Each ALERT has the format
test-name_ALERT_alert-type_alert-level.
Click on the hyperlinks for more details of the test.

Alert level A

```
THETM01_ALERT_3_A  The value of sine(theta_max)/wavelength is less than 0.550
            Calculated sin(theta_max)/wavelength =    0.5056
```

Author Response: This is due to weak diffraction of the crystal, and the data at the high angle is poor.

Alert level B

```
PLAT084_ALERT_3_B High wR2 Value (i.e. > 0.25) ...................       0.42 Report
```

Author Response: This is due to weak diffraction of the crystal.

```
PLAT260_ALERT_2_B Large Average Ueq of Residue Including       Br3      0.426 Check
```

Author Response: These alerts are caused by ghost peaks around heavy atoms.

```
PLAT260_ALERT_2_B Large Average Ueq of Residue Including       Br5      0.343 Check
```

Author Response: These alerts are caused by ghost peaks around heavy atoms.

```
PLAT260_ALERT_2_B Large Average Ueq of Residue Including       Br6      0.322 Check
```

Author Response: These alerts are caused by ghost peaks around heavy atoms.

```
PLAT341_ALERT_3_B Low Bond Precision on  C-C Bonds ..............    0.02562 Ang.
```

Author Response: These alerts result from the high level of thermal motion (or minor unresolved disorder) throughout the structure and the use of extensive thermal parameter restraints.

Alert level C

```
PLAT052_ALERT_1_C Info on Absorption Correction Method   Not Given     Please Do !
PLAT057_ALERT_3_C Correction for Absorption Required   RT(exp) ...       1.15 Do !
PLAT082_ALERT_2_C High R1 Value ................................       0.13 Report
PLAT250_ALERT_2_C Large U3/U1 Ratio for Average U(i,j) Tensor ....      2.2 Note
PLAT260_ALERT_2_C Large Average Ueq of Residue Including       Br2      0.147 Check
```

Author Response: These alerts are caused by ghost peaks around heavy atoms.

```
PLAT260_ALERT_2_C Large Average Ueq of Residue Including       Br4      0.124 Check
```

Author Response: These alerts are caused by ghost peaks around heavy atoms.

```
PLAT334_ALERT_2_C Small <C-C> Benzene Dist.   C37_2    -C42_2    .       1.37 Ang.
PLAT767_ALERT_4_C INS Embedded LIST 6 Instruction Should be LIST 4     Please Check
PLAT910_ALERT_3_C Missing # of FCF Reflection(s) Below Theta(Min).          9 Note
PLAT911_ALERT_3_C Missing FCF Refl Between Thmin & STh/L=    0.506         45 Report
PLAT918_ALERT_3_C Reflection(s) with I(obs) much Smaller I(calc) .          2 Check
PLAT934_ALERT_3_C Number of (Iobs-Icalc)/Sigma(W) > 10 Outliers ..          1 Check
PLAT971_ALERT_2_C Check Calcd Resid. Dens.  0.56Ang From Br3             1.58 eA-3
PLAT972_ALERT_2_C Check Calcd Resid. Dens.  0.59Ang From Br3            -2.05 eA-3
```

Alert level G

```
CELLZ01_ALERT_1_G Difference between formula and atom_site contents detected.
CELLZ01_ALERT_1_G ALERT: Large difference may be due to a
            symmetry error - see SYMMG tests
           From the CIF: _cell_formula_units_Z    8
           From the CIF: _chemical_formula_sum  C54 H48 Br6 N12 O0
           TEST: Compare cell contents of formula and atom_site data

           atom    Z*formula  cif sites diff
           C        432.00    432.00    0.00
           H        384.00    384.00    0.00
           Br        48.00     48.00    0.00
           N         96.00     96.00    0.00
           O          8.00      0.00    8.00
PLAT002_ALERT_2_G Number of Distance or Angle Restraints on AtSite          1 Note
PLAT003_ALERT_2_G Number of Uiso or Uij Restrained non-H Atoms ...         66 Report
PLAT172_ALERT_4_G The CIF-Embedded .res File Contains DFIX Records          1 Report
PLAT178_ALERT_4_G The CIF-Embedded .res File Contains SIMU Records          1 Report
PLAT188_ALERT_3_G A Non-default SIMU Restraint Value has been used     0.0100 Report
PLAT335_ALERT_2_G Check Large C6 Ring C-C Range C25_2    -C30_2         0.19 Ang.
PLAT432_ALERT_2_G Short Inter X...Y Contact  Br1      ..C62_2    .       3.31 Ang.
                                                     x,y,z  =      1_555 Check
PLAT434_ALERT_2_G Short Inter HL..HL Contact Br3      ..Br3      .       3.36 Ang.
                                               1-x,y,1/2-z  =      2_655 Check
PLAT606_ALERT_4_G Solvent Accessible VOID(S) in Structure ........          ! Info
PLAT720_ALERT_4_G Number of Unusual/Non-Standard Labels ..........        114 Note
PLAT860_ALERT_3_G Number of Least-Squares Restraints .............        469 Note
PLAT869_ALERT_4_G ALERTS Related to the Use of SQUEEZE  Suppressed          ! Info
PLAT883_ALERT_1_G No Info/Value for _atom_sites_solution_primary .     Please Do !
PLAT933_ALERT_2_G Number of HKL-OMIT Records in Embedded .res File         13 Note
PLAT978_ALERT_2_G Number C-C Bonds with Positive Residual Density.          1 Info
```

```
   1 ALERT level A = Most likely a serious problem - resolve or explain
   5 ALERT level B = A potentially serious problem, consider carefully
  14 ALERT level C = Check. Ensure it is not caused by an omission or oversight
  17 ALERT level G = General information/check it is not something unexpected

   4 ALERT type 1 CIF construction/syntax error, inconsistent or missing data
```

```
 17 ALERT type 2 Indicator that the structure model may be wrong or deficient
 10 ALERT type 3 Indicator that the structure quality may be low
  6 ALERT type 4 Improvement, methodology, query or suggestion
  0 ALERT type 5 Informative message, check
```

It is advisable to attempt to resolve as many as possible of the alerts in all categories. Often the minor alerts point to easily fixed oversights, errors and omissions in your CIF or refinement strategy, so attention to these fine details can be worthwhile. In order to resolve some of the more serious problems it may be necessary to carry out additional measurements or structure refinements. However, the purpose of your study may justify the reported deviations and the more serious of these should normally be commented upon in the discussion or experimental section of a paper or in the "special_details" fields of the CIF. checkCIF was carefully designed to identify outliers and unusual parameters, but every test has its limitations and alerts that are not important in a particular case may appear. Conversely, the absence of alerts does not guarantee there are no aspects of the results needing attention. It is up to the individual to critically assess their own results and, if necessary, seek expert advice.

Publication of your CIF in IUCr journals

A basic structural check has been run on your CIF. These basic checks will be run on all CIFs submitted for publication in IUCr journals (*Acta Crystallographica*, *Journal of Applied Crystallography*, *Journal of Synchrotron Radiation*); however, if you intend to submit to *Acta Crystallographica Section C* or *E* or *IUCrData*, you should make sure that full publication checks are run on the final version of your CIF prior to submission.

Publication of your CIF in other journals

Please refer to the *Notes for Authors* of the relevant journal for any special instructions relating to CIF submission.

PLATON version of 28/11/2022; check.def file version of 28/11/2022

Datablock cageBr - ellipsoid plot

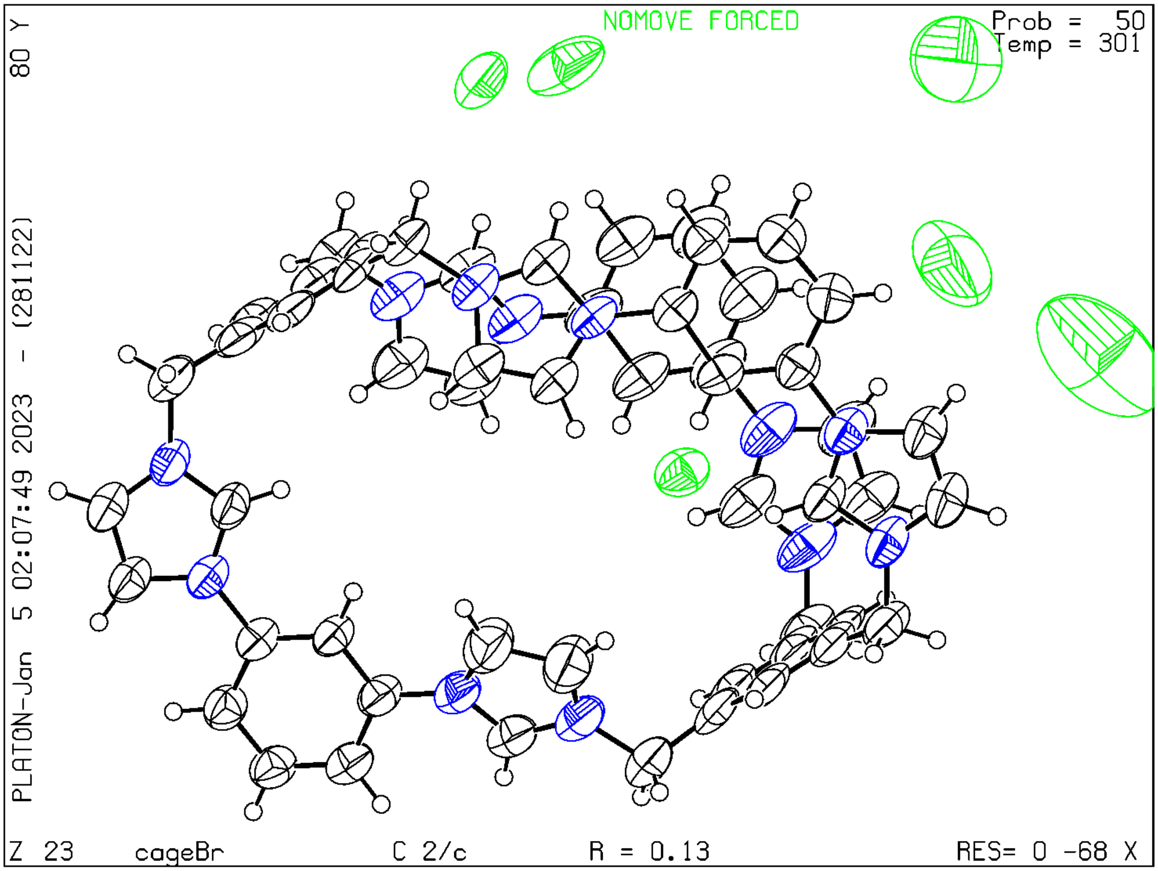

checkCIF/PLATON report

Structure factors have been supplied for datablock(s) cageCl

THIS REPORT IS FOR GUIDANCE ONLY. IF USED AS PART OF A REVIEW PROCEDURE FOR PUBLICATION, IT SHOULD NOT REPLACE THE EXPERTISE OF AN EXPERIENCED CRYSTALLOGRAPHIC REFEREE.

No syntax errors found. CIF dictionary Interpreting this report

Datablock: cageCl

Bond precision: C-C = 0.0053 A Wavelength=1.34050

Cell:	a=13.6528(7)	b=22.9332(10)	c=19.5435(11)
	alpha=90	beta=106.068(6)	gamma=90
Temperature:	100 K		

	Calculated	Reported
Volume	5880.1(5)	5880.1(5)
Space group	P 21/c	P 1 21/c 1
Hall group	-P 2ybc	-P 2ybc
Moiety formula	C54 H48 N12, 6(Cl) [+ solvent]	6(Cl), C54 H48 N12
Sum formula	C54 H48 Cl6 N12 [+ solvent]	C54 H48 Cl6 N12
Mr	1077.74	1077.74
Dx,g cm-3	1.217	1.217
Z	4	4
Mu (mm-1)	2.000	1.997
F000	2232.0	2232.0
F000'	2243.61	
h,k,lmax	17,29,25	17,29,25
Nref	13396	12967
Tmin,Tmax	0.733,0.819	
Tmin'	0.639	

Correction method= Not given

Data completeness= 0.968 Theta(max)= 60.289

R(reflections)= 0.0744(10594) wR2(reflections)= 0.2051(12967)

S = 1.015 Npar= 678

The following ALERTS were generated. Each ALERT has the format
test-name_ALERT_alert-type_alert-level.
Click on the hyperlinks for more details of the test.

Alert level B

```
PLAT260_ALERT_2_B Large Average Ueq of Residue Including      Cl5B      0.348 Check
```

Author Response: This is due to highly disorder of counteranion Cl5B and Cl8

```
PLAT260_ALERT_2_B Large Average Ueq of Residue Including       Cl8      0.559 Check
```

Author Response: This is due to highly disorder of counteranion Cl5B and Cl8

Alert level C

```
RADNW01_ALERT_1_C  The radiation wavelength lies outside the expected range
            for the supplied radiation type. Expected range 1.34130-1.34150
            Wavelength given =    1.34050
PLAT031_ALERT_4_C Refined Extinction Parameter Within Range of ...      3.250 Sigma
PLAT052_ALERT_1_C Info on Absorption Correction Method   Not Given     Please Do !
PLAT057_ALERT_3_C Correction for Absorption Required   RT(exp) ...       1.12 Do !
PLAT340_ALERT_3_C Low Bond Precision on  C-C Bonds ...............    0.00529 Ang.
PLAT906_ALERT_3_C Large K Value in the Analysis of Variance ......      8.269 Check
PLAT906_ALERT_3_C Large K Value in the Analysis of Variance ......      2.075 Check
PLAT911_ALERT_3_C Missing FCF Refl Between Thmin & STh/L=    0.600          4 Report
PLAT934_ALERT_3_C Number of (Iobs-Icalc)/Sigma(W) > 10 Outliers ..          1 Check
```

Alert level G

```
ABSMU01_ALERT_1_G  Calculation of _exptl_absorpt_correction_mu
                not performed for this radiation type.
PLAT042_ALERT_1_G Calc. and Reported MoietyFormula Strings  Differ     Please Check
PLAT083_ALERT_2_G SHELXL Second Parameter in WGHT  Unusually Large      12.60 Why ?
PLAT300_ALERT_4_G Atom Site Occupancy of Cl8        Constrained at     0.3333 Check
PLAT300_ALERT_4_G Atom Site Occupancy of Cl8B       Constrained at     0.3333 Check
PLAT300_ALERT_4_G Atom Site Occupancy of Cl8C       Constrained at     0.3333 Check
PLAT302_ALERT_4_G Anion/Solvent/Minor-Residue Disorder (Resd  6  )       100% Note
PLAT302_ALERT_4_G Anion/Solvent/Minor-Residue Disorder (Resd  7  )       100% Note
PLAT302_ALERT_4_G Anion/Solvent/Minor-Residue Disorder (Resd  8  )       100% Note
PLAT302_ALERT_4_G Anion/Solvent/Minor-Residue Disorder (Resd  9  )       100% Note
PLAT302_ALERT_4_G Anion/Solvent/Minor-Residue Disorder (Resd 10  )       100% Note
PLAT304_ALERT_4_G Non-Integer Number of Atoms in ..... (Resd  6  )       0.50 Check
PLAT304_ALERT_4_G Non-Integer Number of Atoms in ..... (Resd  7  )       0.50 Check
PLAT304_ALERT_4_G Non-Integer Number of Atoms in ..... (Resd  8  )       0.33 Check
PLAT304_ALERT_4_G Non-Integer Number of Atoms in ..... (Resd  9  )       0.33 Check
PLAT304_ALERT_4_G Non-Integer Number of Atoms in ..... (Resd 10  )       0.33 Check
PLAT432_ALERT_2_G Short Inter X...Y Contact  Cl5B      ..C23_2    .       3.24 Ang.
                                                        x,y,z  =      1_555 Check
PLAT432_ALERT_2_G Short Inter X...Y Contact  Cl8B      ..C47_2    .       3.08 Ang.
                                                        x,y,z  =      1_555 Check
PLAT605_ALERT_4_G Largest Solvent Accessible VOID in the Structure        283 A**3
```

```
PLAT720_ALERT_4_G Number of Unusual/Non-Standard Labels ..........        111 Note
PLAT790_ALERT_4_G Centre of Gravity not Within Unit Cell: Resd.  #          3 Note
              Cl
PLAT869_ALERT_4_G ALERTS Related to the Use of SQUEEZE  Suppressed          ! Info
PLAT883_ALERT_1_G No Info/Value for _atom_sites_solution_primary .     Please Do !
PLAT912_ALERT_4_G Missing # of FCF Reflections Above STh/L=  0.600        426 Note
PLAT941_ALERT_3_G Average HKL Measurement Multiplicity ..........         3.4 Low
PLAT978_ALERT_2_G Number C-C Bonds with Positive Residual Density.          1 Info
PLAT984_ALERT_1_G The  C-f'=    0.0147 Deviates from the B&C-Value     0.0137 Check
PLAT984_ALERT_1_G The Cl-f'=    0.3293 Deviates from the B&C-Value     0.3281 Check
PLAT984_ALERT_1_G The  N-f'=    0.0256 Deviates from the B&C-Value     0.0241 Check
PLAT985_ALERT_1_G The Cl-f"=    0.5493 Deviates from the B&C-Value     0.5435 Check
```

```
  0 ALERT level A = Most likely a serious problem - resolve or explain
  2 ALERT level B = A potentially serious problem, consider carefully
  9 ALERT level C = Check. Ensure it is not caused by an omission or oversight
 30 ALERT level G = General information/check it is not something unexpected

  9 ALERT type 1 CIF construction/syntax error, inconsistent or missing data
  6 ALERT type 2 Indicator that the structure model may be wrong or deficient
  7 ALERT type 3 Indicator that the structure quality may be low
 19 ALERT type 4 Improvement, methodology, query or suggestion
  0 ALERT type 5 Informative message, check
```

It is advisable to attempt to resolve as many as possible of the alerts in all categories. Often the minor alerts point to easily fixed oversights, errors and omissions in your CIF or refinement strategy, so attention to these fine details can be worthwhile. In order to resolve some of the more serious problems it may be necessary to carry out additional measurements or structure refinements. However, the purpose of your study may justify the reported deviations and the more serious of these should normally be commented upon in the discussion or experimental section of a paper or in the "special_details" fields of the CIF. checkCIF was carefully designed to identify outliers and unusual parameters, but every test has its limitations and alerts that are not important in a particular case may appear. Conversely, the absence of alerts does not guarantee there are no aspects of the results needing attention. It is up to the individual to critically assess their own results and, if necessary, seek expert advice.

Publication of your CIF in IUCr journals

A basic structural check has been run on your CIF. These basic checks will be run on all CIFs submitted for publication in IUCr journals (*Acta Crystallographica*, *Journal of Applied Crystallography*, *Journal of Synchrotron Radiation*); however, if you intend to submit to *Acta Crystallographica Section C* or *E* or *IUCrData*, you should make sure that full publication checks are run on the final version of your CIF prior to submission.

Publication of your CIF in other journals

Please refer to the *Notes for Authors* of the relevant journal for any special instructions relating to CIF submission.

PLATON version of 28/11/2022; check.def file version of 28/11/2022

Datablock cageCl - ellipsoid plot

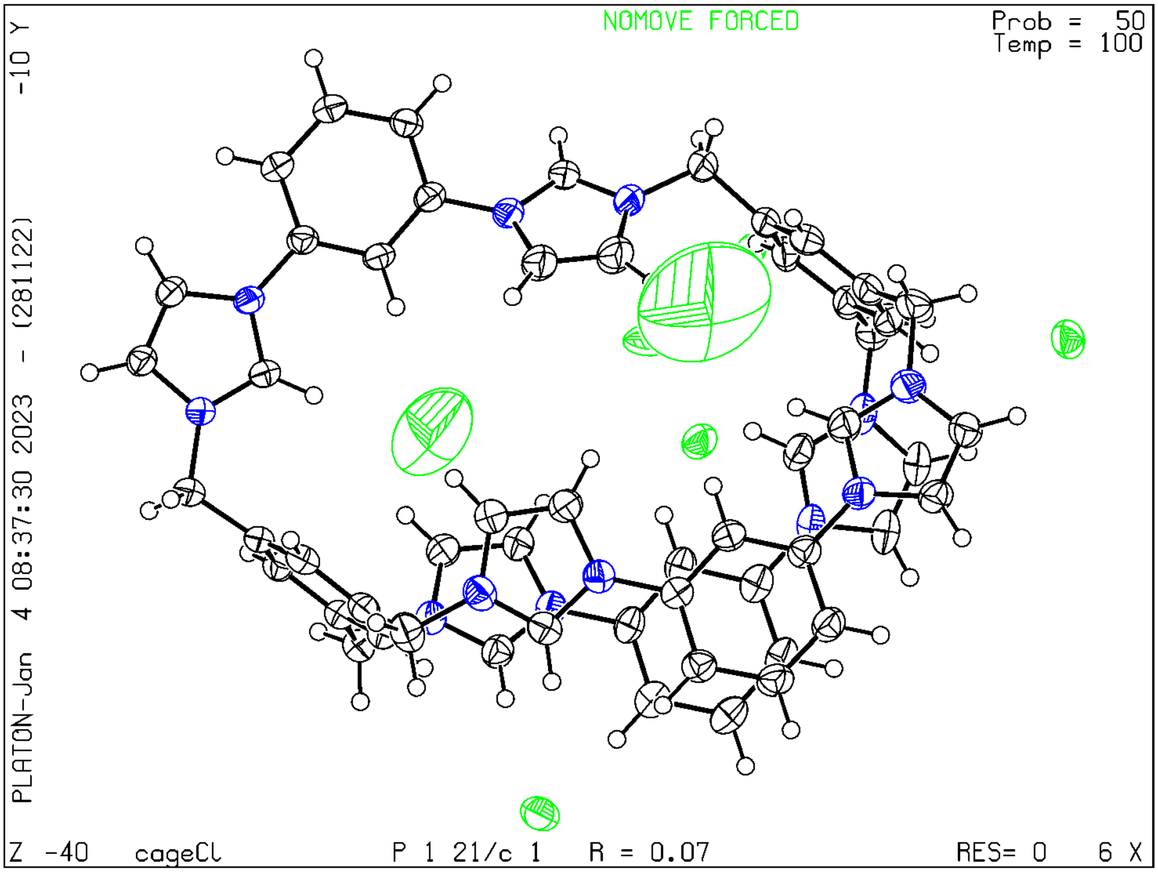

checkCIF/PLATON report

Structure factors have been supplied for datablock(s) cageI

THIS REPORT IS FOR GUIDANCE ONLY. IF USED AS PART OF A REVIEW PROCEDURE FOR PUBLICATION, IT SHOULD NOT REPLACE THE EXPERTISE OF AN EXPERIENCED CRYSTALLOGRAPHIC REFEREE.

No syntax errors found. CIF dictionary Interpreting this report

Datablock: cageI

Bond precision: C-C = 0.0114 A Wavelength=1.34050

Cell:	a=14.03844(17)	b=21.1682(2)	c=22.3833(2)
	alpha=75.0123(10)	beta=85.6441(9)	gamma=86.4936(9)
Temperature:	150 K		

	Calculated	Reported
Volume	6400.82(12)	6400.84(13)
Space group	P -1	P -1
Hall group	-P 1	-P 1
Moiety formula	C54 H48 N12, C2 H6 O S, 6(I), H2 O [+ solvent]	6(I), C54 H48 N12, H2 O, C2 H6 O S
Sum formula	C56 H56 I6 N12 O2 S [+ solvent]	C56 H56 I6 N12 O2 S
Mr	1722.59	1722.58
Dx,g cm-3	1.788	1.788
Z	4	4
Mu (mm-1)	16.171	16.171
F000	3304.0	3304.0
F000'	3312.07	
h,k,lmax	16,25,26	16,25,26
Nref	22623	22605
Tmin,Tmax	0.587,0.724	0.465,1.000
Tmin'	0.499	

Correction method= # Reported T Limits: Tmin=0.465 Tmax=1.000
AbsCorr = MULTI-SCAN

Data completeness= 0.999 Theta(max)= 52.929

R(reflections)= 0.0592(19970)

wR2(reflections)= 0.1844(22605)

S = 1.036 Npar= 1461

The following ALERTS were generated. Each ALERT has the format
test-name_ALERT_alert-type_alert-level.
Click on the hyperlinks for more details of the test.

Alert level A

PLAT972_ALERT_2_A Check Calcd Resid. Dens. 0.44Ang From I12 -5.73 eA-3

Author Response: These alerts are caused by ghost peaks around heavy atoms.

PLAT972_ALERT_2_A Check Calcd Resid. Dens. 0.48Ang From I12 -5.44 eA-3

Author Response: These alerts are caused by ghost peaks around heavy atoms.

Alert level B

DIFMN02_ALERT_2_B The minimum difference density is < -0.1*ZMAX*1.00
_refine_diff_density_min given = -5.746
Test value = -5.300

Author Response: These alerts are caused by ghost peaks around heavy atoms.

PLAT098_ALERT_2_B Large Reported Min. (Negative) Residual Density -5.75 eA-3

Author Response: This alert are caused by ghost peaks around heavy atoms.

PLAT196_ALERT_1_B No TEMP record and _measurement_temperature .NE. 293 Degree
PLAT971_ALERT_2_B Check Calcd Resid. Dens. 0.15Ang From I11 3.50 eA-3

Author Response: This alert are caused by ghost peaks around heavy atoms.

Alert level C

ABSTY02_ALERT_1_C An _exptl_absorpt_correction_type has been given without
a literature citation. This should be contained in the
_exptl_absorpt_process_details field.
Absorption correction given as multi-scan
DIFMN03_ALERT_1_C The minimum difference density is < -0.1*ZMAX*0.75
The relevant atom site should be identified.
RADNW01_ALERT_1_C The radiation wavelength lies outside the expected range
for the supplied radiation type. Expected range 1.34130-1.34150
Wavelength given = 1.34050

```
PLAT241_ALERT_2_C High   'MainMol' Ueq as Compared to Neighbors of        C3V Check
PLAT243_ALERT_4_C High   'Solvent' Ueq as Compared to Neighbors of        O44 Check
PLAT243_ALERT_4_C High   'Solvent' Ueq as Compared to Neighbors of        C43 Check
PLAT243_ALERT_4_C High   'Solvent' Ueq as Compared to Neighbors of        C46 Check
PLAT250_ALERT_2_C Large U3/U1 Ratio for Average U(i,j) Tensor ....        2.3 Note
PLAT342_ALERT_3_C Low Bond Precision on  C-C Bonds ..............    0.01144 Ang.
PLAT767_ALERT_4_C INS Embedded LIST 6 Instruction Should be LIST 4     Please Check
PLAT906_ALERT_3_C Large K Value in the Analysis of Variance ......      2.676 Check
PLAT911_ALERT_3_C Missing FCF Refl Between Thmin & STh/L=    0.595         18 Report
PLAT971_ALERT_2_C Check Calcd Resid. Dens.  0.10Ang From I2            2.32 eA-3
```

Author Response: This alert are caused by ghost peaks around heavy atoms.

```
PLAT971_ALERT_2_C Check Calcd Resid. Dens.  0.80Ang From I9            2.31 eA-3
```

Author Response: This alert are caused by ghost peaks around heavy atoms.

```
PLAT971_ALERT_2_C Check Calcd Resid. Dens.  0.89Ang From I1A           2.23 eA-3
```

Author Response: This alert are caused by ghost peaks around heavy atoms.

```
PLAT971_ALERT_2_C Check Calcd Resid. Dens.  0.91Ang From I5            2.05 eA-3
```

Author Response: This alert are caused by ghost peaks around heavy atoms.

```
PLAT971_ALERT_2_C Check Calcd Resid. Dens.  1.46Ang From I1B           2.02 eA-3
```

Author Response: This alert are caused by ghost peaks around heavy atoms.

```
PLAT971_ALERT_2_C Check Calcd Resid. Dens.  0.60Ang From I4            1.96 eA-3
```

Author Response: This alert are caused by ghost peaks around heavy atoms.

```
PLAT971_ALERT_2_C Check Calcd Resid. Dens.  0.79Ang From I10           1.91 eA-3
```

Author Response: This alert are caused by ghost peaks around heavy atoms.

```
PLAT971_ALERT_2_C Check Calcd Resid. Dens.  0.14Ang From I4            1.90 eA-3
```

Author Response: This alert are caused by ghost peaks around heavy atoms.

```
PLAT971_ALERT_2_C Check Calcd Resid. Dens.  0.54Ang From I9            1.88 eA-3
```

Author Response: This alert are caused by ghost peaks around heavy atoms.

Alert level G

```
ABSMU01_ALERT_1_G  Calculation of _exptl_absorpt_correction_mu
                not performed for this radiation type.
PLAT003_ALERT_2_G Number of Uiso or Uij Restrained non-H Atoms ...          1 Report
PLAT007_ALERT_5_G Number of Unrefined Donor-H Atoms .............          4 Report
PLAT017_ALERT_1_G Check Scattering Type Consistency of C6       as         N
PLAT042_ALERT_1_G Calc. and Reported MoietyFormula Strings  Differ     Please Check
PLAT072_ALERT_2_G SHELXL First  Parameter in WGHT  Unusually Large       0.12 Report
PLAT083_ALERT_2_G SHELXL Second Parameter in WGHT  Unusually Large      46.96 Why ?
PLAT177_ALERT_4_G The CIF-Embedded .res File Contains DELU Records          1 Report
PLAT178_ALERT_4_G The CIF-Embedded .res File Contains SIMU Records          1 Report
PLAT186_ALERT_4_G The CIF-Embedded .res File Contains ISOR Records          1 Report
PLAT302_ALERT_4_G Anion/Solvent/Minor-Residue Disorder (Resd  3  )        50% Note
PLAT302_ALERT_4_G Anion/Solvent/Minor-Residue Disorder (Resd  4  )        25% Note
PLAT302_ALERT_4_G Anion/Solvent/Minor-Residue Disorder (Resd  5  )       100% Note
PLAT302_ALERT_4_G Anion/Solvent/Minor-Residue Disorder (Resd  7  )       100% Note
PLAT302_ALERT_4_G Anion/Solvent/Minor-Residue Disorder (Resd 11  )       100% Note
PLAT302_ALERT_4_G Anion/Solvent/Minor-Residue Disorder (Resd 12  )       100% Note
PLAT302_ALERT_4_G Anion/Solvent/Minor-Residue Disorder (Resd 17  )       100% Note
PLAT302_ALERT_4_G Anion/Solvent/Minor-Residue Disorder (Resd 18  )       100% Note
PLAT302_ALERT_4_G Anion/Solvent/Minor-Residue Disorder (Resd 19  )       100% Note
PLAT302_ALERT_4_G Anion/Solvent/Minor-Residue Disorder (Resd 20  )       100% Note
PLAT304_ALERT_4_G Non-Integer Number of Atoms in ..... (Resd  5  )       0.70 Check
PLAT304_ALERT_4_G Non-Integer Number of Atoms in ..... (Resd  7  )       0.70 Check
PLAT304_ALERT_4_G Non-Integer Number of Atoms in ..... (Resd 11  )       0.70 Check
PLAT304_ALERT_4_G Non-Integer Number of Atoms in ..... (Resd 12  )       0.70 Check
PLAT304_ALERT_4_G Non-Integer Number of Atoms in ..... (Resd 17  )       0.30 Check
PLAT304_ALERT_4_G Non-Integer Number of Atoms in ..... (Resd 18  )       0.30 Check
PLAT304_ALERT_4_G Non-Integer Number of Atoms in ..... (Resd 19  )       0.30 Check
PLAT304_ALERT_4_G Non-Integer Number of Atoms in ..... (Resd 20  )       0.30 Check
PLAT431_ALERT_2_G Short Inter HL..A Contact  I12      ..N1       .       3.32 Ang.
                                                     x,y,z  =     1_555 Check
PLAT431_ALERT_2_G Short Inter HL..A Contact  I12      ..N4       .       3.42 Ang.
                                                     x,y,z  =     1_555 Check
PLAT432_ALERT_2_G Short Inter X...Y Contact  I12      ..C2P      .       3.30 Ang.
                                                     x,y,z  =     1_555 Check
PLAT432_ALERT_2_G Short Inter X...Y Contact  I12      ..C20      .       3.45 Ang.
                                                     x,y,z  =     1_555 Check
PLAT605_ALERT_4_G Largest Solvent Accessible VOID in the Structure        277 A**3
PLAT720_ALERT_4_G Number of Unusual/Non-Standard Labels ..........         19 Note
PLAT790_ALERT_4_G Centre of Gravity not Within Unit Cell: Resd.  #          4 Note
              C2 H6 O S
PLAT790_ALERT_4_G Centre of Gravity not Within Unit Cell: Resd.  #          7 Note
              I
PLAT790_ALERT_4_G Centre of Gravity not Within Unit Cell: Resd.  #          8 Note
              I
PLAT790_ALERT_4_G Centre of Gravity not Within Unit Cell: Resd.  #          9 Note
              I
PLAT790_ALERT_4_G Centre of Gravity not Within Unit Cell: Resd.  #         15 Note
              I
PLAT790_ALERT_4_G Centre of Gravity not Within Unit Cell: Resd.  #         18 Note
              I
PLAT790_ALERT_4_G Centre of Gravity not Within Unit Cell: Resd.  #         21 Note
              H2 O
PLAT860_ALERT_3_G Number of Least-Squares Restraints ............          6 Note
```

```
PLAT869_ALERT_4_G ALERTS Related to the Use of SQUEEZE  Suppressed          ! Info
PLAT883_ALERT_1_G No Info/Value for _atom_sites_solution_primary .     Please Do !
PLAT909_ALERT_3_G Percentage of I>2sig(I) Data at Theta(Max) Still        77% Note
PLAT910_ALERT_3_G Missing # of FCF Reflection(s) Below Theta(Min).          2 Note
PLAT933_ALERT_2_G Number of HKL-OMIT Records in Embedded .res File         13 Note
PLAT941_ALERT_3_G Average HKL Measurement Multiplicity ..........         3.9 Low
PLAT978_ALERT_2_G Number C-C Bonds with Positive Residual Density.          0 Info
PLAT984_ALERT_1_G The  C-f'=    0.0147 Deviates from the B&C-Value     0.0137 Check
PLAT984_ALERT_1_G The  I-f'=    0.0976 Deviates from the B&C-Value     0.0030 Check
PLAT984_ALERT_1_G The  N-f'=    0.0253 Deviates from the B&C-Value     0.0241 Check
PLAT984_ALERT_1_G The  O-f'=    0.0412 Deviates from the B&C-Value     0.0389 Check
PLAT985_ALERT_1_G The  I-f"=    5.4914 Deviates from the B&C-Value     5.4350 Check
```

```
  2 ALERT level A = Most likely a serious problem - resolve or explain
  4 ALERT level B = A potentially serious problem, consider carefully
 21 ALERT level C = Check. Ensure it is not caused by an omission or oversight
 54 ALERT level G = General information/check it is not something unexpected

 13 ALERT type 1 CIF construction/syntax error, inconsistent or missing data
 25 ALERT type 2 Indicator that the structure model may be wrong or deficient
  7 ALERT type 3 Indicator that the structure quality may be low
 35 ALERT type 4 Improvement, methodology, query or suggestion
  1 ALERT type 5 Informative message, check
```

It is advisable to attempt to resolve as many as possible of the alerts in all categories. Often the minor alerts point to easily fixed oversights, errors and omissions in your CIF or refinement strategy, so attention to these fine details can be worthwhile. In order to resolve some of the more serious problems it may be necessary to carry out additional measurements or structure refinements. However, the purpose of your study may justify the reported deviations and the more serious of these should normally be commented upon in the discussion or experimental section of a paper or in the "special_details" fields of the CIF. checkCIF was carefully designed to identify outliers and unusual parameters, but every test has its limitations and alerts that are not important in a particular case may appear. Conversely, the absence of alerts does not guarantee there are no aspects of the results needing attention. It is up to the individual to critically assess their own results and, if necessary, seek expert advice.

Publication of your CIF in IUCr journals

A basic structural check has been run on your CIF. These basic checks will be run on all CIFs submitted for publication in IUCr journals (*Acta Crystallographica*, *Journal of Applied Crystallography*, *Journal of Synchrotron Radiation*); however, if you intend to submit to *Acta Crystallographica Section C* or *E* or *IUCrData*, you should make sure that full publication checks are run on the final version of your CIF prior to submission.

Publication of your CIF in other journals

Please refer to the *Notes for Authors* of the relevant journal for any special instructions relating to CIF submission.

Validation response form

Please find below a validation response form (VRF) that can be filled in and pasted into your CIF.

```
# start Validation Reply Form
_vrf_PLAT196_cageI
;
PROBLEM: No TEMP record and _measurement_temperature .NE.        293 Degree
RESPONSE: ...
;
# end Validation Reply Form
```

PLATON version of 28/11/2022; check.def file version of 28/11/2022

Datablock cageI - ellipsoid plot

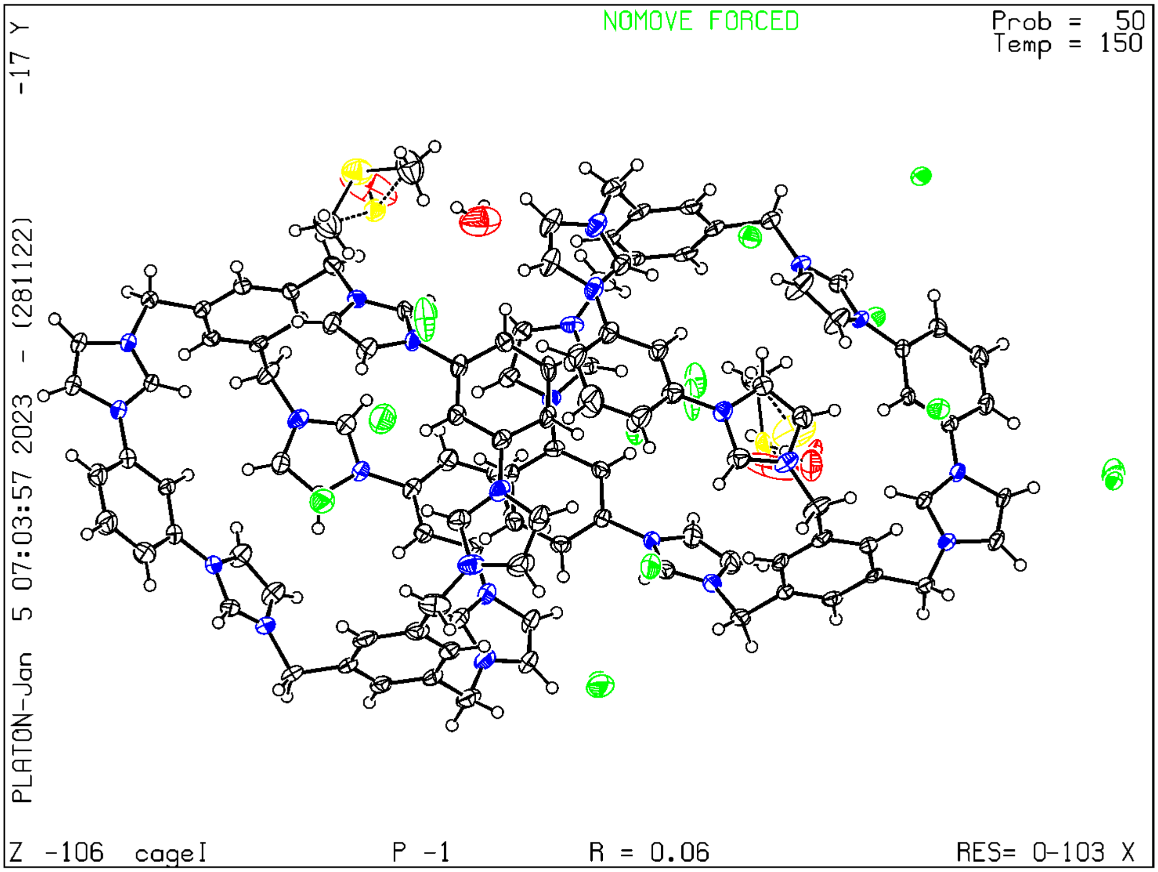

checkCIF/PLATON report

Structure factors have been supplied for datablock(s) cageI4Br

THIS REPORT IS FOR GUIDANCE ONLY. IF USED AS PART OF A REVIEW PROCEDURE FOR PUBLICATION, IT SHOULD NOT REPLACE THE EXPERTISE OF AN EXPERIENCED CRYSTALLOGRAPHIC REFEREE.

No syntax errors found. CIF dictionary Interpreting this report

Datablock: cageI4Br

Bond precision:	C-C = 0.0180 A	Wavelength=1.34139	
Cell:	a=14.23375(19)	b=14.4164(2)	c=19.8789(3)
	alpha=92.8920(12)	beta=95.2333(11)	gamma=94.2796(11)
Temperature:	100 K		

	Calculated	Reported
Volume	4044.07(10)	4044.07(10)
Space group	P -1	P -1
Hall group	-P 1	-P 1
Moiety formula	C54 H48 N12, Br I4, 5(Br I2) [+ solvent]	5(Br I2), Br I4, C54 H48 N12
Sum formula	C54 H48 Br6 I14 N12 [+ solvent]	C54 H48 Br6 I14 N12 [+solvent]
Mr	3121.04	3121.04
Dx,g cm-3	2.563	2.563
Z	2	2
Mu (mm-1)	31.456	31.456
F000	2816.0	2816.0
F000'	2811.51	
h,k,lmax	18,18,25	18,18,25
Nref	17175	17145
Tmin,Tmax	0.009,0.043	0.174,1.000
Tmin'	0.000	

Correction method= # Reported T Limits: Tmin=0.174 Tmax=1.000
AbsCorr = MULTI-SCAN

Data completeness= 0.998 Theta(max)= 58.098

R(reflections)= 0.0845(15387)	wR2(reflections)= 0.2727(17145)
S = 1.072 Npar= 812	

The following ALERTS were generated. Each ALERT has the format
test-name_ALERT_alert-type_alert-level.
Click on the hyperlinks for more details of the test.

Alert level A

PLAT971_ALERT_2_A Check Calcd Resid. Dens. 0.17Ang From Br10 10.42 eA-3

Author Response: These alerts are caused by ghost peaks around heavy atoms.

PLAT971_ALERT_2_A Check Calcd Resid. Dens. 0.35Ang From Br11 7.63 eA-3

Author Response: These alerts are caused by ghost peaks around heavy atoms.

PLAT971_ALERT_2_A Check Calcd Resid. Dens. 0.36Ang From Br13 6.87 eA-3

Author Response: These alerts are caused by ghost peaks around heavy atoms.

PLAT971_ALERT_2_A Check Calcd Resid. Dens. 0.17Ang From Br1A 5.36 eA-3

Author Response: These alerts are caused by ghost peaks around heavy atoms.

PLAT972_ALERT_2_A Check Calcd Resid. Dens. 0.65Ang From I19 -5.07 eA-3

Author Response: These alerts are caused by ghost peaks around heavy atoms.

PLAT972_ALERT_2_A Check Calcd Resid. Dens. 0.41Ang From I19 -4.82 eA-3

Author Response: These alerts are caused by ghost peaks around heavy atoms.

PLAT972_ALERT_2_A Check Calcd Resid. Dens. 0.70Ang From I19 -4.58 eA-3

Author Response: These alerts are caused by ghost peaks around heavy atoms.

PLAT972_ALERT_2_A Check Calcd Resid. Dens. 0.55Ang From I15 -4.51 eA-3

Author Response: These alerts are caused by ghost peaks around heavy atoms.

PLAT972_ALERT_2_A Check Calcd Resid. Dens. 0.62Ang From I11   -4.17 eA-3

Author Response: These alerts are caused by ghost peaks around heavy atoms.

PLAT972_ALERT_2_A Check Calcd Resid. Dens. 0.29Ang From I15   -4.07 eA-3

Author Response: These alerts are caused by ghost peaks around heavy atoms.

PLAT972_ALERT_2_A Check Calcd Resid. Dens. 0.49Ang From I15   -4.05 eA-3

Author Response: These alerts are caused by ghost peaks around heavy atoms.

PLAT972_ALERT_2_A Check Calcd Resid. Dens. 0.58Ang From I11   -3.99 eA-3

Author Response: These alerts are caused by ghost peaks around heavy atoms.

PLAT972_ALERT_2_A Check Calcd Resid. Dens. 0.63Ang From I15   -3.84 eA-3

Author Response: These alerts are caused by ghost peaks around heavy atoms.

PLAT972_ALERT_2_A Check Calcd Resid. Dens. 0.50Ang From I11   -3.59 eA-3

Author Response: These alerts are caused by ghost peaks around heavy atoms.

Alert level B

PLAT097_ALERT_2_B Large Reported Max. (Positive) Residual Density   10.15 eA-3

Author Response: This alert are caused by ghost peaks around heavy atoms.

PLAT196_ALERT_1_B No TEMP record and _measurement_temperature .NE.   293 Degree
PLAT971_ALERT_2_B Check Calcd Resid. Dens. 0.75Ang From I1   3.09 eA-3

Author Response: These alerts are caused by ghost peaks around heavy atoms.

PLAT971_ALERT_2_B Check Calcd Resid. Dens. 0.99Ang From I12   2.73 eA-3

Author Response: These alerts are caused by ghost peaks around heavy atoms.

PLAT971_ALERT_2_B Check Calcd Resid. Dens. 0.49Ang From I8   2.64 eA-3

Author Response: These alerts are caused by ghost peaks around heavy atoms.

```
PLAT971_ALERT_2_B Check Calcd Resid. Dens.  0.95Ang From I4             2.62 eA-3
```

Author Response: These alerts are caused by ghost peaks around heavy atoms.

```
PLAT971_ALERT_2_B Check Calcd Resid. Dens.  0.07Ang From I2             2.58 eA-3
```

Author Response: These alerts are caused by ghost peaks around heavy atoms.

Alert level C

```
DIFMN02_ALERT_2_C  The minimum difference density is < -0.1*ZMAX*0.75
            _refine_diff_density_min given =     -4.890
            Test value =     -3.975
DIFMN03_ALERT_1_C  The minimum difference density is < -0.1*ZMAX*0.75
            The relevant atom site should be identified.
DIFMX02_ALERT_1_C  The maximum difference density is > 0.1*ZMAX*0.75
            The relevant atom site should be identified.
PLAT084_ALERT_3_C High wR2 Value (i.e. > 0.25) ..................       0.27 Report
PLAT094_ALERT_2_C Ratio of Maximum / Minimum Residual Density ....       2.08 Report
PLAT098_ALERT_2_C Large Reported Min.  (Negative) Residual Density      -4.89 eA-3
PLAT244_ALERT_4_C Low    'Solvent' Ueq as Compared to Neighbors of       Br11 Check
PLAT244_ALERT_4_C Low    'Solvent' Ueq as Compared to Neighbors of         I3 Check
PLAT244_ALERT_4_C Low    'Solvent' Ueq as Compared to Neighbors of         I4 Check
PLAT342_ALERT_3_C Low Bond Precision on  C-C Bonds ..............      0.018 Ang.
PLAT767_ALERT_4_C INS Embedded LIST 6 Instruction Should be LIST 4     Please Check
PLAT790_ALERT_4_C Centre of Gravity not Within Unit Cell: Resd.  #          1 Note
             C54 H48 N12
PLAT906_ALERT_3_C Large K Value in the Analysis of Variance ......      2.251 Check
PLAT911_ALERT_3_C Missing FCF Refl Between Thmin & STh/L=    0.600          8 Report
PLAT918_ALERT_3_C Reflection(s) with I(obs) much Smaller I(calc) .          7 Check
PLAT971_ALERT_2_C Check Calcd Resid. Dens.  0.97Ang From I13            2.46 eA-3
```

Author Response: These alerts are caused by ghost peaks around heavy atoms.

```
PLAT971_ALERT_2_C Check Calcd Resid. Dens.  0.86Ang From I8             2.45 eA-3
```

Author Response: These alerts are caused by ghost peaks around heavy atoms.

```
PLAT971_ALERT_2_C Check Calcd Resid. Dens.  0.80Ang From I1             2.42 eA-3
```

Author Response: These alerts are caused by ghost peaks around heavy atoms.

```
PLAT971_ALERT_2_C Check Calcd Resid. Dens.  0.88Ang From I2             2.40 eA-3
```

Author Response: These alerts are caused by ghost peaks around heavy atoms.

PLAT971_ALERT_2_C Check Calcd Resid. Dens. 1.02Ang From I3 2.35 eA-3

Author Response: These alerts are caused by ghost peaks around heavy atoms.

PLAT971_ALERT_2_C Check Calcd Resid. Dens. 0.83Ang From I2 2.31 eA-3

Author Response: These alerts are caused by ghost peaks around heavy atoms.

PLAT971_ALERT_2_C Check Calcd Resid. Dens. 0.79Ang From I1 2.26 eA-3

Author Response: These alerts are caused by ghost peaks around heavy atoms.

PLAT971_ALERT_2_C Check Calcd Resid. Dens. 0.79Ang From I4 2.23 eA-3

Author Response: These alerts are caused by ghost peaks around heavy atoms.

PLAT971_ALERT_2_C Check Calcd Resid. Dens. 1.00Ang From I4 2.22 eA-3

Author Response: These alerts are caused by ghost peaks around heavy atoms.

PLAT971_ALERT_2_C Check Calcd Resid. Dens. 0.86Ang From I5 2.15 eA-3

Author Response: These alerts are caused by ghost peaks around heavy atoms.

PLAT971_ALERT_2_C Check Calcd Resid. Dens. 1.03Ang From I3 2.15 eA-3

Author Response: These alerts are caused by ghost peaks around heavy atoms.

PLAT971_ALERT_2_C Check Calcd Resid. Dens. 0.79Ang From Br12 2.12 eA-3

Author Response: These alerts are caused by ghost peaks around heavy atoms.

PLAT971_ALERT_2_C Check Calcd Resid. Dens. 0.91Ang From I4 2.11 eA-3

Author Response: These alerts are caused by ghost peaks around heavy atoms.

PLAT971_ALERT_2_C Check Calcd Resid. Dens. 0.76Ang From I8 2.08 eA-3

Author Response: These alerts are caused by ghost peaks around heavy atoms.

```
PLAT971_ALERT_2_C Check Calcd Resid. Dens.  0.79Ang From I8             2.01 eA-3
```

Author Response: These alerts are caused by ghost peaks around heavy atoms.

```
PLAT971_ALERT_2_C Check Calcd Resid. Dens.  0.95Ang From I14            1.96 eA-3
```

Author Response: These alerts are caused by ghost peaks around heavy atoms.

```
PLAT972_ALERT_2_C Check Calcd Resid. Dens.  0.85Ang From I14           -1.96 eA-3
```

Author Response: These alerts are caused by ghost peaks around heavy atoms.

```
PLAT972_ALERT_2_C Check Calcd Resid. Dens.  0.46Ang From I12           -1.89 eA-3
```

Author Response: These alerts are caused by ghost peaks around heavy atoms.

```
PLAT972_ALERT_2_C Check Calcd Resid. Dens.  0.20Ang From C33_1         -1.88 eA-3
```

Author Response: These alerts are caused by ghost peaks around heavy atoms.

```
PLAT972_ALERT_2_C Check Calcd Resid. Dens.  0.34Ang From C63_1         -1.71 eA-3
```

Author Response: These alerts are caused by ghost peaks around heavy atoms.

```
PLAT972_ALERT_2_C Check Calcd Resid. Dens.  0.50Ang From I13           -1.61 eA-3
```

Author Response: These alerts are caused by ghost peaks around heavy atoms.

```
PLAT972_ALERT_2_C Check Calcd Resid. Dens.  0.63Ang From I3            -1.61 eA-3
```

Author Response: These alerts are caused by ghost peaks around heavy atoms.

```
PLAT977_ALERT_2_C Check Negative Difference Density on H16_1     .      -0.39 eA-3
PLAT977_ALERT_2_C Check Negative Difference Density on H42_1     .      -0.35 eA-3
PLAT977_ALERT_2_C Check Negative Difference Density on H55_1     .      -0.36 eA-3
PLAT977_ALERT_2_C Check Negative Difference Density on H63_1     .      -0.32 eA-3
```

Alert level G

```
ABSMU01_ALERT_1_G  Calculation of _exptl_absorpt_correction_mu
                not performed for this radiation type.
CELLZ01_ALERT_1_G Difference between formula and atom_site contents detected.
CELLZ01_ALERT_1_G ALERT: Large difference may be due to a
            symmetry error - see SYMMG tests
           From the CIF: _cell_formula_units_Z    2
           From the CIF: _chemical_formula_sum  C54 H48 Br6 I14 N12 [+solvent]
```

```
TEST: Compare cell contents of formula and atom_site data

atom    Z*formula  cif sites diff
C        108.00    108.00    0.00
H         96.00     96.00    0.00
Br        12.00     12.00    0.00
I         28.00     28.00    0.00
N         24.00     24.00    0.00
[+sol      2.00      0.00    2.00
```

```
PLAT003_ALERT_2_G Number of Uiso or Uij Restrained non-H Atoms ...         57 Report
PLAT042_ALERT_1_G Calc. and Reported MoietyFormula Strings  Differ     Please Check
PLAT072_ALERT_2_G SHELXL First  Parameter in WGHT  Unusually Large       0.19 Report
PLAT083_ALERT_2_G SHELXL Second Parameter in WGHT  Unusually Large      68.27 Why ?
PLAT178_ALERT_4_G The CIF-Embedded .res File Contains SIMU Records          1 Report
PLAT188_ALERT_3_G A Non-default SIMU Restraint Value has been used     0.0100 Report
PLAT301_ALERT_3_G Main Residue  Disorder .............(Resd  1  )          5% Note
PLAT302_ALERT_4_G Anion/Solvent/Minor-Residue Disorder (Resd  3  )        33% Note
PLAT605_ALERT_4_G Largest Solvent Accessible VOID in the Structure         93 A**3
PLAT720_ALERT_4_G Number of Unusual/Non-Standard Labels ..........        120 Note
PLAT790_ALERT_4_G Centre of Gravity not Within Unit Cell: Resd.  #          3 Note
              Br I2
PLAT790_ALERT_4_G Centre of Gravity not Within Unit Cell: Resd.  #          6 Note
              Br I2
PLAT860_ALERT_3_G Number of Least-Squares Restraints .............       1344 Note
PLAT869_ALERT_4_G ALERTS Related to the Use of SQUEEZE  Suppressed          ! Info
PLAT883_ALERT_1_G No Info/Value for _atom_sites_solution_primary .     Please Do !
PLAT910_ALERT_3_G Missing # of FCF Reflection(s) Below Theta(Min).          1 Note
PLAT912_ALERT_4_G Missing # of FCF Reflections Above STh/L=  0.600         21 Note
PLAT933_ALERT_2_G Number of HKL-OMIT Records in Embedded .res File          7 Note
PLAT941_ALERT_3_G Average HKL Measurement Multiplicity ..........         3.2 Low
PLAT978_ALERT_2_G Number C-C Bonds with Positive Residual Density.          0 Info
```

---

14 **ALERT level A** = Most likely a serious problem - resolve or explain
7 **ALERT level B** = A potentially serious problem, consider carefully
41 **ALERT level C** = Check. Ensure it is not caused by an omission or oversight
23 **ALERT level G** = General information/check it is not something unexpected

8 ALERT type 1 CIF construction/syntax error, inconsistent or missing data
54 ALERT type 2 Indicator that the structure model may be wrong or deficient
10 ALERT type 3 Indicator that the structure quality may be low
13 ALERT type 4 Improvement, methodology, query or suggestion
0 ALERT type 5 Informative message, check

It is advisable to attempt to resolve as many as possible of the alerts in all categories. Often the minor alerts point to easily fixed oversights, errors and omissions in your CIF or refinement strategy, so attention to these fine details can be worthwhile. In order to resolve some of the more serious problems it may be necessary to carry out additional measurements or structure refinements. However, the purpose of your study may justify the reported deviations and the more serious of these should normally be commented upon in the discussion or experimental section of a paper or in the "special_details" fields of the CIF. checkCIF was carefully designed to identify outliers and unusual parameters, but every test has its limitations and alerts that are not important in a particular case may appear. Conversely, the absence of alerts does not guarantee there are no aspects of the results needing attention. It is up to the individual to critically assess their own results and, if necessary, seek expert advice.

Publication of your CIF in IUCr journals

A basic structural check has been run on your CIF. These basic checks will be run on all CIFs submitted for publication in IUCr journals (*Acta Crystallographica*, *Journal of Applied Crystallography*, *Journal of Synchrotron Radiation*); however, if you intend to submit to *Acta Crystallographica Section C* or *E* or *IUCrData*, you should make sure that full publication checks are run on the final version of your CIF prior to submission.

Publication of your CIF in other journals

Please refer to the *Notes for Authors* of the relevant journal for any special instructions relating to CIF submission.

Validation response form

Please find below a validation response form (VRF) that can be filled in and pasted into your CIF.

```
# start Validation Reply Form
_vrf_PLAT196_cageI4Br
;
PROBLEM: No TEMP record and _measurement_temperature .NE.        293 Degree
RESPONSE: ...
;
# end Validation Reply Form
```

PLATON version of 28/11/2022; check.def file version of 28/11/2022

Datablock cageI4Br - ellipsoid plot

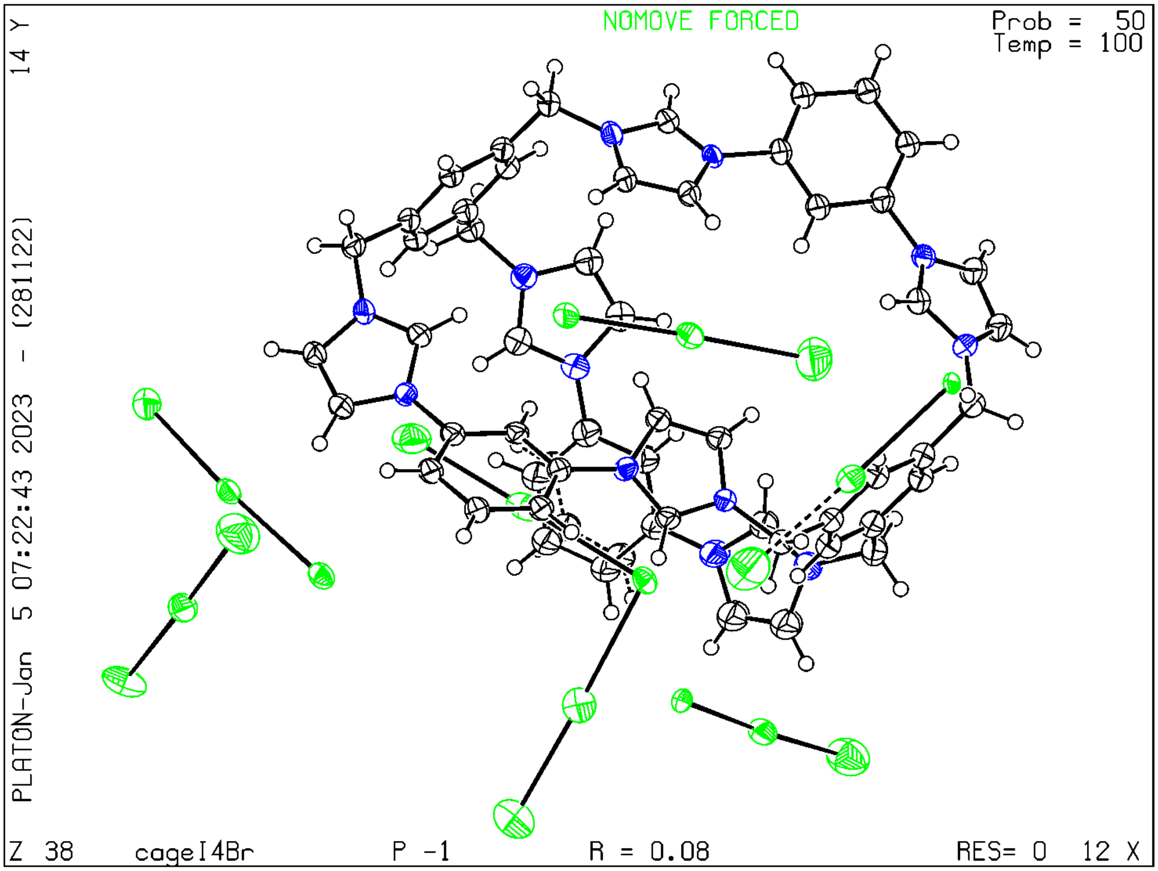

checkCIF/PLATON report

Structure factors have been supplied for datablock(s) cagePF6

THIS REPORT IS FOR GUIDANCE ONLY. IF USED AS PART OF A REVIEW PROCEDURE FOR PUBLICATION, IT SHOULD NOT REPLACE THE EXPERTISE OF AN EXPERIENCED CRYSTALLOGRAPHIC REFEREE.

No syntax errors found. CIF dictionary Interpreting this report

Datablock: cagePF6

Bond precision: C-C = 0.0106 A Wavelength=1.34050

Cell:	a=13.9971(5)	b=23.3167(9)	c=23.5433(11)
	alpha=90	beta=100.018(5)	gamma=90
Temperature:	293 K		

	Calculated	Reported
Volume	7566.6(5)	7566.6(6)
Space group	P 21/n	P 1 21/n 1
Hall group	-P 2yn	-P 2yn
Moiety formula	C54 H48 N12, 6(F6 P) [+ solvent]	C54 H48 N12, 6(F6 P)
Sum formula	C54 H48 F36 N12 P6 [+ solvent]	C54 H48 F36 N12 P6
Mr	1734.86	1734.86
Dx,g cm-3	1.523	1.523
Z	4	4
Mu (mm-1)	1.651	1.638
F000	3480.0	3480.0
F000’	3498.09	
h,k,lmax	14,24,25	14,24,25
Nref	9667	9583
Tmin,Tmax	0.790,0.849	0.594,1.000
Tmin’	0.782	

Correction method= # Reported T Limits: Tmin=0.594 Tmax=1.000
AbsCorr = MULTI-SCAN

Data completeness= 0.991 Theta(max)= 45.757

R(reflections)= 0.1010(7364) wR2(reflections)= 0.2909(9583)

S = 1.043 Npar= 973

The following ALERTS were generated. Each ALERT has the format
test-name_ALERT_alert-type_alert-level.
Click on the hyperlinks for more details of the test.

Alert level A

THETM01_ALERT_3_A The value of sine(theta_max)/wavelength is less than 0.550
Calculated sin(theta_max)/wavelength = 0.5344

Author Response: This is due to weak diffraction of the crystal, and the data at the high angle is poor despite optmized conditions attempted.

Alert level B

PLAT097_ALERT_2_B Large Reported Max. (Positive) Residual Density 1.57 eA-3

Author Response: These alerts are caused by ghost peaks around heavy atoms.

PLAT250_ALERT_2_B Large U3/U1 Ratio for Average U(i,j) Tensor .... 4.3 Note

Author Response: These alerts result from the high level of thermal motion (or minor unresolved disorder) throughout the structure and the use of extensive thermal parameter restraints.

PLAT340_ALERT_3_B Low Bond Precision on C-C Bonds .... 0.01057 Ang.

Author Response: These alerts result from the high level of thermal motion (or minor unresolved disorder) throughout the structure and the use of extensive thermal parameter restraints.

Alert level C

ABSTY02_ALERT_1_C An _exptl_absorpt_correction_type has been given without
a literature citation. This should be contained in the
_exptl_absorpt_process_details field.
Absorption correction given as multi-scan
DIFMX02_ALERT_1_C The maximum difference density is > 0.1*ZMAX*0.75
The relevant atom site should be identified.
RADNW01_ALERT_1_C The radiation wavelength lies outside the expected range
for the supplied radiation type. Expected range 1.34130-1.34150
Wavelength given = 1.34050
PLAT084_ALERT_3_C High wR2 Value (i.e. > 0.25) .... 0.29 Report

```
PLAT088_ALERT_3_C Poor Data / Parameter Ratio ...................       9.85 Note
PLAT260_ALERT_2_C Large Average Ueq of Residue Including      P1_1      0.122 Check
PLAT260_ALERT_2_C Large Average Ueq of Residue Including      P1_4      0.110 Check
PLAT260_ALERT_2_C Large Average Ueq of Residue Including      P1_6      0.224 Check
PLAT334_ALERT_2_C Small <C-C> Benzene Dist.   C1J      -C24      .       1.37 Ang.
PLAT767_ALERT_4_C INS Embedded LIST 6 Instruction Should be LIST 4     Please Check
PLAT906_ALERT_3_C Large K Value in the Analysis of Variance ......      8.402 Check
PLAT906_ALERT_3_C Large K Value in the Analysis of Variance ......      2.648 Check
PLAT906_ALERT_3_C Large K Value in the Analysis of Variance ......      2.053 Check
PLAT911_ALERT_3_C Missing FCF Refl Between Thmin & STh/L=    0.534         83 Report
PLAT971_ALERT_2_C Check Calcd Resid. Dens.  1.61Ang From F2_6           1.58 eA-3
```

Alert level G

```
ABSMU01_ALERT_1_G  Calculation of _exptl_absorpt_correction_mu
                not performed for this radiation type.
PLAT002_ALERT_2_G Number of Distance or Angle Restraints on AtSite         42 Note
PLAT003_ALERT_2_G Number of Uiso or Uij Restrained non-H Atoms ...        108 Report
PLAT072_ALERT_2_G SHELXL First  Parameter in WGHT  Unusually Large       0.15 Report
PLAT083_ALERT_2_G SHELXL Second Parameter in WGHT  Unusually Large      42.14 Why ?
PLAT175_ALERT_4_G The CIF-Embedded .res File Contains SAME Records          5 Report
PLAT177_ALERT_4_G The CIF-Embedded .res File Contains DELU Records          1 Report
PLAT178_ALERT_4_G The CIF-Embedded .res File Contains SIMU Records          1 Report
PLAT199_ALERT_1_G Reported _cell_measurement_temperature ..... (K)        293 Check
PLAT200_ALERT_1_G Reported   _diffrn_ambient_temperature ..... (K)        293 Check
PLAT244_ALERT_4_G Low    'Solvent' Ueq as Compared to Neighbors of       P1_1 Check
PLAT244_ALERT_4_G Low    'Solvent' Ueq as Compared to Neighbors of       P1_2 Check
PLAT244_ALERT_4_G Low    'Solvent' Ueq as Compared to Neighbors of       P1_3 Check
PLAT244_ALERT_4_G Low    'Solvent' Ueq as Compared to Neighbors of       P1_4 Check
PLAT244_ALERT_4_G Low    'Solvent' Ueq as Compared to Neighbors of       P1_5 Check
PLAT244_ALERT_4_G Low    'Solvent' Ueq as Compared to Neighbors of       P1_6 Check
PLAT432_ALERT_2_G Short Inter X...Y Contact  F4_1     ..C2I      .       2.90 Ang.
                                     1/2+x,3/2-y,1/2+z  =      4_676 Check
PLAT432_ALERT_2_G Short Inter X...Y Contact  F2_5     ..C1D      .       2.93 Ang.
                                                 x,y,z  =      1_555 Check
PLAT432_ALERT_2_G Short Inter X...Y Contact  F7_5     ..C1       .       2.82 Ang.
                                    1/2-x,-1/2+y,1/2-z  =      2_545 Check
PLAT432_ALERT_2_G Short Inter X...Y Contact  F7_6     ..C1M      .       2.96 Ang.
                                                 x,y,z  =      1_555 Check
PLAT606_ALERT_4_G Solvent Accessible VOID(S) in Structure ........          ! Info
PLAT720_ALERT_4_G Number of Unusual/Non-Standard Labels ..........         51 Note
PLAT860_ALERT_3_G Number of Least-Squares Restraints ............        1317 Note
PLAT869_ALERT_4_G ALERTS Related to the Use of SQUEEZE  Suppressed          ! Info
PLAT883_ALERT_1_G No Info/Value for _atom_sites_solution_primary .     Please Do !
PLAT909_ALERT_3_G Percentage of I>2sig(I) Data at Theta(Max) Still        53% Note
PLAT913_ALERT_3_G Missing # of Very Strong Reflections in FCF ....          1 Note
PLAT941_ALERT_3_G Average HKL Measurement Multiplicity ...........        4.2 Low
PLAT978_ALERT_2_G Number C-C Bonds with Positive Residual Density.          0 Info
PLAT984_ALERT_1_G The  C-f'=    0.0147 Deviates from the B&C-Value     0.0137 Check
PLAT984_ALERT_1_G The  F-f'=    0.0599 Deviates from the B&C-Value     0.0583 Check
PLAT984_ALERT_1_G The  N-f'=    0.0253 Deviates from the B&C-Value     0.0241 Check
PLAT984_ALERT_1_G The  P-f'=    0.2594 Deviates from the B&C-Value     0.2543 Check
PLAT985_ALERT_1_G The  F-f"=    0.0410 Deviates from the B&C-Value     0.0400 Check
PLAT985_ALERT_1_G The  P-f"=    0.3349 Deviates from the B&C-Value     0.3332 Check
```

---

1 **ALERT level A** = Most likely a serious problem - resolve or explain

```
 3 ALERT level B = A potentially serious problem, consider carefully
15 ALERT level C = Check. Ensure it is not caused by an omission or oversight
35 ALERT level G = General information/check it is not something unexpected

13 ALERT type 1 CIF construction/syntax error, inconsistent or missing data
16 ALERT type 2 Indicator that the structure model may be wrong or deficient
12 ALERT type 3 Indicator that the structure quality may be low
13 ALERT type 4 Improvement, methodology, query or suggestion
 0 ALERT type 5 Informative message, check
```

It is advisable to attempt to resolve as many as possible of the alerts in all categories. Often the minor alerts point to easily fixed oversights, errors and omissions in your CIF or refinement strategy, so attention to these fine details can be worthwhile. In order to resolve some of the more serious problems it may be necessary to carry out additional measurements or structure refinements. However, the purpose of your study may justify the reported deviations and the more serious of these should normally be commented upon in the discussion or experimental section of a paper or in the "special_details" fields of the CIF. checkCIF was carefully designed to identify outliers and unusual parameters, but every test has its limitations and alerts that are not important in a particular case may appear. Conversely, the absence of alerts does not guarantee there are no aspects of the results needing attention. It is up to the individual to critically assess their own results and, if necessary, seek expert advice.

Publication of your CIF in IUCr journals

A basic structural check has been run on your CIF. These basic checks will be run on all CIFs submitted for publication in IUCr journals (*Acta Crystallographica*, *Journal of Applied Crystallography*, *Journal of Synchrotron Radiation*); however, if you intend to submit to *Acta Crystallographica Section C* or *E* or *IUCrData*, you should make sure that full publication checks are run on the final version of your CIF prior to submission.

Publication of your CIF in other journals

Please refer to the *Notes for Authors* of the relevant journal for any special instructions relating to CIF submission.

PLATON version of 28/11/2022; check.def file version of 28/11/2022

Datablock cagePF6 - ellipsoid plot

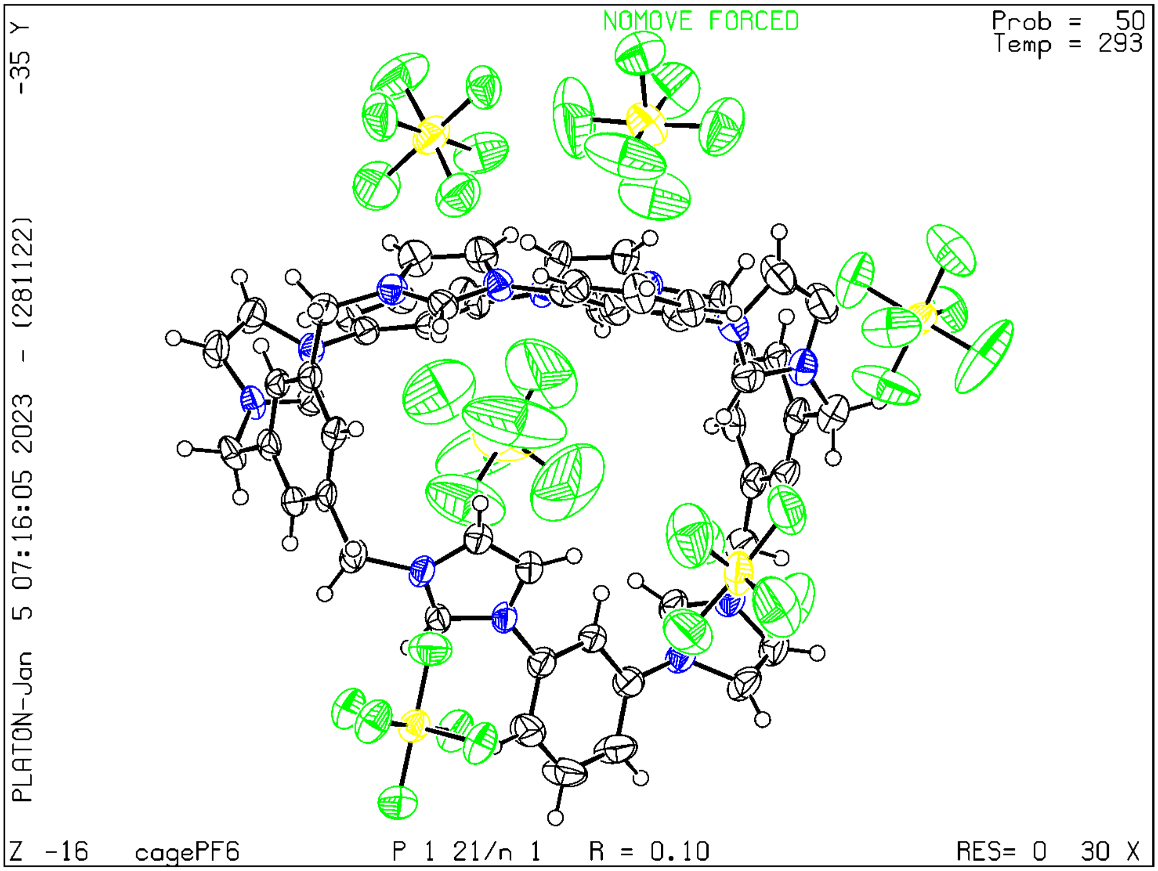

Supplement: Supplementary file 4 — Supplementary Data 1 [file checkcif.pdf › CIF/checkcif.pdf]
